# Supplementary material for: Transforming carbon dioxide into a methanol surrogate using modular transition metal-free Zintl ions
Source: Nat Commun. 2024 Nov 19;15:10030. doi: 10.1038/s41467-024-54277-z (PMC11576849; doi:10.1038/s41467-024-54277-z)
Supplement: Supplementary file 1 — Supplementary Information [file 41467_2024_54277_MOESM1_ESM.pdf]

Supplementary Information

Transforming Carbon Dioxide into a Methanol Surrogate using Modular Transition Metal-Free Zintl Ions

Bono van IJzendoorn,[1] Saad F. Albawardi,[1] William D. Jobbins,[2] George F. S. Whitehead,[2] John E. McGrady,[1]* Meera Mehta[1]*

John.mcgrady@chem.ox.ac.uk, Meera.mehta@chem.ox.ac.uk

1. Department of Chemistry, University of Oxford, 12 Mansfield Road, Oxford, OX1 3QR, U.K.
2. Department of Chemistry, University of Manchester, Oxford Road, Manchester, M13 9PL, U.K.

Table of Contents

1. Methods and Materials 4
1.1. Experimental Considerations 4
1.2. Analytical Considerations 4
1.3. X-ray Diffraction Studies 5
1.4. Synthesis Literature Compounds 6
1.4.1. Synthesis of [Na(DME)$_x$]$_3$P$_7$ 6
1.4.2. Synthesis of K$_3$P$_7$ 7
1.4.3. Synthesis of [K(DME)$_x$]$_3$As$_7$ 7
1.4.4. Synthesis of (Me$_3$Si)$_3$P$_7$ 8
1.4.5. Synthesis of [Na(18c6)]$_2$[HP$_7$] 8
1.4.6. Synthesis of [K(18c6)]$_2$[HP$_7$] 9
1.4.7. Synthesis of [K(18c6)]$_2$[HAs$_7$] 9
1.4.8. Synthesis of [Na(18c6)]$_2$[(BBN)P$_7$] 10
1.4.9. Synthesis of [K(18c6)]$_2$[(BBN)P$_7$] 11
1.4.10. Synthesis of [Na(18c6)]$_2$[(Ph$_2$In)P$_7$] 11
1.4.11. Synthesis of [K(18c6)]$_2$[(Ph$_2$In)P$_7$] 12
2. Synthesis and Characterization of catalysts 13
2.1. Synthesis of [Na(18c6)]$_2$[κ^2-(*i*Bu$_2$Al)P$_7$] 13
2.2. Synthesis of [K(18c6)]$_2$[κ^2-(*i*Bu$_2$Al)As$_7$] 19
2.3. Attempted Synthesis of [Na(18c6)]$_2$[κ^2-((Me$_2$N)$_2$Ga)P$_7$] 22
3. Catalytic Hydroboration of Carbon Dioxide 23
3.1. Comparison of Catalysts 23
3.2. Isotopic Labelled Studies 25
3.2.1. Hydroboration of ^{13}CO$_2$ using [Na(18c6)]$_2$[κ^2-(*i*Bu$_2$Al)P$_7$] 25
3.2.2. Hydroboration of ^{13}CO$_2$ using [K(18c6)]$_2$[κ^2-(*i*Bu$_2$Al)As$_7$] 26
3.3. Detailed Studies Performance [Na(18c6)]$_2$[(*i*Bu$_2$Al)P$_7$] 27
3.3.1. General Procedure Hydroboration CO$_2$ using [Na(18c6)]$_2$[2] 27
3.3.2. Investigating Product Selectivity using [Na(18c6)]$_2$[2] 28
3.3.3. Thermal Decomposition of [Na(18c6)]$_2$[2] 30
3.4. [Na(18c6)]$_2$[(*i*Bu$_2$Al)P$_7$] Catalyst Recycling 33
3.5. [Na(18c6)]$_2$[(*i*Bu$_2$Al)P$_7$] Catalyst Recovery 35
3.6. Comparison to Literature Metal-free Catalysts for CO$_2$ Hydroboration 37
3.7. Comparison to Literature Metal Catalysts for CO$_2$ Hydroboration 38
4. Experimental Mechanistic studies 40

4.1. Stoichiometric Reactions 40
4.1.1. Addition of HBin to [Na(18c6)]$_2$[2] 40
4.1.2. Addition of HBBN to [Na(18c6)]$_2$[2] 41
4.1.3. Addition of Benzaldehyde to [Na(18c6)]$_2$[2] 42
4.1.4. Probing Equilibrium Mono- and Bis-insertion of Benzaldehyde into [2]$^{2-}$ 55
4.1.5. Addition of Acetophenone to [Na(18c6)]$_2$[2] 56
4.1.6. Addition of Benzaldehyde to [K(18c6)]$_2$[3] 58
4.1.7. Addition of Acetophenone to [K(18c6)]$_2$[3] 59
4.1.8. Addition of Benzaldehyde to [Na(18c6)]$_2$[4] 60
4.1.9. Addition of Acetophenone to [Na(18c6)]$_2$[4] 61
4.2. Stoichiometric Hydroboration of Benzaldehyde Using [Na(18c6)]$_2$[1] 62
4.2.1. First Benzaldehyde Addition Followed by HBpin 62
4.2.2. First HBpin Addition Followed by Benzaldehyde 63
4.3. Stoichiometric Hydroboration of Benzaldehyde Using [Na(18c6)]$_2$[2] 65
4.3.1. First Benzaldehyde Addition Followed by HBpin 65
4.3.2. First HBpin Addition Followed by Benzaldehyde 67
4.4. Stoichiometric Hydroboration of Benzaldehyde Using [K(18c6)]$_2$[3] 68
4.4.1. First Benzaldehyde Addition Followed by HBpin 68
4.4.2. First HBpin Addition Followed by Benzaldehyde 69
4.5. Stoichiometric Hydroboration of Benzaldehyde Using [K(18c6)]$_2$[3] 70
4.5.1. First Benzaldehyde Addition Followed by HBpin 70
4.5.2. First HBpin addition followed by benzaldehyde 71
5. Variable Time Normalization Analysis 73
5.1. Order in Reagents of the Hydroboration of Benzaldehyde Using HBBN dimer 74
5.2. Order in Reagents of the Hydroboration of Benzaldehyde Using HBpin 93
6. Kinetic Isotope Effect 112
7. Crystallography Table 114
8. Details of computational study 116
9. References 119

1. Methods and Materials

1.1. Experimental Considerations

All manipulations were performed under an inert atmosphere using standard Schlenk-line, and glovebox (MBraun Unilab) techniques. Glassware was flame dried prior to use.

Dry DMF, THF, diethyl ether, toluene, and pentane were obtained using Innovative Technologies anhydrous engineering solvent purification systems and subsequently degassed. DME, pyridine, and hexane were dried over Na or K, purified by distillation. *o*DFB was dried over CaH_2 and purified by distillation. THF-d_8, C_6D_6, were dried over activated 3 Å molecular sieves. All solvents were stored over activated 3 Å molecular sieves.

Red phosphorus, arsenic powder, naphthalene, pinacol borane, HBBN dimer, 18-crown-6, benzaldehyde, acetophenone were purchased from a commercial source (Apollo Scientific, Sigma-Aldrich, Alfa Aesar, Fuorochem, Tokyo Chemical Industry, Thermo Fisher Scientific, and Acros Organics) and used without purification. Elemental sodium and elemental potassium were cleaned by removal of the oxide layers and washing with toluene and hexane. Carbon dioxide (cp grade, 99.95%) was purchased from BOC Ltd. Clusters $[Na(DME)_x]_3P_7$,[1] K_3P_7,[1] $[K(DME)_x]_3As_7$,[2] $(Me_3Si)_3P_7$,[1] $[Na(18c6)]_2[HP_7]$,[3, 4] $[K(18c6)]_2[HP_7]$,[3, 4] $[K(18c6)]_2[HAs_7]$,[3, 4] $[Na(18c6)]_2[(BBN)P_7]$,[5] $[K(18c6)]_2[(BBN)P_7]$,[5] $[Na(18c6)]_2[(Ph_2In)P_7]$,[6] and $[K(18c6)]_2[(Ph_2In)P_7]$[6] were synthesized using (modified) literature procedures, see section 1.4. below.

1.2. Analytical Considerations

NMR Spectroscopy. ^{1}H, ^{11}B, $^{11}B\{^{1}H\}$, $^{13}C\{^{1}H\}$, ^{31}P NMR and ^{31}P COSY spectra were recorded on a Bruker AVIII 400 spectrometer (operating frequencies: 399.78 MHz, 128.36 MHz, 100.53 MHz and 161.83 MHz for ^{1}H, ^{11}B, ^{13}C, and ^{31}P, respectively) or a Bruker AVIII HD 600 spectrometer (operating frequencies: 600.42 MHz, and 150.99 MHz for ^{1}H, and ^{13}C respectively). ^{1}H and $^{13}C\{^{1}H\}$ NMR chemical shifts were internally

referenced to the residual solvent resonances (C_6D_6 (benzene-d_6): ^1H δ = 7.16 ppm, $^{13}C\{^1H\}$ δ = 128.02 ppm, THF-d_8 (tetrahydrofuran-d_8): ^1H δ = 3.58, 1.73 ppm, $^{13}C\{^1H\}$ δ = 67.57, 25.37 ppm. ^{11}B, ^{31}P chemical shifts were externally referenced to $BF_3 \cdot Et_2O$, H_3PO_4, respectively. Solution phase NMR samples were prepared under an inert atmosphere in 5 mm J Young NMR tubes. 1D and COSY NMR data was analyzed using MestReNova V14.0.0 software or Topspin V3.6.1 software.

Elemental Analysis. Elemental analysis was carried out by the microanalysis service of the University of Manchester using a Flash 2000 elemental analyser. Samples were prepared under a nitrogen atmosphere.

Mass spectrometry. Mass spectrometry samples were measured by the mass spectrometry service of the University of Manchester using an electrospray ionization (ESI) or atmospheric pressure chemical ionization (APCI) equipped Thermo Orbitrap Executive Plus Extended Mass Range mass spectrometer. Samples were prepared under a nitrogen atmosphere and directly injected into the ionization source of the mass spectrometer.

Infrared spectroscopy. Infrared (IR) spectra were recorded on a Bruker Alpha II spectrometer, an attenuated total reflectance FT-IR spectrometer (4000-500 cm^{-1}, 32 scans.

1.3. X-ray Diffraction Studies

Data collection: X-ray diffraction data for $[Na(18c6)]_2$[**2**], and $[K(18c6)]_2$[**3**] were collected on a dual wavelength Rigaku FR-X rotating anode diffractometer using CuKα (λ = 1.54184 Å) and MoKα (0.71073Å) radiation, respectively, equipped with an AFC-11 4-circle kappa geometry goniometer, VariMAX™ microfocus optics, a Hypix-6000HE detector and an Oxford Cryosystems Cryostream 800 nitrogen flow gas system, at a temperature of 100 K. Data were collected and reduced using Rigaku CrysAlisPro v42.[7] Absorption correction was performed using empirical methods (SCALE3 ABSPACK) based upon symmetry-equivalent reflections combined with measurements at different azimuthal angles.

Crystal structure determination and refinements: The crystal structure was solved and refined against all F^2 values using the SHELX and Olex2 suite of programmes.[8, 9] All non-hydrogen atoms were refined anisotropically. Hydrogen atoms were placed in calculated positions and refined using idealized geometries. Hydrogen isotropic atomic displacement parameters were constrained to ride with the parent atom with an appropriate multiplier for the hybridisation. For $[K(18c6)]_2$[**3**], similar neighbour atomic displacement parameter and enhanced rigid bond restraints were applied globally to the model in order to refine chemically sensible atomic displacement parameters given the poor quality of the data. While there is clearly disorder of 18c6 and isobutyl group C13, C14, C15 and C16, this could not be modelled on account of the low resolution of the data and the poor data-to-parameter ratio that would have resulted. Stronger atomic displacement parameter restraints were applied to these groups.

Crystallographic data for $[Na(18c6)]_2$[**2**], and $[K(18c6)]_2$[**3**] have been deposited with the CCDC (CCDC 2365860 and 2365861). These data can be obtained free of charge via https://www.ccdc.cam.ac.uk/structures/ (or from the Cambridge Crystallographic Data Centre, 12 Union Road, Cambridge CB21EZ, UK; Tel: (+44)1223-336-408; or deposit@ccdc.cam.ac.uk).

1.4. Synthesis Literature Compounds

1.4.1. Synthesis of $[Na(DME)_x]_3P_7$

Following literature procedure,[1] a Schlenk flask with a stir bar was charged with red phosphorus (25.16 g, 0.808 mol, 7 eq.), sodium (8.00 g, 0.344 mmol, 3 eq.) and naphthalene (2.936 g, 23 mmol, 0.1 eq.). The mixture was cooled to 0 °C and suspended in THF (35 mL) and DME (35 mL). The reaction mixture was stirred overnight to give a dark suspension. The headspace of the flask was removed under vacuum and the reaction mixture was slowly heated to 55 °C over the course of a day and left to stir over 3 days until a green/yellow suspension was observed. The suspension was cooled to room temperature and was washed with pentane (3 x 40

mL). The product was then dried under vacuum over the course of a day, yielding a green/yellow powder. The $[Na(DME)_x]_3P_7$ was used without further purification.

Isolated Yield: 34.02 g, 98%.

^{31}P NMR (162 MHz, 298 K, DMF): δ = –113.02 (broad singlet) ppm.

1.4.2. Synthesis of K_3P_7

Following literature procedure,[1] a Schlenk flask with a stir bar was charged with red phosphorus (3.70 g, 51 mmol, 7 eq.), sodium (2.00 g, 51 mmol, 3 eq.) and naphthalene (218 mg, 1.7 mmol, 0.1 eq.). The mixture was cooled to 0 °C and suspended in THF (20 mL) and DME (20 mL). The reaction mixture was stirred overnight to give a dark suspension. The headspace of the flask was removed under vacuum and the reaction mixture was slowly heated to 55 °C over the course of a day and left to stir over 3 days until a green/yellow suspension was observed. The suspension was cooled to room temperature and was washed with pentane (3 x 40 mL). The product was then dried under vacuum over the course of a day, yielding a green powder. The K_3P_7 was used without further purification.

Isolated Yield: 5.51 g, 96%.

^{31}P NMR (162 MHz, 298 K, DMF): δ = –112.09 (broad singlet) ppm.

1.4.3. Synthesis of $[K(DME)_x]_3As_7$

Following literature procedure,[2] a Schlenk flask with a stir bar was charged with grey arsenic (2.25 g, 30 mmol, 7 eq.), potassium (0.500 g, 12.5 mmol, 3 eq.) and naphthalene (55 mg, 0.42 mmol, 0.1 eq.). The mixture was cooled to 0 °C and suspended in THF (8 mL) and DME (8 mL). The reaction mixture was stirred overnight to give a dark suspension. The headspace of the flask was removed under vacuum and the reaction mixture was slowly heated to 55 °C over the course of a day and left to stir over 7 days until a red suspension was observed. The suspension was cooled to room temperature and was washed with pentane (3 x 20 mL). The product was then

dried under vacuum over the course of 3 days, yielding a red powder. The $[K(DME)x]_3As_7$ was used without further purification.

Isolated Yield: 2.53 g, 92%.

1.4.4. Synthesis of $(Me_3Si)_3P_7$

Following literature procedure,[1] a Schlenk flask with a stir bar was charged with $[Na(DME)_x]_3P_7$ (3.00 g, 10.09 mmol, 1 eq.) and the solids were suspended in toluene (10 mL). While the suspension was stirring, Me_3SiCl (3.97 mL, 31.28 mmol, 3.1 eq.) was added dropwise after which the reaction mixture was allowed to react overnight. The mixture was filtered and the black residue was further extracted with toluene (10 mL) combining both filtrates yielding a clear yellow solution. Removal of volatiles under reduced pressure yielded yellow solids.

Isolated Yield: 3.93 g, 89%.

^{1}H NMR (400 MHz, 298 K, Tol-d_8): δ = 0.05 (s) ppm.

^{31}P NMR (162 MHz, 298 K, Tol-d_8): δ = 4.24 – –5.12 (m, 3P, bridging P), –95.21 – –104.41 (m, 1P, apical P), –152.79 – –161.20 (m, 3P, basal P) ppm.

1.4.5. Synthesis of $[Na(18c6)]_2[HP_7]$

Following literature procedure,[3,4] a Schlenk flask with a stir bar was charged with $[Na(DME)_x]_3P_7$ (500 mg, 1.68 mmol, 1 eq.) and the solids were suspended in pyridine (1 mL). While the suspension was stirring at 0 °C, H_2O (60.7 µL, 3.36 mmol, 2 eq.) was added dropwise after which the reaction mixture was allowed for 5 min. To the reaction mixture 18c6 (888 mg, 3.36 mmol, 2 eq.) was added under a flow of N_2 and the reaction mixture was allowed to react for 15 min. The mixture was filtered yielding a clear dark red solution. While stirring, the solution was concentrated under reduced pressure to *ca.* 0.5 mL after which toluene (10 mL) was added and an orange solid formed. The product was then dried under vacuum over the course of 7 day, yielding an orange powder.

Isolated Yield: 1.06 g, 80%.

^{1}H NMR (400 MHz, 298 K, DMF): δ = 3.57 (s, 48H, 18c6), 0.39 (broad singlet, 1H, HP_7) ppm.

^{31}P NMR (162 MHz, 298 K, DMF): δ = –24.57 (broad singlet, 1P), –111.01 (broad singlet, 6P) ppm.

1.4.6. Synthesis of $[K(18c6)]_2[HP_7]$

Following literature procedure,[3,4] a Schlenk flask with a stir bar was charged with K_3P_7 (561 mg, 1.68 mmol, 1 eq.) and the solids were suspended in pyridine (1 mL). While the suspension was stirring at 0 °C, H_2O (60.7 µL, 3.36 mmol, 2 eq.) was added dropwise after which the reaction mixture was allowed for 5 min. To the reaction mixture 18c6 (888 mg, 3.36 mmol, 2 eq.) was added under a flow of N_2 and the reaction mixture was allowed to react for 15 min. The mixture was filtered yielding a clear dark red solution. While stirring, the solution was concentrated under reduced pressure to *ca.* 0.5 mL after which toluene (10 mL) was added and an orange solid formed. The product was then dried under vacuum over the course of 7 day, yielding an orange powder.

Isolated Yield: 1.18 g, 86%.

^{1}H NMR (400 MHz, 298 K, DMF): δ = 3.56 (s, 48H, 18c6), 0.46 (broad singlet, 1H, HP_7) ppm.

^{31}P NMR (162 MHz, 298 K, DMF): δ = –21.26 (broad singlet, 1P), –111.82 (broad singlet, 6P) ppm.

1.4.7. Synthesis of $[K(18c6)]_2[HAs_7]$

Following literature procedure,[3,4] a Schlenk flask with a stir bar was charged with $[K(DME)_x]_3As_7$ (1078 mg, 1.68 mmol, 1 eq.) and the solids were suspended in pyridine (1 mL). While the suspension was stirring at 0 °C, H_2O (60.7 µL, 3.36 mmol, 2 eq.)

was added dropwise after which the reaction mixture was allowed for 5 min. To the reaction mixture 18c6 (888 mg, 3.36 mmol, 2 eq.) was added under a flow of N_2 and the reaction mixture was allowed to react for 15 min. The mixture was filtered yielding a clear dark red solution. While stirring, the solution was concentrated under reduced pressure to *ca.* 0.5 mL after which toluene (10 mL) was added and a black solid formed. The product was then dried under vacuum over the course of 7 day, yielding a black powder.

Isolated Yield: 569 mg, 30%.

1.4.8. Synthesis of $[Na(18c6)]_2[(BBN)P_7]$

Following literature procedure,[5] a Schlenk flask with a stir bar was charged with $[Na(18c6)]_2[HP_7]$ (2.01 g, 2.54 mmol, 1.0 eq.), HBBN dimer (928 mg, 3.80 mmol, 1.5 eq.). THF (40 mL) was added to the mixture and gas evolution was observed. The reaction mixture was allowed to stir until no gas evolution was observed. The mixture was filtered yielding a clear dark orange solution. The volatiles were removed under reduced pressure and the residue was washed with toluene (5 x 20mL). The residue was dissolved in THF (5 mL) and filtered yielding a clear dark orange solution. The solution was filtered and removal of volatiles under reduced pressure yielded glassy orange solids.

Isolated Yield: 1.42 g, 61%.

^{1}H NMR (400 MHz, 298 K, THF-d_8): δ = 3.41 (s, 48H, 18c6), 2.31 – 1.65 (m, 14H, BBN) ppm.

^{31}P NMR (162 MHz, 298 K, THF-d_8): δ = 76.45 (ddd, $^{1}J_{PP}$ = 345.1, 212.9, $^{2}J_{PP}$ = 60.5 Hz, 2P, basal), 45.75 (dtd, $^{1}J_{PP}$ = 376.1, 234.2, $^{2}J_{PP}$ = 56.8 Hz, 1P, apical), –31.76 (dd (pseudo triplet), $^{1}J_{PP}$ = 368.6 Hz, 1P, bridging), –56.52 – –63.33 (m, 1P, basal), –153.15 – –159.99 (m, 2P, bridging) ppm.

Elemental analysis for $C_{32}H_{62}BNa_2O_{12}P_7$: calcd.: C 42.12, H 6.85, N 0.0; found C 42.25, H 7.09, N 0.0.

1.4.9. Synthesis of $[K(18c6)]_2[(BBN)P_7]$

Following literature procedure,[5] a Schlenk flask with a stir bar was charged with $[K(18c6)]_2[HP_7]$ (2.09 g, 2.54 mmol, 1.0 eq.), HBBN dimer (928 mg, 3.80 mmol, 1.5 eq.). THF (40 mL) was added to the mixture and gas evolution was observed. The reaction mixture was allowed to stir until no gas evolution was observed. The mixture was filtered yielding a clear dark orange solution. The volatiles were removed under reduced pressure and the residue was washed with toluene (5 x 20mL). The residue was dissolved in THF (5 mL) and filtered yielding a clear dark orange solution. The solution was filtered and removal of volatiles under reduced pressure yielded glassy orange solids.
Isolated Yield: 1.35 g, 57%.

^{1}H NMR (400 MHz, 298 K, THF-d_8): δ = 3.48 (s, 48H, 18c6), 2.23 – 1.60 (m, 14H, BBN) ppm.

^{31}P NMR (162 MHz, 298 K, THF-d_8): δ = 76.43 (ddd, $^1J_{PP}$ = 343.9, 214.2, $^2J_{PP}$ = 61.2 Hz, 2P, basal), 45.89 (dtd, $^1J_{PP}$ = 375.2, 234.6, $^2J_{PP}$ = 57.2 Hz, 1P, apical), –31.76 (dd (pseudo triplet), $^1J_{PP}$ = 369.1 Hz, 1P, bridging), –57.23 – –63.51 (m, 1P, basal), –153.14 – –159.84 (m, 2P, bridging) ppm.

Elemental analysis for $C_{32}H_{62}BK_2O_{12}P_7$: calcd.: C 40.69, H 6.62, N 0.0; found C 40.23, H 6.95, N 0.0.

1.4.10. Synthesis of $[Na(18c6)]_2[(Ph_2In)P_7]$

Following literature procedure,[6] a Schlenk flask with a stir bar was charged with $[Na(DME)_x]_3P_7$ (89.1 mg, 0.30 mmol, 1 eq.), 18c6 (158.5 mg, 0.60 mmol, 2 eq.) and $InPh_3$ (103.8 mg, 0.3 mmol, 1 eq.). The solids were suspended in pyridine (3 mL) and allowed to react for 2 days. The reaction mixture was filtered yielding a dark red filtrate. To this filtrate toluene was added (10 mL) yielding a brown precipitate. The solids were dried under reduced pressure over the course of 2 days, yielding a brown powder.
Isolated Yield: 92.3 mg, 29%.

^{31}P NMR (162 MHz, 298 K, DMF): δ = 6.06 – –0.01 (m, 2P), –47.56 – –55.17 (m, 1P), –67.50 (dd, $^{1}J_{PP}$ = 403.1, 331.0 Hz, 1P), –77.23 (dtd, $^{1}J_{PP}$ = 406.8, 206.0, $^{2}J_{PP}$ = 53.1 Hz, 1P), –166.33 – –174.46 (m, 2P) ppm.

Elemental analysis for $C_{36}H_{58}InNa_2O_{12}P_7$: calcd.: C 40.77, H 5.51, N 0.0; found C 41.12, H 5.31, N 0.0.

1.4.11. Synthesis of [K(18c6)]$_2$[(Ph$_2$In)P$_7$]

Following literature procedure,[6] a Schlenk flask with a stir bar was charged with K_3P_7 (100.1 mg, 0.30 mmol, 1 eq.), 18c6 (158.5 mg, 0.60 mmol, 2 eq.) and $InPh_3$ (103.8 mg, 0.3 mmol, 1 eq.). The solids were suspended in pyridine (3 mL) and allowed to react for 2 days. The reaction mixture was filtered yielding a dark red filtrate. To this filtrate toluene was added (10 mL) yielding a brown precipitate. The solids were dried under reduced pressure over the course of 2 days, yielding a green/brown powder.
Isolated Yield: 72 mg, 22%.

^{31}P NMR (162 MHz, 298 K, DMF): δ = 5.95 – –0.12 (m, 2P), –46.21 – –55.34 (m, 1P), –67.69 (dd, $^{1}J_{PP}$ = 403.9, 330.2 Hz, 1P), –76.84 (dtd, $^{1}J_{PP}$ = 407.2, 204.9, $^{2}J_{PP}$ = 52.4 Hz, 1P), –165.98 – –173.53 (m, 2P) ppm.

Elemental analysis for $C_{36}H_{58}InK_2O_{12}P_7$: calcd.: C 39.57, H 5.35, N 0.0; found C 39.75, H 5.12, N 0.0.

2. Synthesis and Characterization of catalysts

2.1. Synthesis of $[Na(18c6)]_2[\kappa^2\text{-}(\textit{i}Bu_2Al)P_7]$

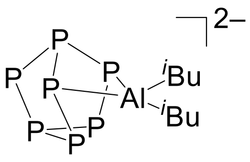

2 $[Na(18c6)]^+$

To a Schlenk flask charged with a stir bar and $[Na(18c6)]_2[HP_7]$ (250 mg, 0.32 mmol, 1 eq.) THF (5 mL) was added and cooled to –30 °C forming a slurry. A solution of diisopropyl aluminium hydride (45 mg, 0.32 mmol. 1 eq.) in THF (5 mL) was cooled to –30 °C and dropwise added to the $[Na(18c6)]_2[HP_7]$ slurry. Gas evolution was observed, and the reaction was allowed to react for 5 min at –30 °C, after which it was warmed to RT. The mixture was filtered yielding a clear dark orange solution. The solvent was removed under reduced pressure and the residue was washed with toluene (2 x 20 mL). The residue was dissolved in THF and filtered again yielding a dark orange solution. Removal of volatiles under reduced pressure yielded glassy orange solids. Crystals suitable for X-ray diffraction analysis were obtained through slow diffusion of hexane into a concentrated THF solution.

Isolated Yield: 187 mg, 63%.

^{1}H NMR (400 MHz, 298 K, THF-d_8): δ = 3.58 (s, 48H, *18-crown-6*, overlap with THF-d_8 residual resonance), 2.29 (th, $^3J_{HH}$ = 13.1, 6.6 Hz, 1H, $AlCH_2C\textit{H}(CH_3)_2$), 1.48 (th, $^3J_{HH}$ = 13.3, 6.6 Hz, 1H, $AlCH_2C\textit{H}(CH_3)_2$), 1.01 (d, $^3J_{HH}$ = 6.5 Hz, 6H, $AlCH_2CH(C\textit{H}_3)_2$), 0.61 (d, $^3J_{HH}$ = 6.5 Hz, 6H, $AlCH_2CH(C\textit{H}_3)_2$), 0.11 (d, $^3J_{HH}$ = 6.9 Hz, 2H, $AlC\textit{H}_2CH(CH_3)_2$), –0.89 (d, $^3J_{HH}$ = 7.0 Hz, 2H, $AlC\textit{H}_2CH(CH_3)_2$) ppm.

^{13}C{^{1}H} NMR (101 MHz, 298 K, THF-d_8): δ = 68.09 (s, *18-crown-6*), 26.91 (s, $Al^i\textit{Bu}_2$), 26.86 (s, $Al^i\textit{Bu}_2$), 26.50 (s, $Al^i\textit{Bu}_2$), 26.11 (s, $Al^i\textit{Bu}_2$), 25.84 (s, $Al^i\textit{Bu}_2$), 25.72 (s, $Al^i\textit{Bu}_2$) ppm.

^{31}P NMR (162 MHz, 298 K, THF-d_8): δ = –29.15 – –36.99 (m, 2P, *bridging*), –45.26 (dd, $^1J_{PP}$ = 399.6, 342.8 Hz, 1P, *bridging*), –52.58 – –55.58 (m, 1P, *basal*), -55.58 – –64.20 (m, 1P, *bridging*), –171.20 – –182.04 (m, 2P, *basal*) ppm. Full assignment of the ^{31}P NMR spectrum below in Figure S6.

Elemental analysis for $C_{32}H_{66}AlNa_2O_{12}P_7$: calcd.: C 41.21, H 7.13, N 0.0; found C 41.27, H 7.12, N 0.0

Note: The cluster was not observed by mass spectrometry, possibly due to its high air and mositure sensitivity resulting in cluster decomposition.

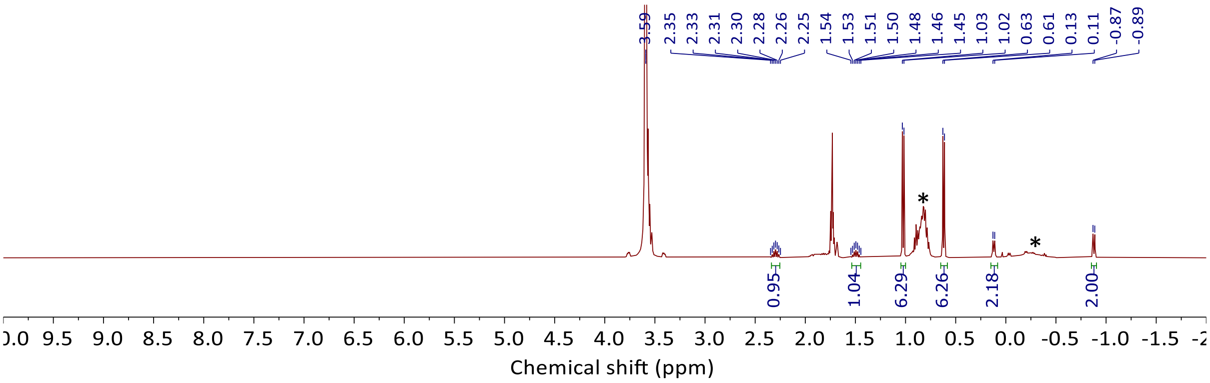

Figure S1. ^{1}H NMR spectrum (400 MHz, THF-d_8) of [Na(18c6)]$_2$[**2**]. Resonances marked by * are possibly from small amount of [Na(18c6)]$_2$[κ^1-**2**], similar behaviour was observed for [Na(18c6)]$_2$[**1**].[5]

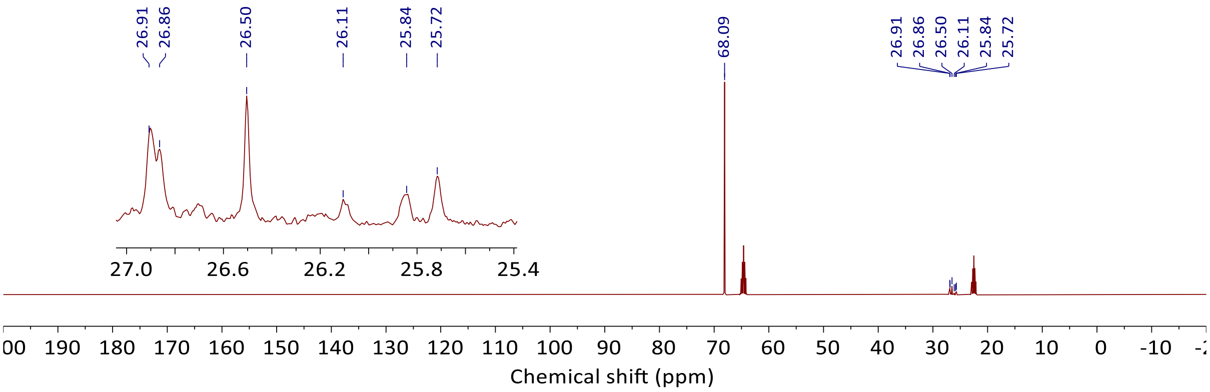

Figure S2. ^{13}C{^{1}H} NMR spectrum (101 MHz, THF-d_8) of [Na(18c6)]$_2$[**2**].

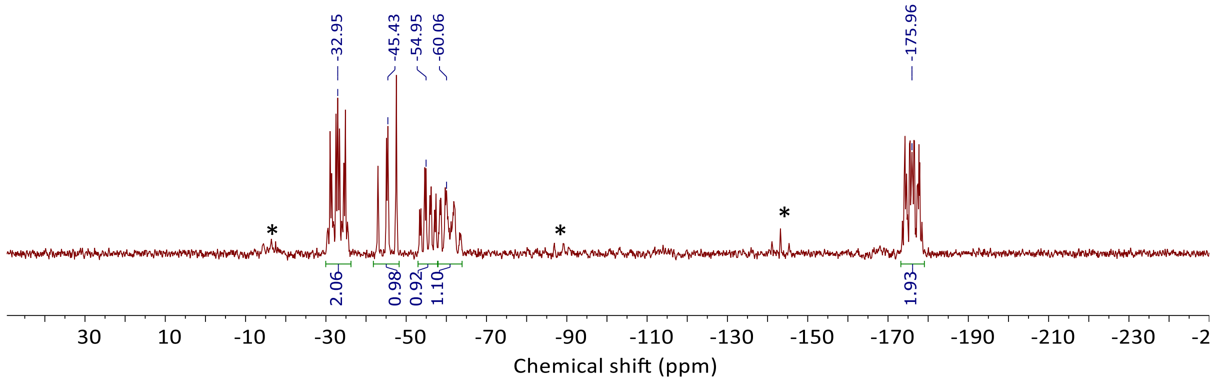

Figure S3. ^{31}P NMR spectrum (162 MHz, THF-d_8) of $[Na(18c6)]_2$[**2**]. Resonances marked by * are possibly from small amount of $[Na(18c6)]_2[\kappa^1$-**2**], similar behaviour was observed for $[Na(18c6)]_2$[**1**].[5]

Variable temperature (VT) NMR spectroscopy was employed to investigate the fluxional κ^1- and κ^2-coordination modes. ^1H VT NMR spectroscopy studies resulted in inconclusive results, see Figure S4. Where the ^{31}P VT NMR spectroscopy studies (Figure S5) revealed a number of resonances (labelled by *) appearing. Around –30 °C $[Na(18c6)]_2$[**2**] oiled out of the solution inhibiting measurement of the sample and small features and coupling at lower temperatures are difficult to identify.

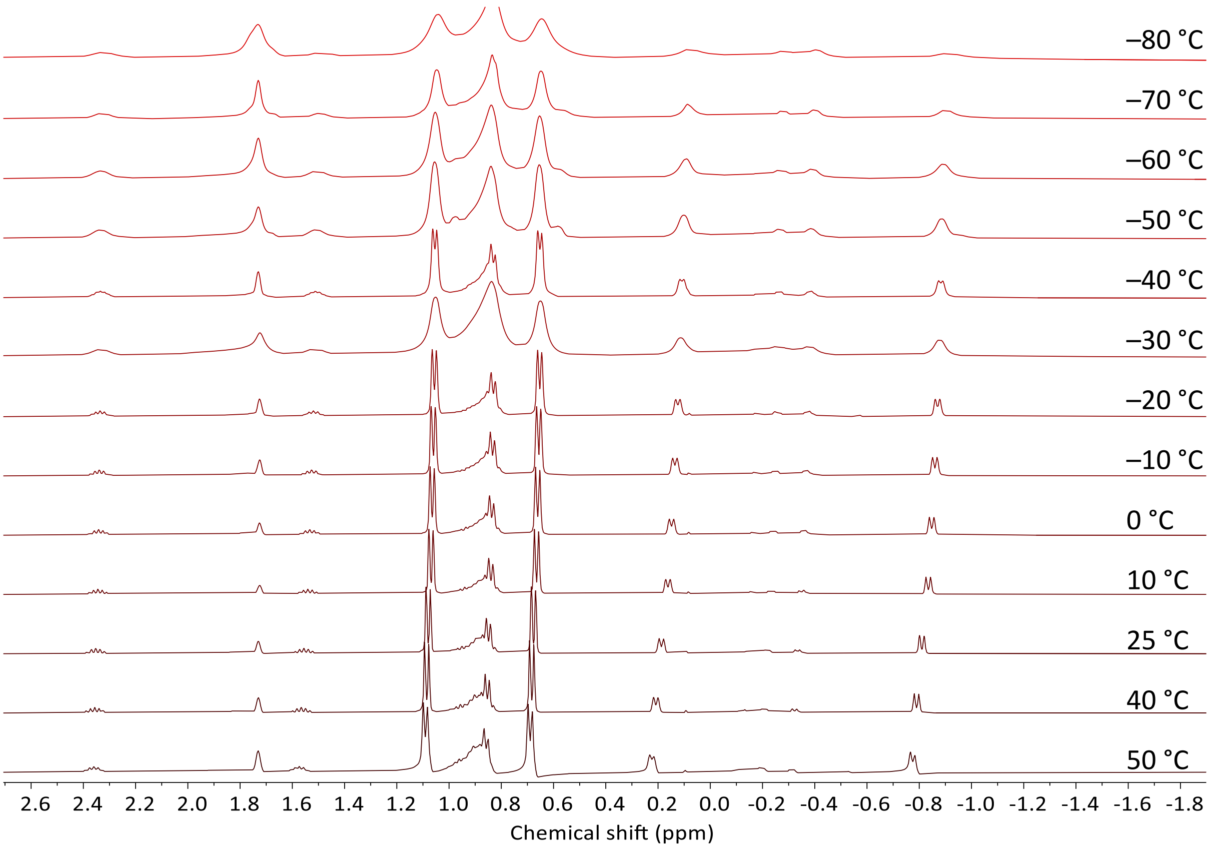

Figure S4. ^{1}H VT NMR spectra (400 MHz, THF-d_8) of $[Na(18c6)]_2$[**2**]. Note: around –30 °C $[Na(18c6)]_2$[**2**] oiled out of the solution inhibiting accurate shimming of the sample.

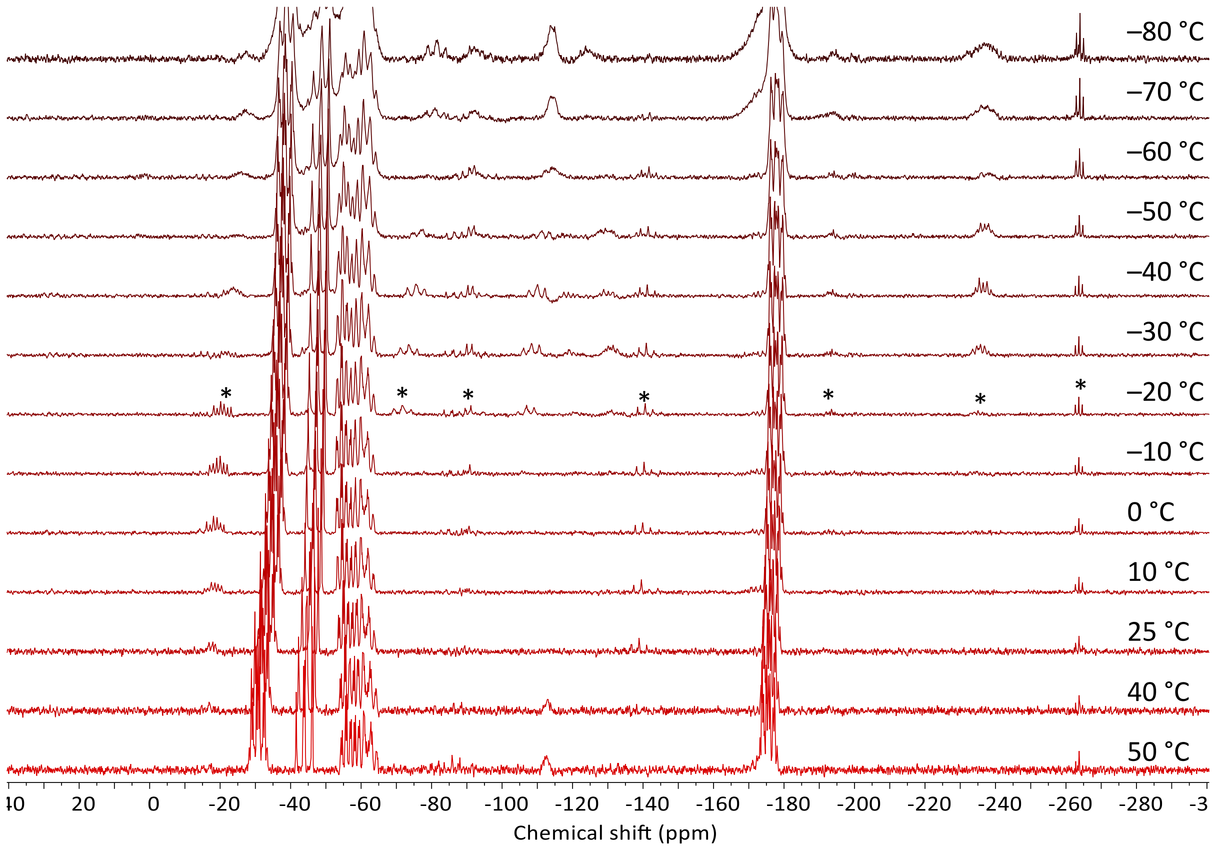

Figure S5. ^{31}P VT NMR spectra (162 MHz, THF-d_8) of $[Na(18c6)]_2$[**2**]. Note: around –30 °C $[Na(18c6)]_2$[**2**] oiled out of the solution inhibiting accurate shimming of the sample.

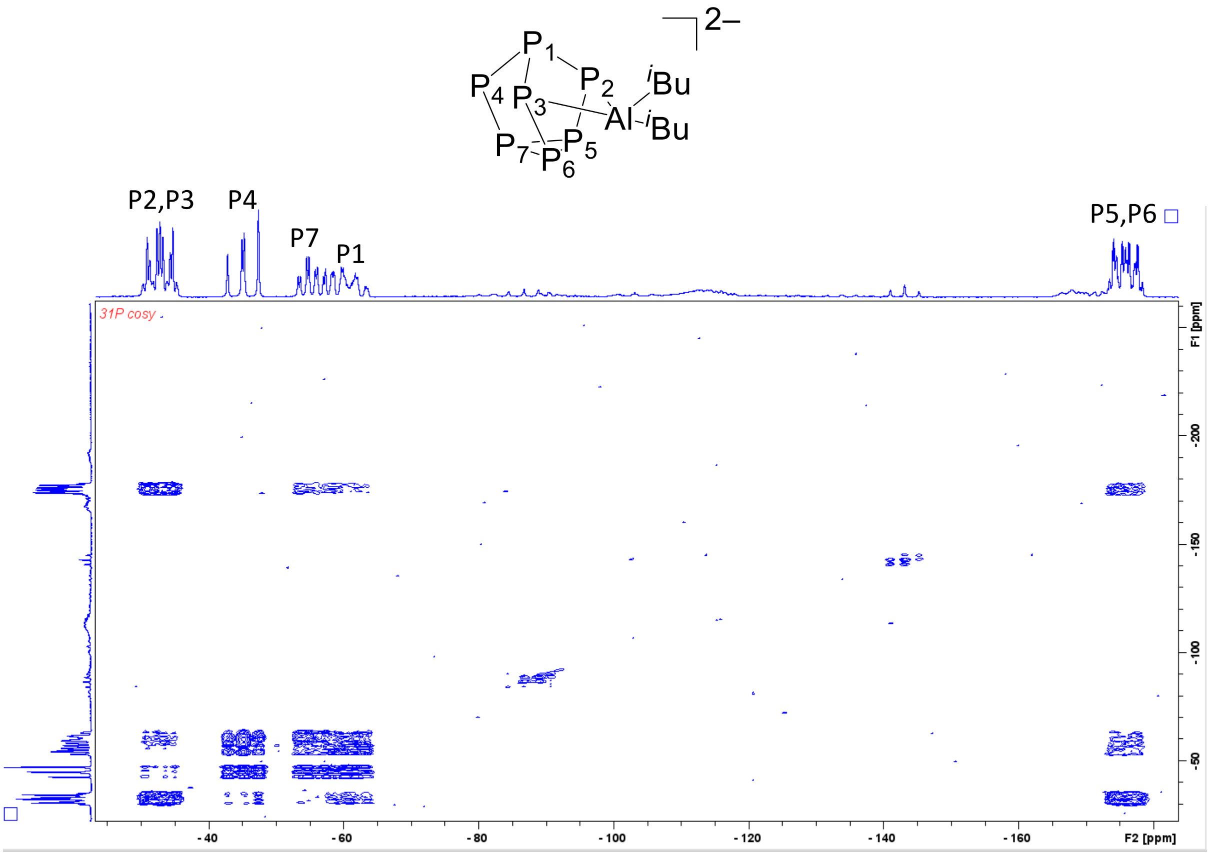

Figure S6. ^{31}P COSY NMR spectrum (162 MHz, THF-d_8) of [Na(18c6)]$_2$[**2**].

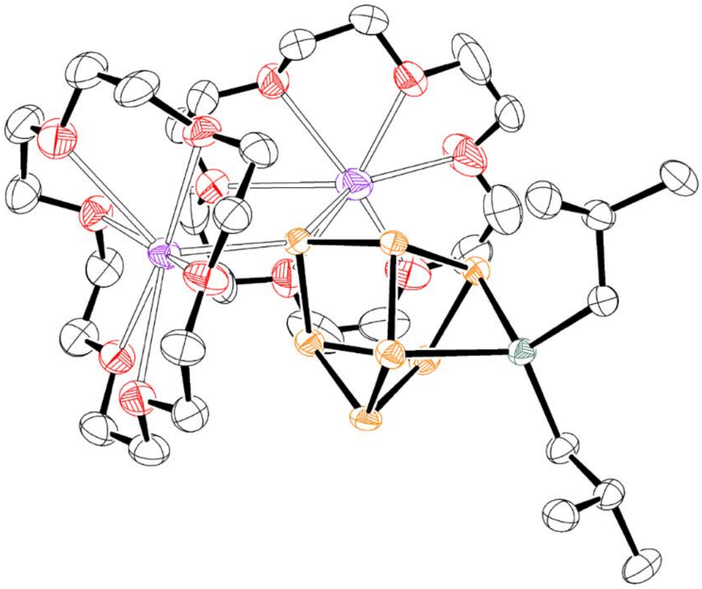

Figure S7. Molecular structure of [Na(18c6)]$_2$[**2**]. Anisotropic displacement ellipsoids pictured at 50% probability. Hydrogen atoms omitted for clarity. Phosphorus: Orange; Aluminium: Green; Carbon: White; Sodium: Purple; Oxygen: Red. CCDC code: 2365860.

2.2. Synthesis of $[K(18c6)]_2[\kappa^2\text{-}(iBu_2Al)As_7]$

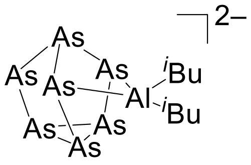

2 $[K(18c6)]^+$

To a Schlenk flask charged with a stir bar and $[K(18c6)]_2[HAs_7]$ (250 mg, 0.22 mmol. 1 eq.) THF (5 mL) was added and cooled to –30 °C forming a slurry. A solution of diisopropyl aluminium hydride (31 mg, 0.22 mmol. 1 eq.) in THF (5 mL) was cooled to –30 °C and dropwise added to the $[K(18c6)]_2[HAs_7]$ slurry. Gas evolution was observed and the reaction was allowed to react for 5 min at –30 °C, after which it was warmed to RT. The mixture was filtered yielding a clear black solution. The solvent was removed under reduced pressure and the residue was washed with toluene (2 x 20 mL). The residue was dissolved in THF and filtered again yielding a black solution. Removal of volatiles under reduced pressure yielded dark brown solids. Crystals suitable for X-ray diffraction analysis were obtained through slow diffusion of hexane into a concentrated *o*DFB solution. In solution the cluster was found to decompose slowly on exposure to light, forming presumably elemental As.

Isolated Yield: 101 mg, 71%.

^{1}H NMR (400 MHz, 298 K, THF-d_8): δ = 3.61 (s, 48H, *18-crown-6*, overlap with THF-d_8 residual resonance), 2.26 (th, $^3J_{HH}$ = 13.2, 6.6 Hz, 1H, $AlCH_2C\mathit{H}(CH_3)_2$), 1.55 (th, $^3J_{HH}$ = 13.3, 6.7 Hz, 1H, $AlCH_2C\mathit{H}(CH_3)_2$), 1.03 (d, $^3J_{HH}$ = 6.5 Hz, 6H, $AlCH_2CH(C\mathit{H_3})_2$), 0.66 (d, $^3J_{HH}$ = 6.5 Hz, 6H, $AlCH_2CH(C\mathit{H_3})_2$), 0.25 (d, $^3J_{HH}$ = 6.9 Hz, 2H, $AlC\mathit{H_2}CH(CH_3)_2$), –0.71 (d, $^3J_{HH}$ = 7.0 Hz, 2H, $AlC\mathit{H_2}CH(CH_3)_2$) ppm.

^{13}C{^{1}H} NMR (101 MHz, 298 K, THF-d_8): δ = 68.39 (s, *18-crown-6*), 29.69 (s, $Al^i\mathit{Bu_2}$), 28.35 (s, $Al^i\mathit{Bu_2}$), 26.76 (s, $Al^i\mathit{Bu_2}$), 26.38 (s, $Al^i\mathit{Bu_2}$), 25.96 (s, $Al^i\mathit{Bu_2}$), 25.82 (s, $Al^i\mathit{Bu_2}$) ppm.

Elemental analysis for $C_{32}H_{66}AlK_2O_{12}As_7$: calcd.: C 30.20, H 5.32, N 0.0; found C 30.61, H 5.09, N 0.0

Note: The cluster was not observed by mass spectrometry, possibly due to its high air and mositure sensitivity resulting in cluster decomposition.

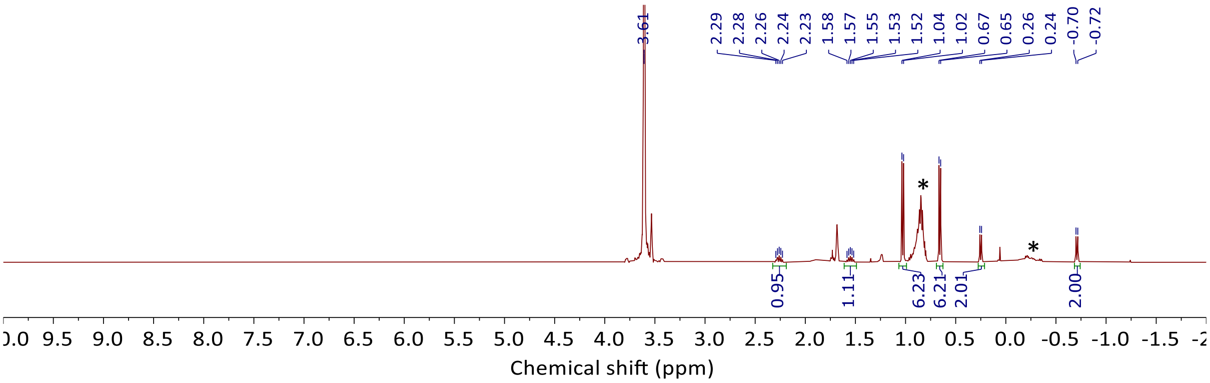

Figure S8. ^{1}H NMR spectrum (400 MHz, THF-d_8) of [K(18c6)]$_2$[**3**]. Resonances marked by * are possibly from small amount of [K(18c6)]$_2$[κ^1-**3**], similar behaviour was observed for [Na(18c6)]$_2$[**1**].

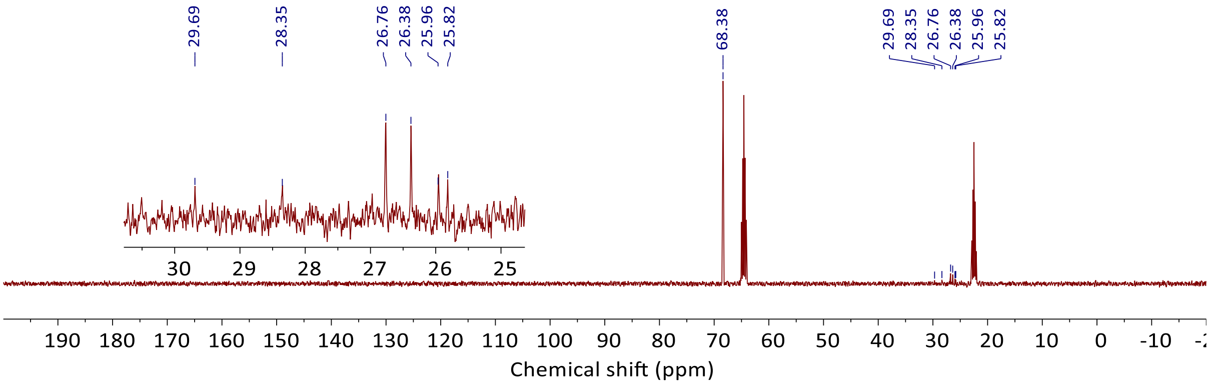

Figure S9. ^{13}C{^{1}H} NMR spectrum (101 MHz, THF-d_8) of [K(18c6)]$_2$[**3**].

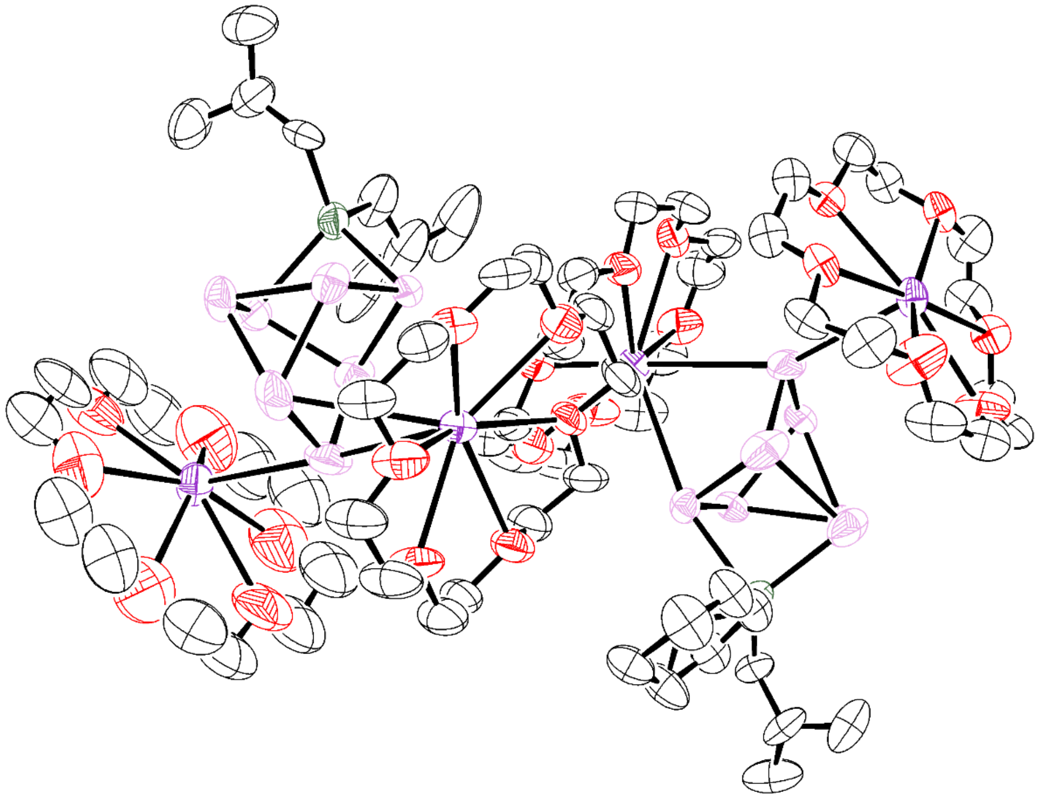

Figure S10. Molecular structure of [K(18c6)]$_2$[**3**]. Anisotropic displacement ellipsoids pictured at 50% probability. Hydrogen atoms omitted for clarity. Arsenic: Plum; Aluminium: Green; Carbon: White; Potassium: Purple; Oxygen: Red. CCDC code: 2365861.

2.3. Attempted Synthesis of $[Na(18c6)]_2[\kappa^2\text{-}((Me_2N)_2Ga)P_7]$

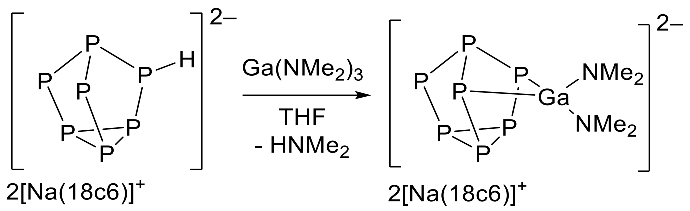

In a glovebox a sample vial was charged with $[Na(18c6)]_2[HP_7]$ (50 mg, 0.064 mmol, 1 eq.) and $Ga(NMe_2)_3$ dimer (13 mg, 0.032 mmol, 0.5 eq.). THF (0.5 mL, held at –30 °C) was added and the reaction mixture was allowed to react at –30 °C for 30 min. Gas evolution was observed. The reaction mixture was allowed to warm to RT. The reaction was filtered and investigated by ^{31}P NMR spectroscopy. The solvent was removed under reduced pressure and the residue was washed with toluene (2 x 20 mL). The residue was dissolved in THF and filtered again yielding a black solution. Removal of volatiles under reduced pressure yielded dark oil. Despite multiple efforts to wash the oil (wash with hexane, pentane or ether), clean isolation was not achieved.

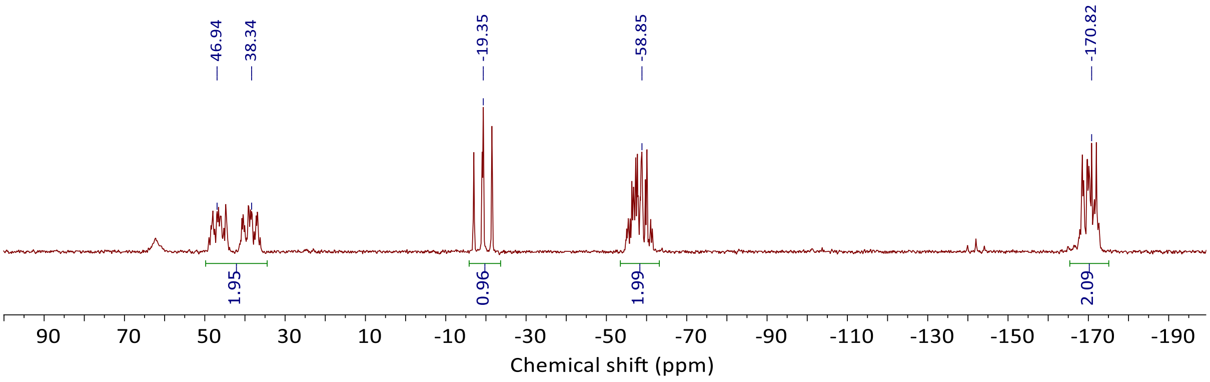

Figure S11. ^{31}P NMR spectrum (162 MHz, Reaction mixture) of attempted synthesis $[Na(18c6)]_2[((Me_2N)_2Ga)P_7]$.

3. Catalytic Hydroboration of Carbon Dioxide

3.1. Comparison of Catalysts

To a J Young NMR tube C_6Me_6, HBBN dimer (36 mg, 0.15 mmol), and a solution of catalysts (with mol % relative to HBBN monomer) in *o*DFB:toluene (0.6 mL, 1:1) was added. The reaction mixture was degassed and the headspace was refilled with CO_2 (1 atm). The reaction was monitored by ^{1}H, ^{11}B and ^{11}B{^{1}H} NMR spectroscopy. The NMR conv. was calculated by integration of the crude ^{1}H NMR spectrum using the C_6Me_6 as an internal standard (^{1}H δ = 2.20 ppm). Where possible reaction profiles were recorded to determine an accurate end time. A representative set of example NMR spectra and reaction profiles are given below.

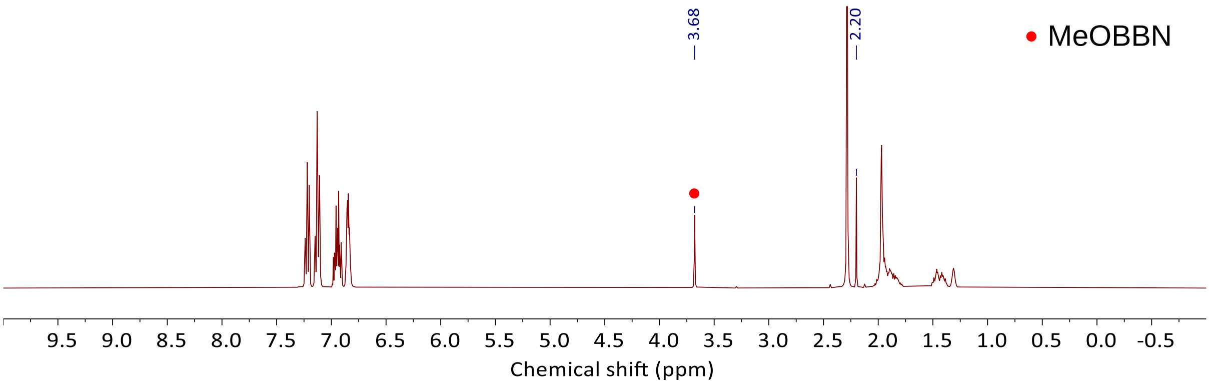

Figure S12. ^{1}H NMR spectrum (400 MHz, *o*DFB:toluene) of the hydroboration of CO_2 by [Na(18c6)]$_2$[**2**] (0.1 mol% at RT).

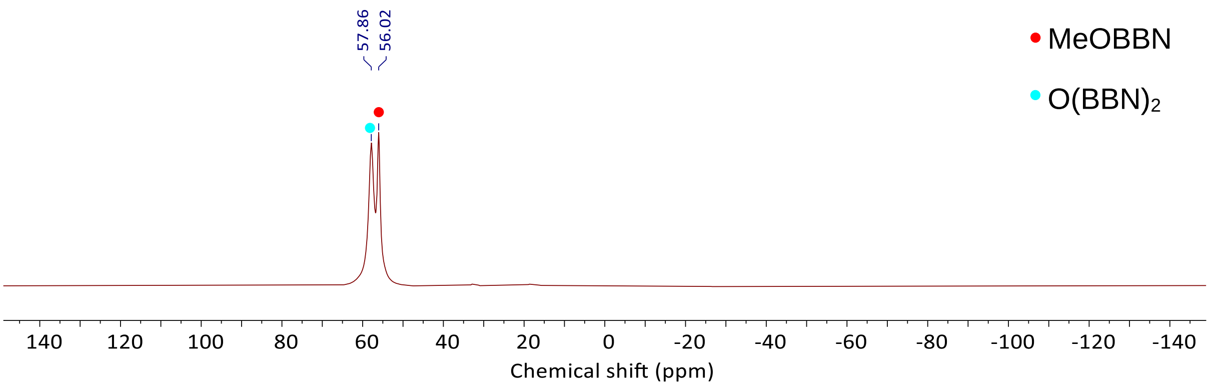

Figure S13. ^{11}B NMR spectrum (128 MHz, *o*DFB:toluene) of the hydroboration of CO_2 by [Na(18c6)]$_2$[**2**] (0.1 mol% at RT).

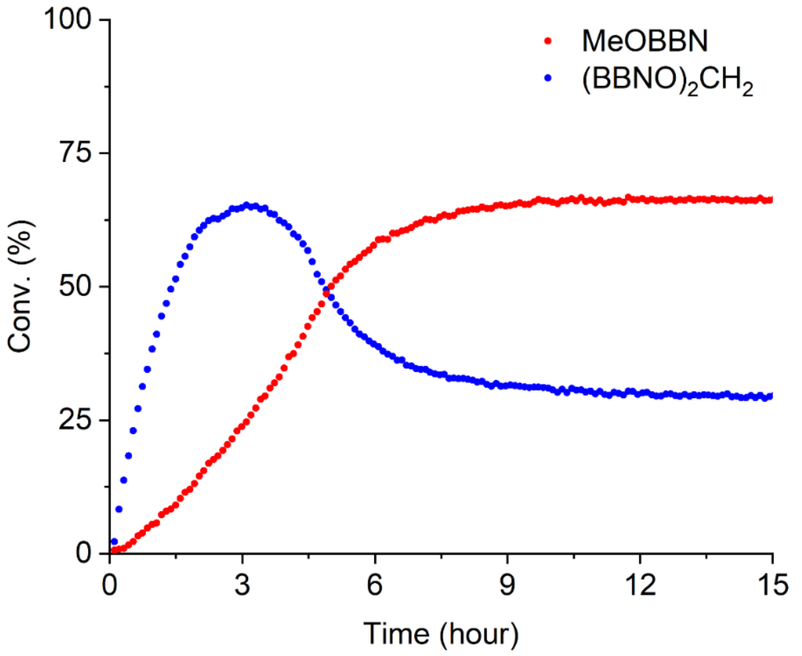

Figure S14. Tracked conversion of the hydroboration of CO_2 using [Na(18c6)]$_2$[**2**] (0.33 mol%) at RT.

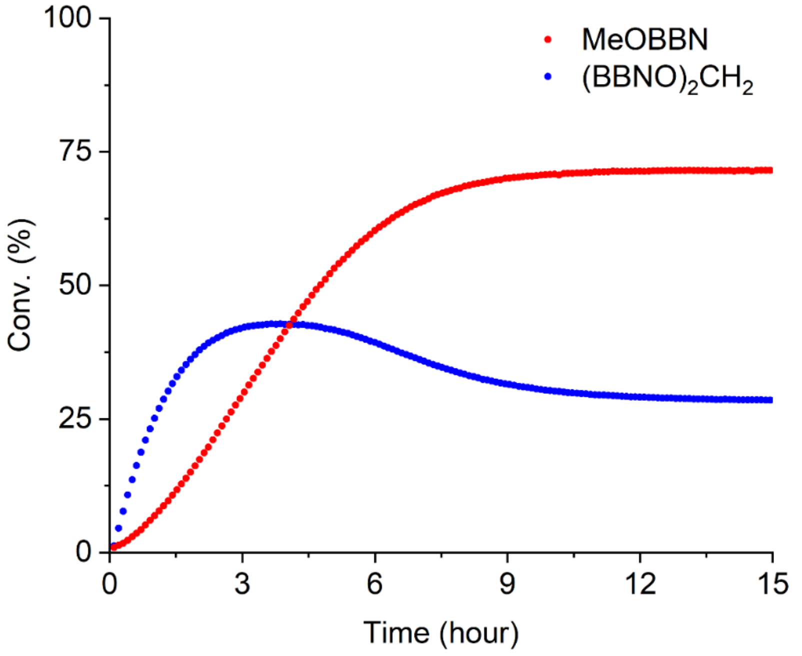

Figure S15. Tracked conversion of the hydroboration of CO_2 using [K(18c6)]$_2$[**3**] (0.33 mol%) at RT.

3.2. Isotopic Labelled Studies

3.2.1. Hydroboration of $^{13}CO_2$ using [Na(18c6)]$_2$[κ^2-(*i*Bu$_2$Al)P$_7$]

To a J Young NMR tube C_6Me_6, HBBN dimer (36 mg, 0.15 mmol), and a solution of [Na(18c6)][**2**] (0.9 mg, 0.9 µmol, 0.0033 eq.) in *o*DFB:toluene (0.6 mL, 1:1) was added. The reaction mixture was degassed and the headspace was refilled with $^{13}CO_2$ (1 atm). The reaction was monitored by ^{1}H, ^{11}B, ^{11}B{^{1}H} and ^{13}C{^{1}H} NMR spectroscopy.

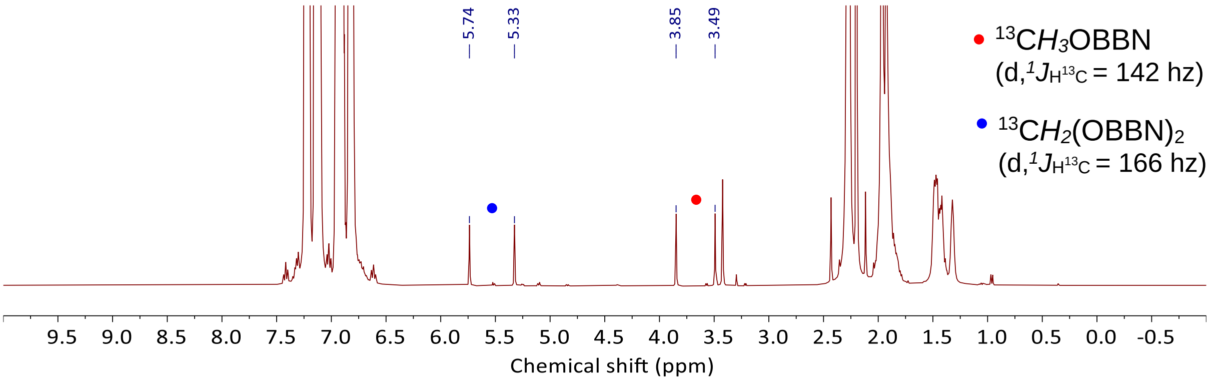

Figure S16. ^{1}H NMR spectrum (400 MHz, *o*DFB:toluene) of the hydroboration of $^{13}CO_2$ by [Na(18c6)]$_2$[**2**] (0.33 mol% at RT).

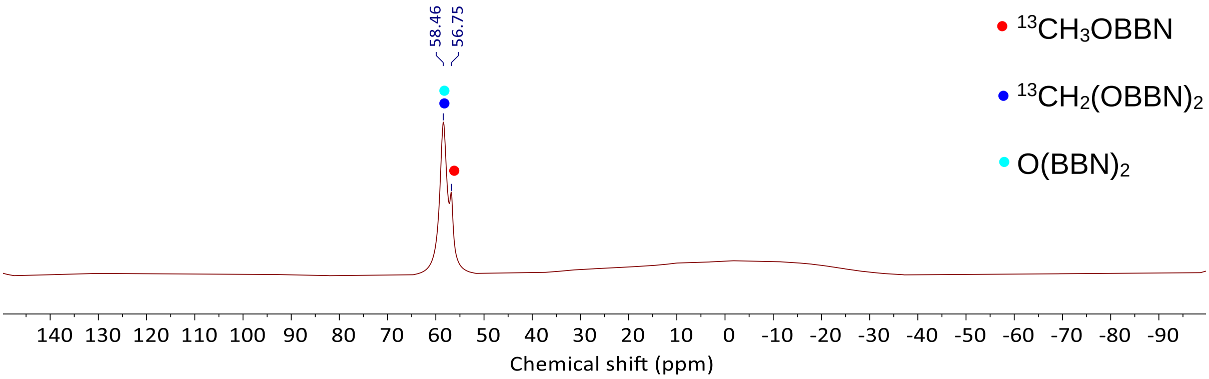

Figure S17. ^{11}B NMR spectrum (128 MHz, *o*DFB:toluene) of the hydroboration of $^{13}CO_2$ by [Na(18c6)]$_2$[**2**] (0.33 mol% at RT).

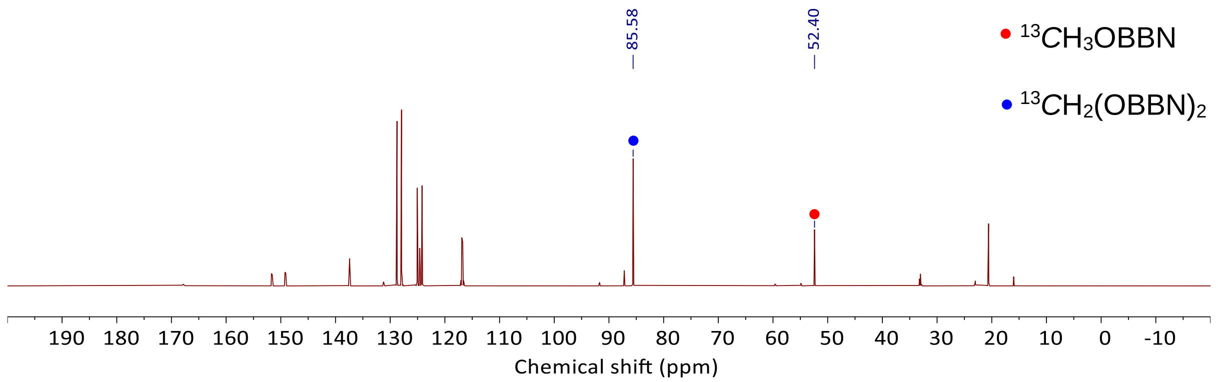

Figure S18. $^{13}C\{^1H\}$ NMR spectrum (101 MHz, *o*DFB:toluene) of the hydroboration of $^{13}CO_2$ by [Na(18c6)]$_2$[**2**] (0.33 mol% at RT).

3.2.2. Hydroboration of $^{13}CO_2$ using [K(18c6)]$_2$[κ^2-(*i*Bu$_2$Al)As$_7$]

To a J Young NMR tube C_6Me_6, HBBN dimer (36 mg, 0.15 mmol), and a solution of [K(18c6)][**3**] (1.3 mg, 0.9 µmol, 0.0033 eq.) in *o*DFB:toluene (0.6 mL, 1:1) was added. The reaction mixture was degassed and the headspace was refilled with $^{13}CO_2$ (1 atm). The reaction was monitored by ^1H, ^{11}B, $^{11}B\{^1H\}$ and $^{13}C\{^1H\}$ NMR spectroscopy.

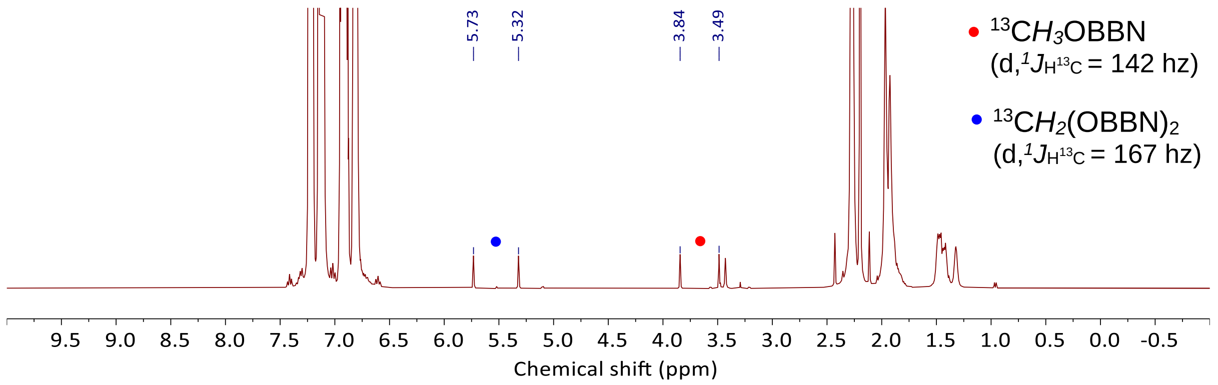

Figure S19. ^1H NMR spectrum (400 MHz, *o*DFB:toluene) of the hydroboration of $^{13}CO_2$ by [K(18c6)]$_2$[**3**] (0.33 mol% at RT).

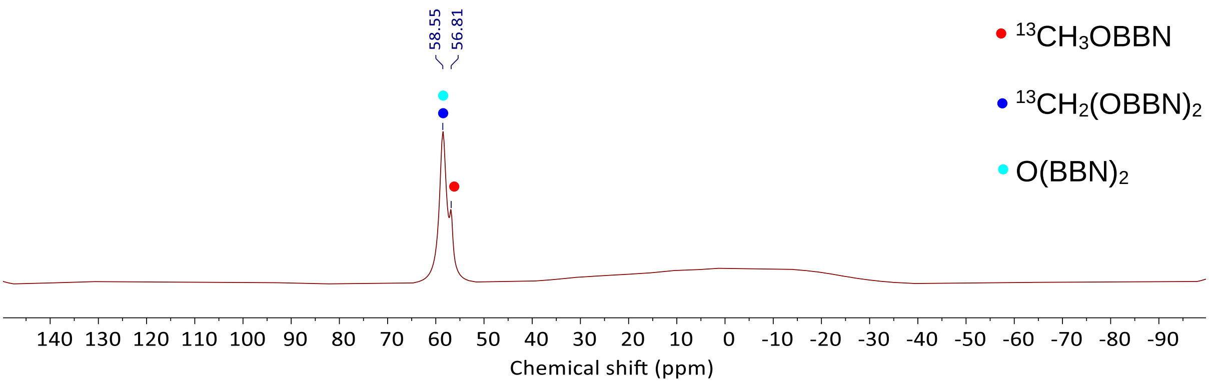

Figure S20. ^{11}B NMR spectrum (128 MHz, *o*DFB:toluene) of the hydroboration of $^{13}CO_2$ by [K(18c6)]$_2$[**3**] (0.33 mol% at RT).

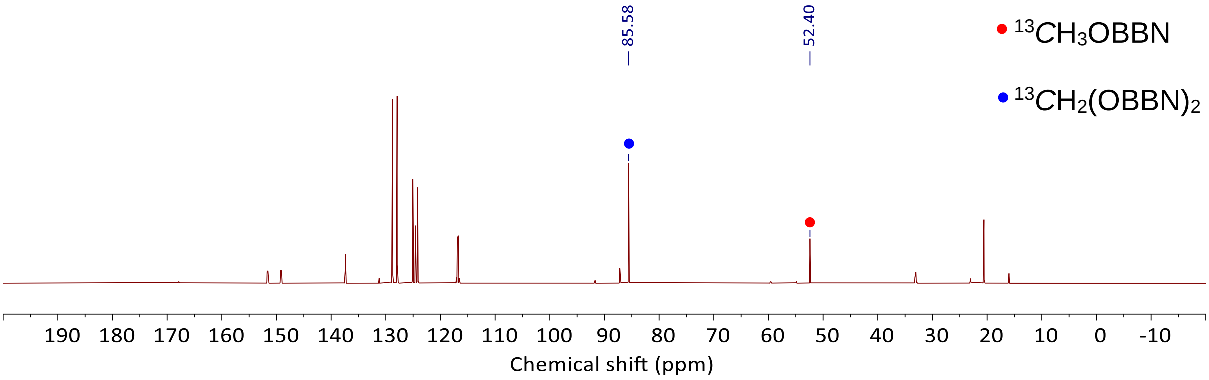

Figure S21. $^{13}C\{^1H\}$ NMR spectrum (101 MHz, *o*DFB:toluene) of the hydroboration of $^{13}CO_2$ by [K(18c6)]$_2$[**3**] (0.33 mol% at RT).

3.3. Detailed Studies Performance [Na(18c6)]$_2$[(*i*Bu$_2$Al)P$_7$]

3.3.1. General Procedure Hydroboration CO_2 using [Na(18c6)]$_2$[2]

To a J Young NMR tube C_6Me_6, HBBN dimer (36 mg, 0.15 mmol), and a solution of [Na(18c6)][**2**] (with mol % relative to HBBN monomer) in *o*DFB:toluene (0.6 mL, 1:1) was added. The reaction mixture was degassed and the headspace was refilled with CO_2 (1 atm). The reaction was monitored by ^1H, ^{11}B and $^{11}B\{^1H\}$ NMR spectroscopy. The NMR conv. was calculated by integration of the crude ^1H NMR spectrum using the C_6Me_6 as an internal standard (^1H δ = 2.20 ppm). Where possible reaction profiles were recorded to determine an accurate end time.

3.3.2. Investigating Product Selectivity using [Na(18c6)]$_2$[2]

Dilution experiment:

To a J Young NMR tube C_6Me_6 (9.8 mg, 0.06 mmol), HBBN dimer (18 mg, 0.075 mmol, 0.5 eq.), and a solution of [Na(18c6)][**2**] (0.45 mg, 0.45 µmol, 0.0033 eq.) in *o*DFB:toluene (0.6 mL, 1:1) was added. The reaction mixture was degassed and the headspace was refilled with CO_2 (1 atm). The reaction was monitored by ^{1}H, ^{11}B and ^{11}B{^{1}H} NMR spectroscopy. The NMR conv. was calculated by integration of the crude ^{1}H NMR spectrum using the C_6Me_6 as an internal standard (^{1}H δ = 2.20 ppm) and the product distribution found was 25:75 $CH_2(OBBN)_2$:MeOBBN, overall conv. >99%.

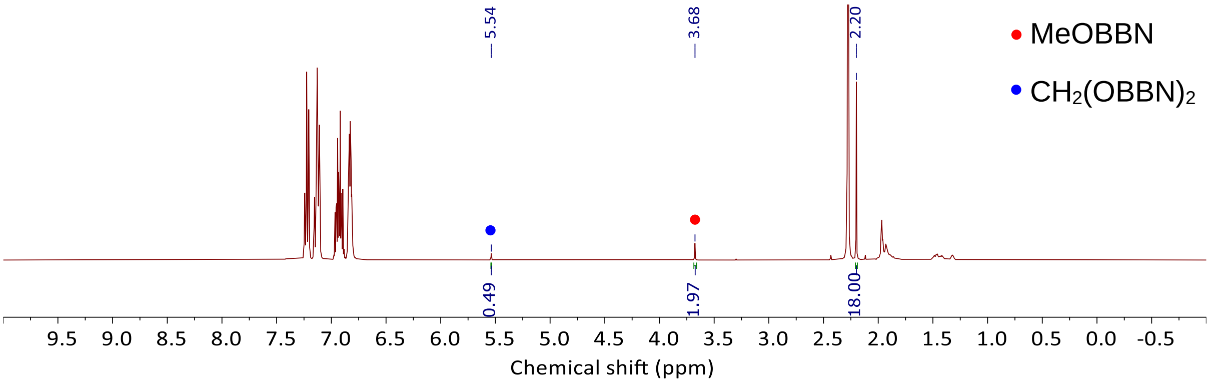

Figure S22. ^{1}H NMR spectrum (400 MHz, *o*DFB:toluene) of CO_2 hydroboration using [Na(18c6)]$_2$[**2**] at 0.97mM catalyst concentration.

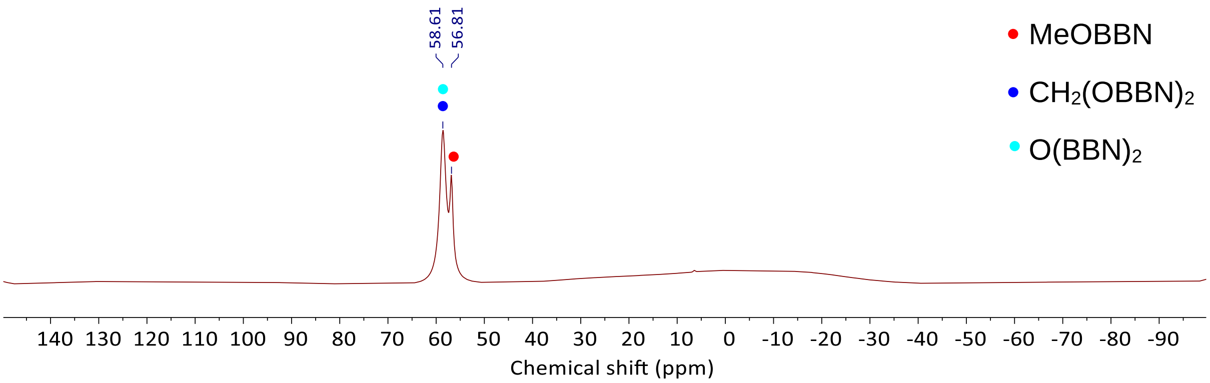

Figure S23. ^{11}B NMR spectrum (128 MHz, *o*DFB:toluene) of CO_2 hydroboration using [Na(18c6)]$_2$[**2**] at 0.97mM catalyst concentration.

Surface area experiment:

To a J Young ampoule (see schematic on the right) C_6Me_6 (16.2 mg, 0.10 mmol), HBBN dimer (36 mg, 0.15 mmol, 0.5 eq.), and a solution of [Na(18c6)][**2**] (0.9 mg, 0.9 µmol, 0.0033 eq.) in *o*DFB:toluene (0.6 mL, 1:1) was added. The reaction mixture was degassed and the headspace was refilled with CO_2 (1 atm). After 3 days the reaction vessel was opened under Ar and the reaction mixture was transferred into a J Young NMR tube in the glovebox. The reaction was monitored by ^1H, ^{11}B and $^{11}B\{^1H\}$ NMR spectroscopy. The NMR conv. was calculated by integration of the crude ^1H NMR spectrum using the C_6Me_6 as an internal standard (^1H δ = 2.20 ppm) and the product distribution found was 63:37 $CH_2(OBBN)_2$:MeOBBN, overall conv. 86%.

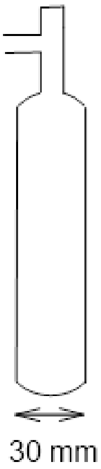

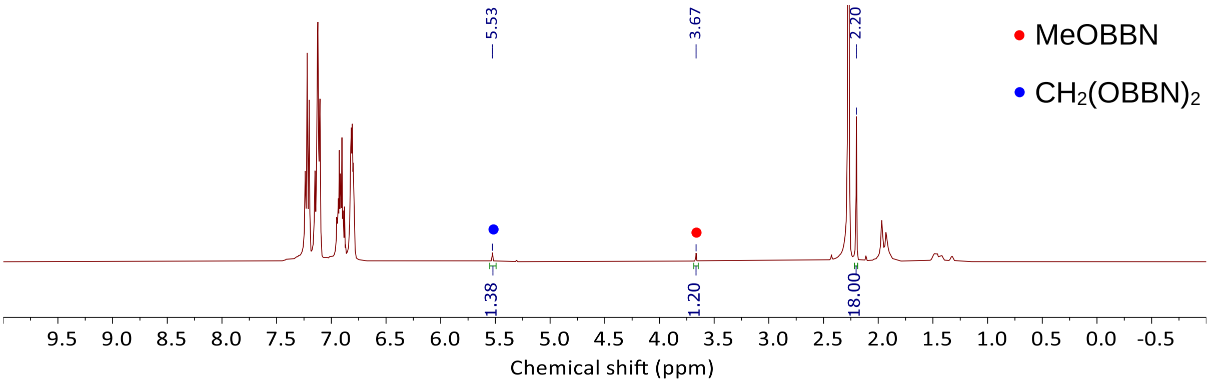

Figure S24. ^1H NMR spectrum (400 MHz, *o*DFB:toluene) of CO_2 hydroboration using $[Na(18c6)]_2$[**2**] in a J Young ampoule.

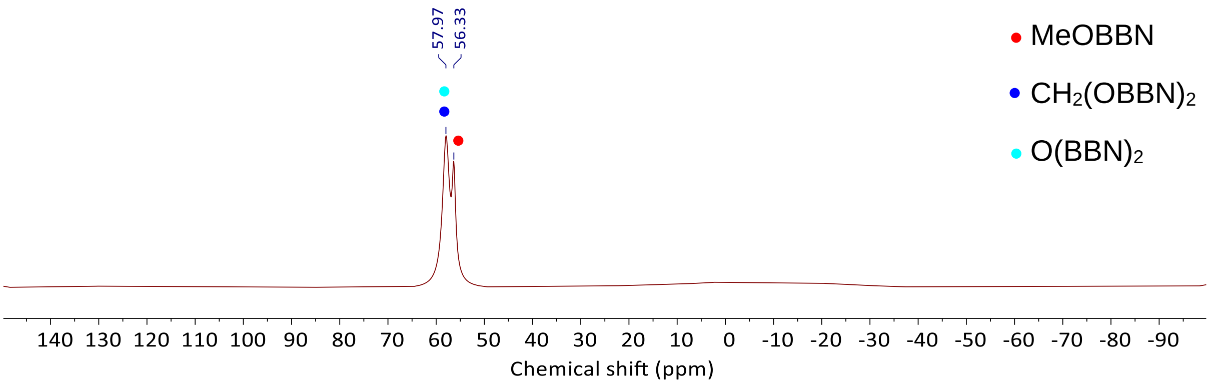

Figure S25. ^{11}B NMR spectrum (128 MHz, *o*DFB:toluene) of CO_2 hydroboration using $[Na(18c6)]_2$[**2**] in a J Young ampoule.

3.3.3. Thermal Decomposition of [Na(18c6)]$_2$[2]

To three separate J Young NMR tubes a solution of [Na(18c6)][**2**] (25 mg) in *o*DFB (0.6 mL) was added. The reaction mixture was heated to 50 °C, 60 °C or 70 °C. The reaction was monitored by ^{31}P NMR spectroscopy.

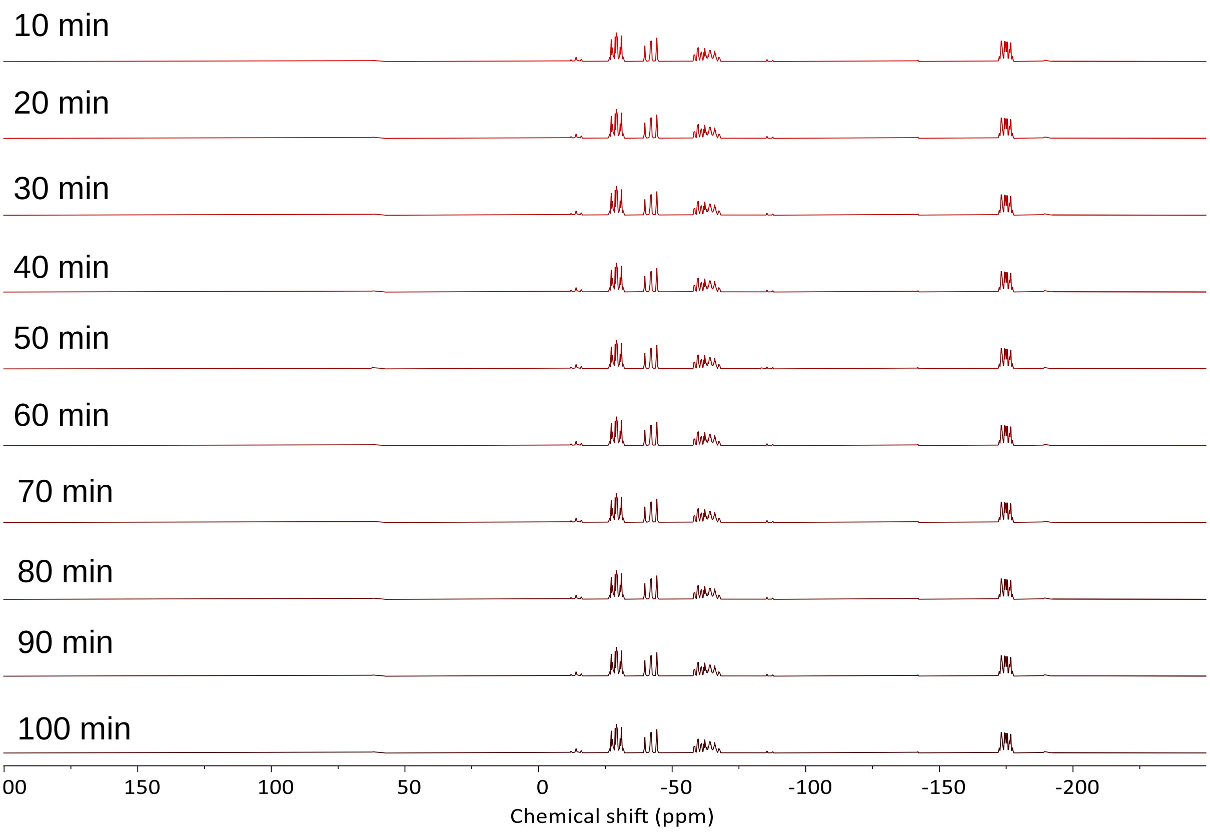

Figure S26. Tracked ^{31}P NMR spectrum (162 MHz, *o*DFB) of [Na(18c6)]$_2$[**2**] heated at 50 °C. No decomposition observed.

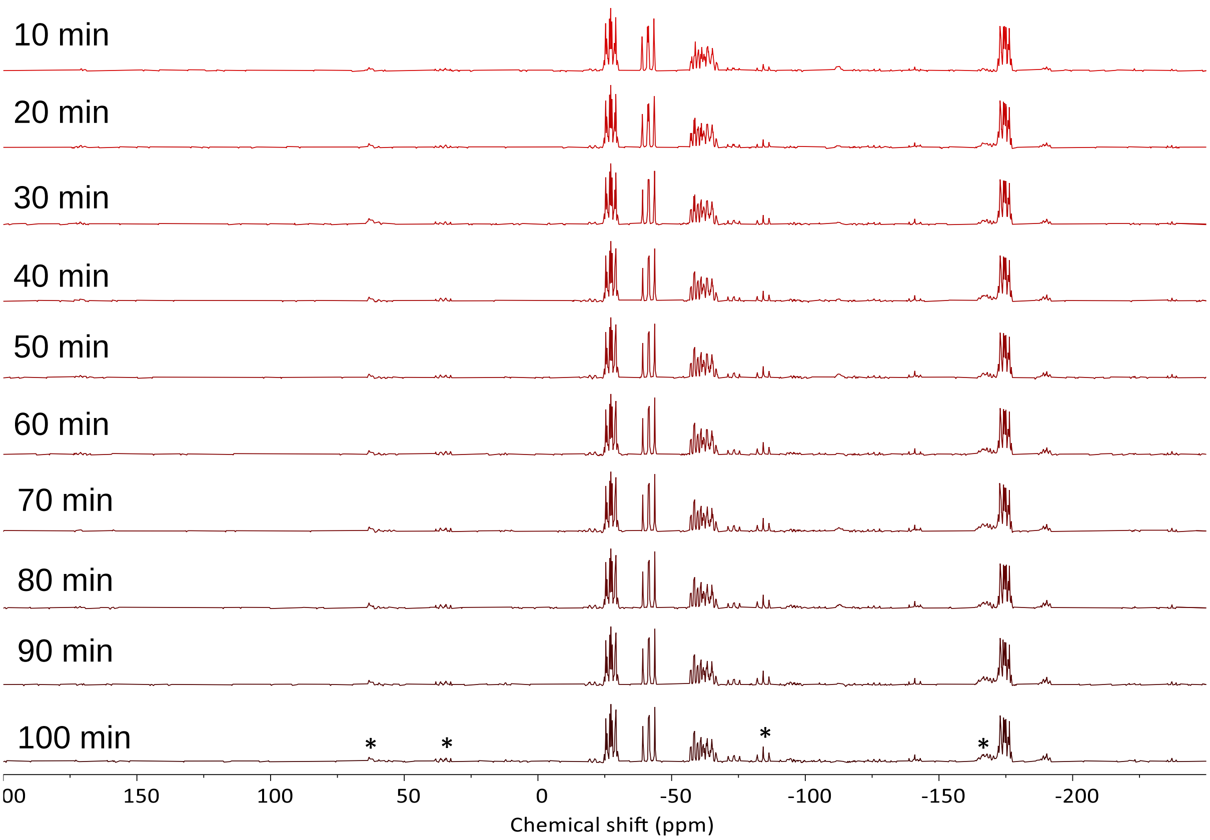

Figure S27. Tracked ^{31}P NMR spectrum (162 MHz, *o*DFB) of $[Na(18c6)]_2$[**2**] heated at 60 °C. Decomposition products marked by *.

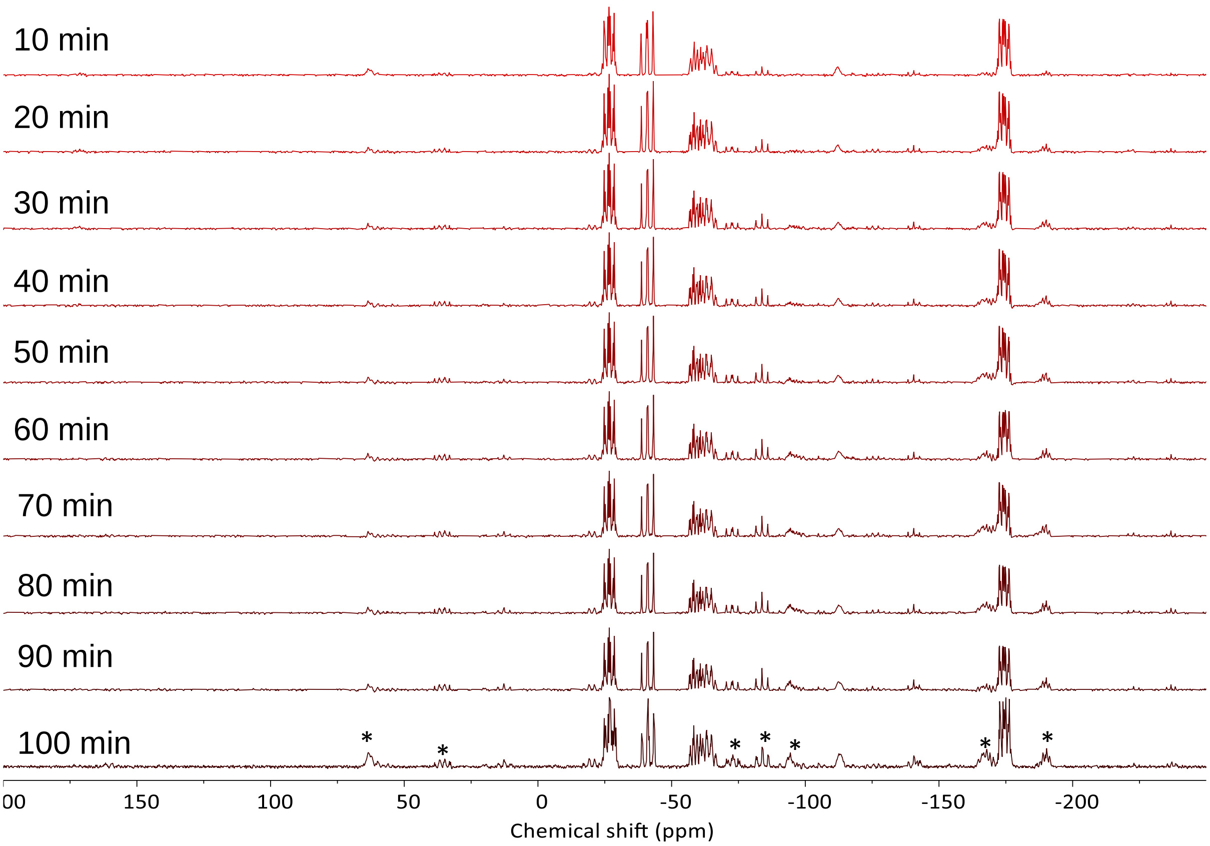

Figure S28. Tracked ^{31}P NMR spectrum (162 MHz, *o*DFB) of $[Na(18c6)]_2$[**2**] heated at 70 °C. Decomposition products marked by *.

3.4. $[Na(18c6)]_2[(iBu_2Al)P_7]$ Catalyst Recycling

$$CO_2 + 1.5\ (HBBN)_2 \xrightarrow[\text{oDFB:Toluene 1:1, RT, overnight}]{[Na(18c6)]_2[\mathbf{2}]\ (0.1\ mol\%)} MeO\text{–}BBN + (BBN)_2O$$

To a J Young NMR tube C_6Me_6 (8.9 mg, 0.055 mmol), HBBN dimer (36 mg, 0.15 mmol, 0.5 eq.), and a solution of $[Na(18c6)]_2$[**2**] (0.3 mg, 0.3 µmol, 0.001 eq.) in *o*DFB: toluene (0.6 mL, 1:1) was added. The reaction mixture was degassed and the headspace was refilled with CO_2 (1 atm). The reaction was monitored by ^1H, ^{11}B and $^{11}B\{^1H\}$ NMR spectroscopy. The NMR conv. was calculated by integration of the crude ^1H NMR spectrum using the C_6Me_6 as an internal standard (^1H δ = 2.20 ppm). Catalyst+HBBN adduct was detected by ^{31}P NMR spectroscopy in an 20x upscale reaction, Figure S29. The tube was reloaded with HBBN dimer and subsequently degassed and the headspace refilled with CO_2 (1 atm). This process was repeated 9 times. The complete recycling experiment was repeated 3 more times. The data (over the 4 runs) was averaged and 95% confidence interval was determined, depicted by the error bars in Figure 3 in the manuscript. The results are tabulated in Table S1. A presentative set of NMR spectra is given below in Figure S30.

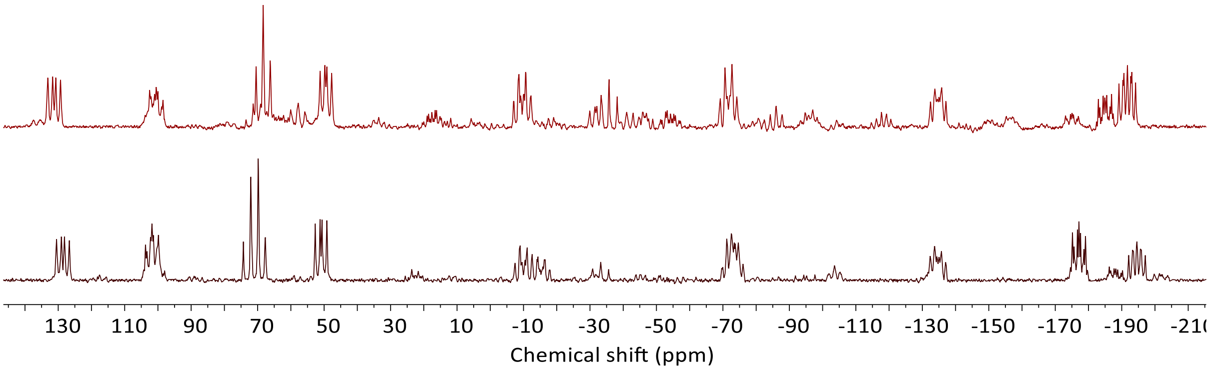

Figure S29. ^{31}P NMR spectrum (162 MHz, reaction mixture) of top: $[Na(18c6)]_2$[**2**] after catalysis (20x upscale reaction); bottom: $[Na(18c6)]_2$[**2**] + HBBN dimer

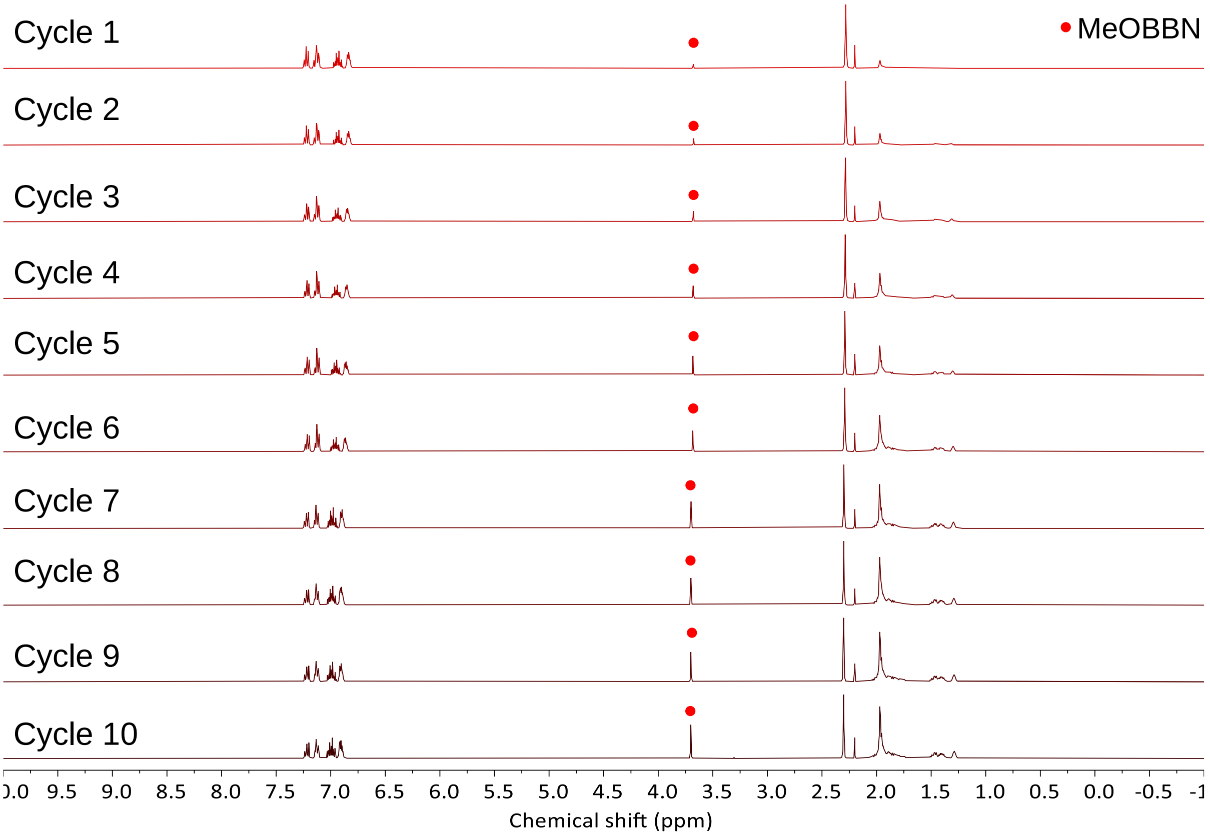

Figure S30. ^{1}H NMR spectra (400 MHz, *o*DFB:toluene) of recycling $[Na(18c6)]_2$[**2**] in the hydroboration of CO_2 using (0.1 mol%) at RT.

Table S1. [Na(18c6)]$_2$[(iBu2Al)P$_7$] Catalyst Recycling

Cycle	Average Conv. MeOBBN	Error	Average Conv. $CH_2(OBBN)_2$	Error
1	95.3	±4.2	4.2	±2.5
2	95.0	±2.1	2.4	±3.1
3	97.5	±2.0	1.2	±2.2
4	98.1	±1.2	0.9	±0.6
5	98.2	±2.3	0.4	±0.8
6	98.4	±0.9	0.0	±0.0
7	98.8	±0.9	0.0	±0.0
8	97.6	±2.3	1.0	±2.0
9	98.1	±1.5	0.6	±1.1
10	98.7	±1.1	0.0	±0.0

3.5. [Na(18c6)]$_2$[(*i*Bu$_2$Al)P$_7$] Catalyst Recovery

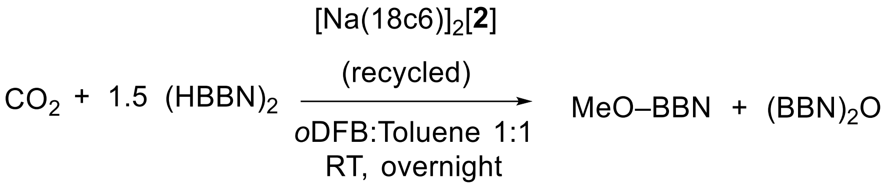

After the 10th cycle in the recycling in the hydroboration of CO_2, the catalyst was isolated from the reaction mixture by removal of volatiles, and washing with pentane. The catalyst was dried and then dissolved in *o*DFB (0.6 mL). To this solution C_6Me_6 (8.5 mg, 0.052 mmol) and HBBN dimer (36 mg, 0.15 mmol) were added and allowed to react at RT for 24 h. The reaction mixture was monitored by ^1H, ^{11}B and $^{11}B\{^1H\}$ NMR spectroscopy. Crude NMR conv. was determined by integration using C_6Me_6 (^1H δ = 2.20 ppm) as internal standard and found to be 99% MeOBBN. The recovery experiment was repeated 3 more times. The data (over the 4 experiments) was averaged (98.4% and 0.1% conv. to MeOBBN and $CH_2(OBBN)_2$ respectively) and 95% confidence interval was determined (error ±1.1 and ±0.3 for MeOBBN and

$CH_2(OBBN)_2$ respectively), depicted by the error bars in Figure 3 in the manuscript. The average A presentative set of NMR spectra is given below in Figures S31 and S32.

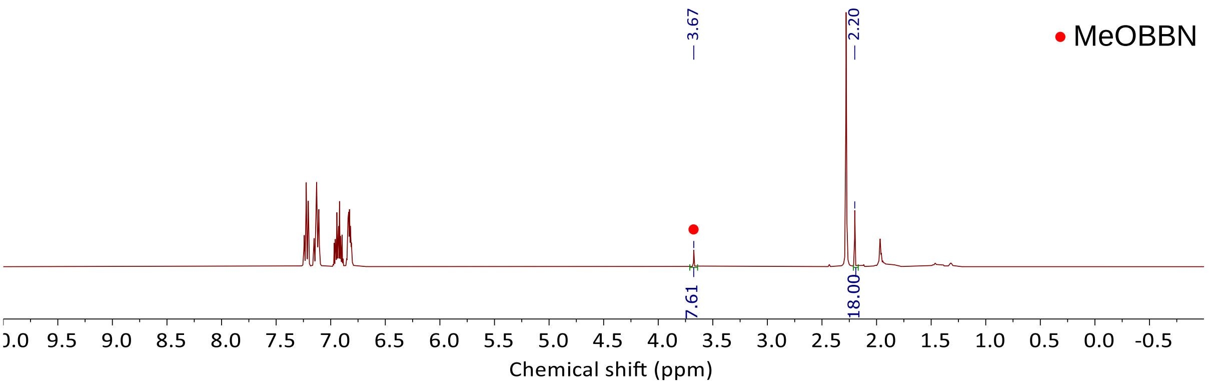

Figure S31. ^1H NMR spectrum (400 MHz, *o*DFB:toluene) of using recovered $[Na(18c6)]_2$[**2**] in the hydroboration of CO_2.

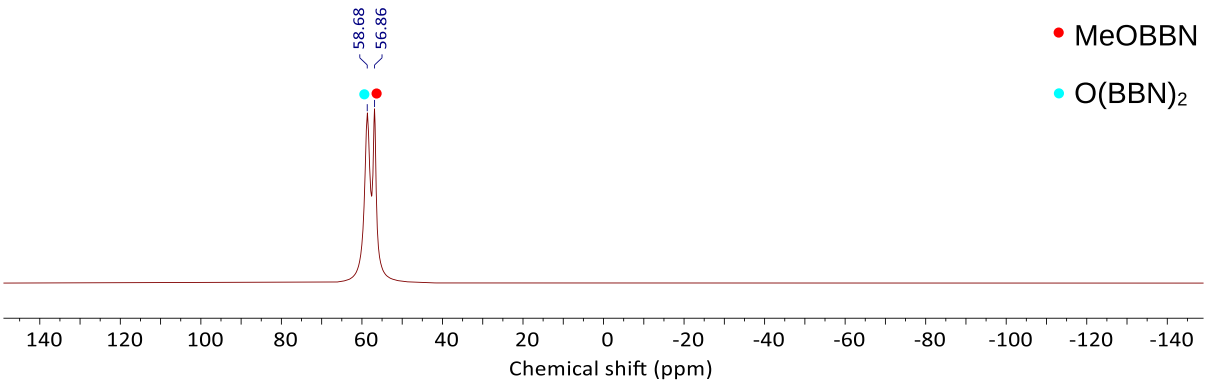

Figure S32. ^{11}B NMR spectrum (128 MHz, *o*DFB:toluene) of using recovered $[Na(18c6)]_2$[**2**] in the hydroboration of CO_2.

3.6. Comparison to Literature Metal-free Catalysts for CO_2 Hydroboration

Table S2. Comparison of TON and TOF for Metal-free Catalysts Reported for CO_2 Hydroboration

$$CO_2 + H\text{—}[B] \xrightarrow{\textbf{Cat}} MeO[B]$$

Catalysts [ref]	H–[B]	Solvent	Pressure CO_2 (atm)	T (°C)	Cat loading (mol%)	TON	TOF h^{-1}
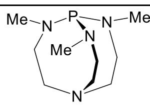 [10]	HBBN dimer	THF	1	20	0.01	6043	32
				70	0.5	100	287
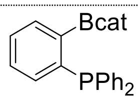 [11]	$BH_3 \cdot SMe_2$	C_6D_6	2	70	0.33	2950	737
					0.1	853	853
$^tBu_3P$ [12]	BBN	C_6H_5Br	3	60	0.02	5556	176
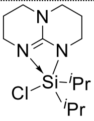 [13]	HBpin	THF	1	90	2.5	32	2
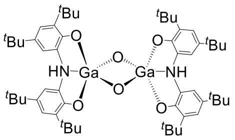 [14]	HBpin	C_6D_6	2	RT	1.0	99	2.6
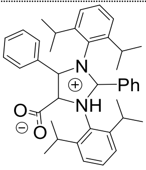 [15]	HBBN dimer	C_6D_6	1	RT	0.1	300	50
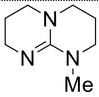 [16]	HBBN dimer	C_6D_6	1	RT	0.1	648	33
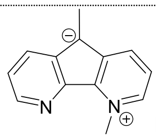 [17]	$BH_3 \cdot SMe_2$	$CDCl_3$	1.5	RT	1	298	43
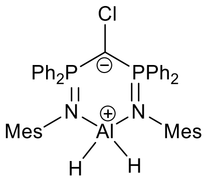 [18]	$BH_3 \cdot SMe_2$	C_6D_6	1	110	1	293	293
					10	30	356

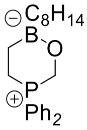 [19]	HBBN dimer	C_6D_6	1	60	0.1	341<br>177	49<br>177
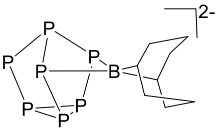 2 $[Na(18c6)]^+$ 5	HBBN dimer	*o*DFB: Tol	1	RT<br>50	0.01<br>0.33	9800<br>300	20<br>300

3.7. Comparison to Literature Metal Catalysts for CO_2 Hydroboration

Table S3. Comparison of TON and TOF for Metal Catalysts Reported for CO_2 Hydroboration.

$$CO_2 + H{-}[B] \xrightarrow{\textbf{Cat}} MeO[B]$$

Catalysts	H–[B]	Solvent	Pressure CO_2 (atm)	T (°C)	Cat loading (mol%)	TON	TOF h^{-1}
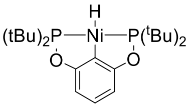 20	HBCat	C_6D_6	1	25	0.5	495	495
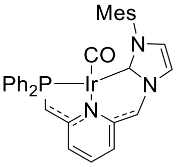 21	HBCat	THF	2	30	1	84	56
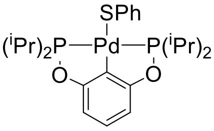 21	HBCat	C_6H_6	1	RT	1	445	1780
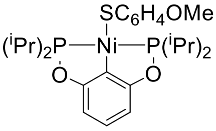 22	HBCat	C_6D_6	1	RT	0.2	490	2400
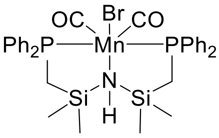 23	HBpin	neat	1	100	0.036	883	63

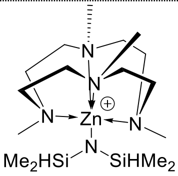 24	HBpin	THF	1	60	10	100	6.25
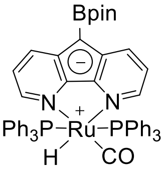 25	HBpin	C_6D_5Br	1.5	100	1	54	72
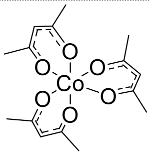 + $NaHBEt_3$ 26	$BH_3 \cdot SMe_2$	THF	1	50	0.33	297	15
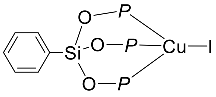 *P* = PPh_2 27	$(HBBN)_2$	CH_3CN	1	60	1.5	59	14.8

Tables in 3.6 and 3.7 do not represent comprehensive lists. Instead, these lists serve to help demonstrate the relative reactivity to the clusters reports *vs.* other documented homogenous metal and metal-free catalysts in CO_2 hydroborative reductions that are selective for methoxyborane formation under similar conditions.

4. Experimental Mechanistic studies

4.1. Stoichiometric Reactions

The addition of benzaldehyde, acetophenone, phenyl isocyanate, CO_2, HBpin and HBBN dimer to [Na(18c6)]$_2$[(BBN)P_7] has been reported previously.[5]

4.1.1. Addition of HBin to [Na(18c6)]$_2$[2]

To a J Young NMR tube a solution of [Na(18c6)]$_2$[**2**] (25 mg, 0.027 mmol, 1 eq.) in *o*DFB and HBpin (4 µL, 0.027 mmol, 1 eq.) was added and allowed to react for 24h. The reaction was monitored by ^{11}B and ^{31}P NMR spectroscopy. No reaction was observed after 24h.

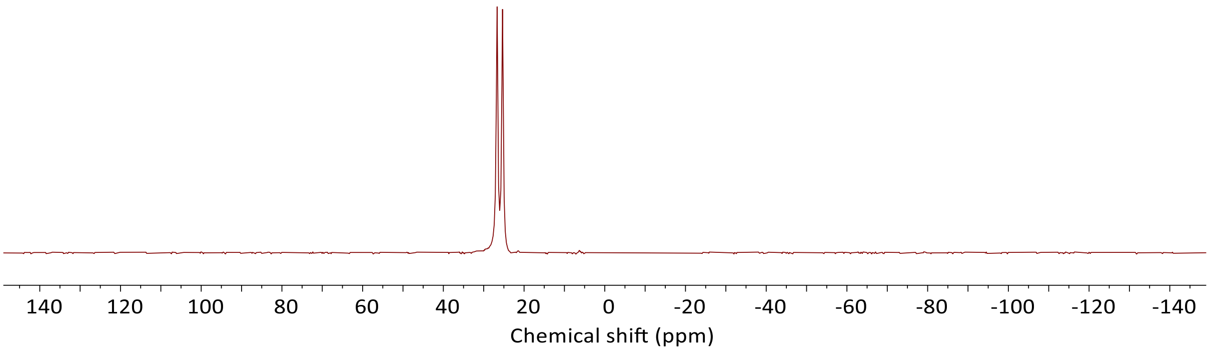

Figure S33. ^{11}B NMR spectrum (128 MHz, *o*DFB) of [Na(18c6)]$_2$[**2**] + HBpin.

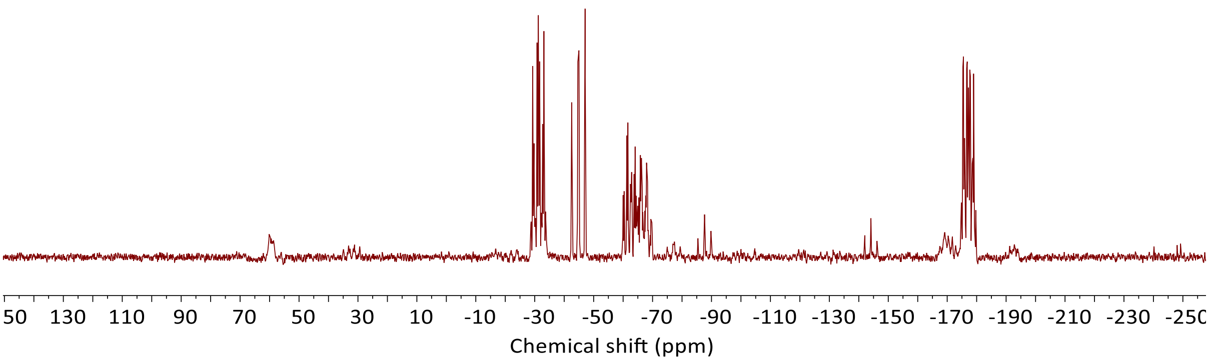

Figure S34. ^{31}P NMR spectrum (162 MHz, *o*DFB) of [Na(18c6)]$_2$[**2**] + HBpin.

4.1.2. Addition of HBBN to [Na(18c6)]$_2$[2]

To a J Young NMR tube a solution of [Na(18c6)]$_2$[**2**] (25 mg, 0.027 mmol, 1 eq.) in *o*DFB and HBBN dimer (3.4 mg, 0.014 mmol, 0.5 eq.) was added and allowed to react for 24h. The reaction was monitored by ^{11}B and ^{31}P NMR spectroscopy. No reaction was observed after 1h, only after leaving the reaction to react for >10 h.

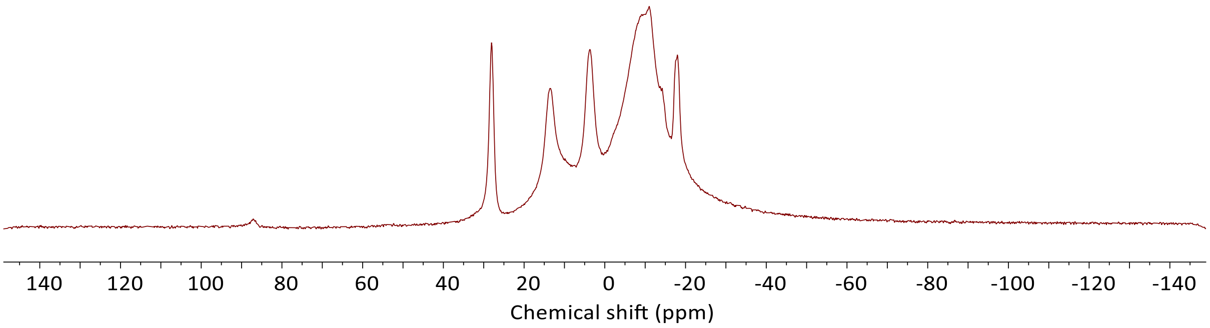

Figure S35. ^{11}B NMR spectrum (128 MHz, *o*DFB) of [Na(18c6)]$_2$[**2**] + HBBN.

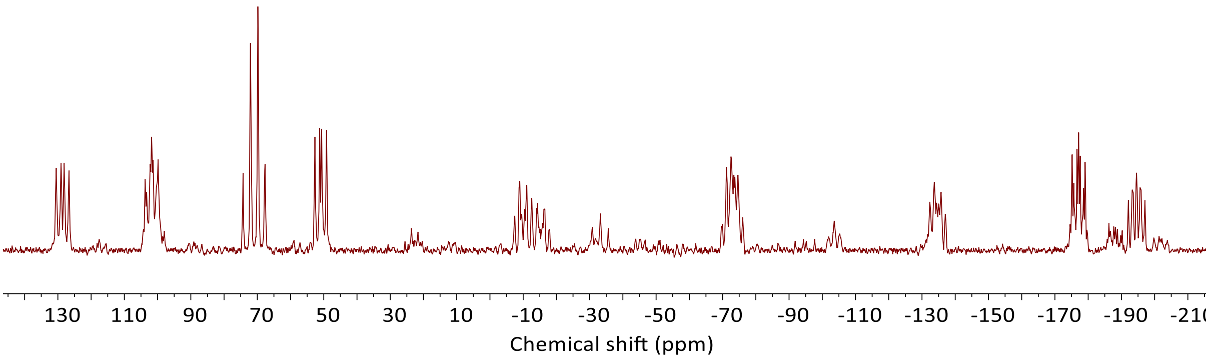

Figure S36. ^{31}P NMR spectrum (162 MHz, *o*DFB) of [Na(18c6)]$_2$[**2**] + HBBN.

4.1.3. Addition of Benzaldehyde to [Na(18c6)]2[2]

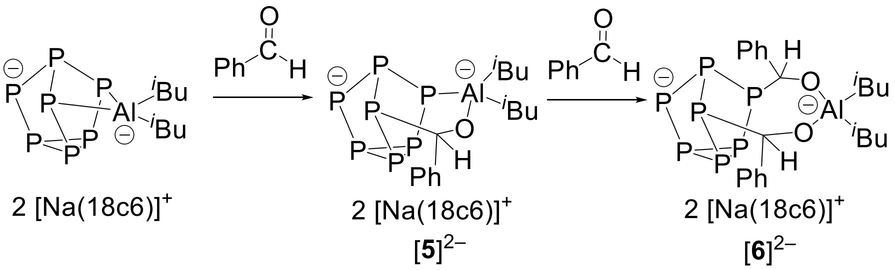

To a J Young NMR tube a solution of $[Na(18c6)]_2$[**2**] (25 mg, 0.027 mmol, 1 eq.) in *o*DFB and benzaldehyde (1.4 µL, 0.013 mmol, 0.5 eq.) was added and allowed to react for 30 min. The reaction was monitored by ^{1}H and ^{31}P NMR spectroscopy and the product distribution was found to be 4:72:24 [**2**]$^{2-}$:[**5**]$^{2-}$:[**6**]$^{2-}$ by ^{31}P NMR spectroscopy. More benzaldehyde (1.4 µL, 0.013 mmol, +0.5 eq.) was added and allowed to react for 30 min. Again the reaction was monitored by ^{1}H and ^{31}P NMR spectroscopy and the product distribution was found to be 11:23:66 [**2**]$^{2-}$:[**5**]$^{2-}$:[**6**]$^{2-}$ by ^{31}P NMR spectroscopy. Finally, more benzaldehyde (2.8 µL, 0.027 mmol, +1 eq.) was added and allowed to react for 30 min. The reaction was monitored by ^{1}H and ^{31}P NMR spectroscopy, full conversion to [**6**]$^{2-}$ was observed. Spectra were referenced against C_6Me_6 internal standard (^{1}H δ = 2.20).

Further detailed IR and ^{13}C NMR spectroscopy studies are given below.

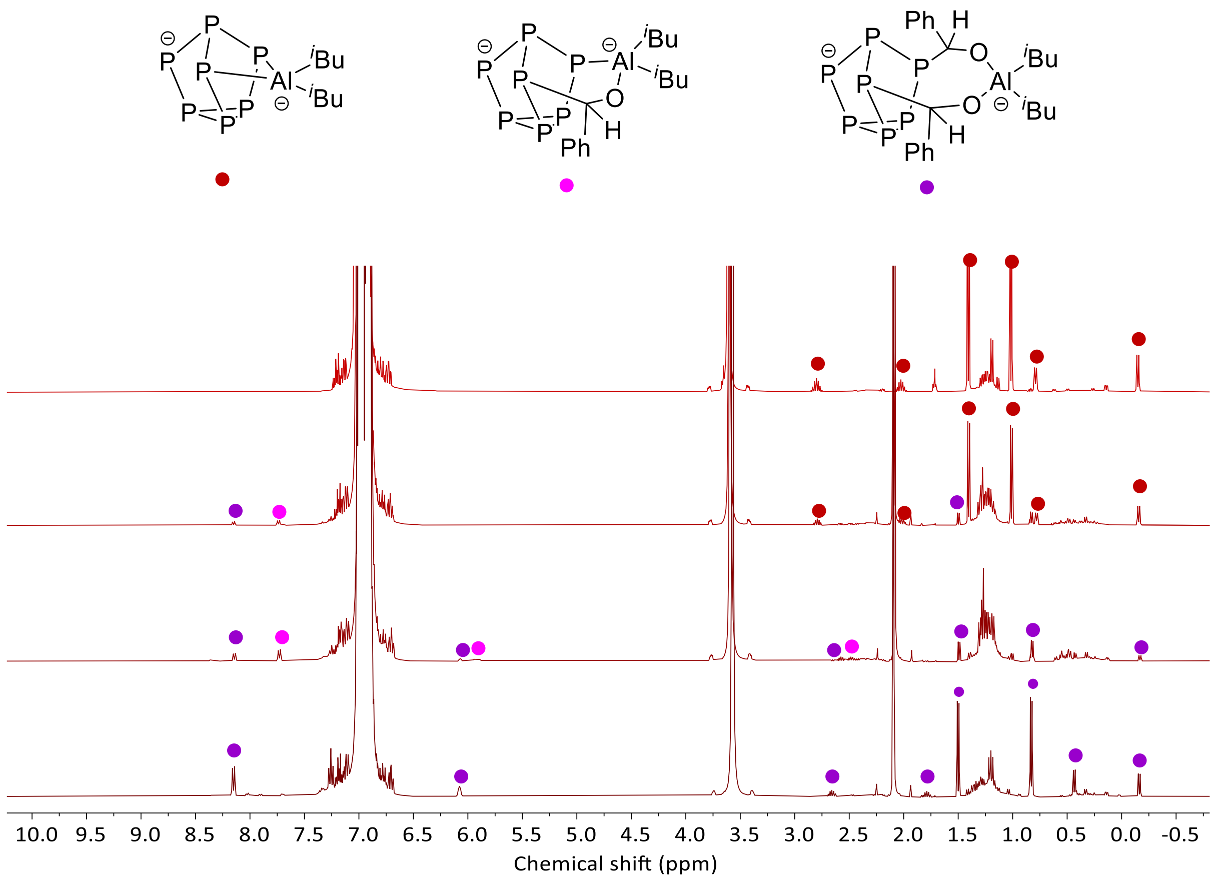

Figure S37. ^{1}H NMR spectrum (400 MHz, *o*DFB) of [Na(18c6)]$_2$[**2**] + PhCH(=O). Top spectrum: [Na(18c6)]$_2$[**2**]; second spectrum: [Na(18c6)]$_2$[**2**] + 0.5 eq. PhCH(=O); third spectrum: [Na(18c6)]$_2$[**2**] + 1 eq. PhCH(=O); bottom spectrum: [Na(18c6)]$_2$[**2**] + 2 eq. PhCH(=O).

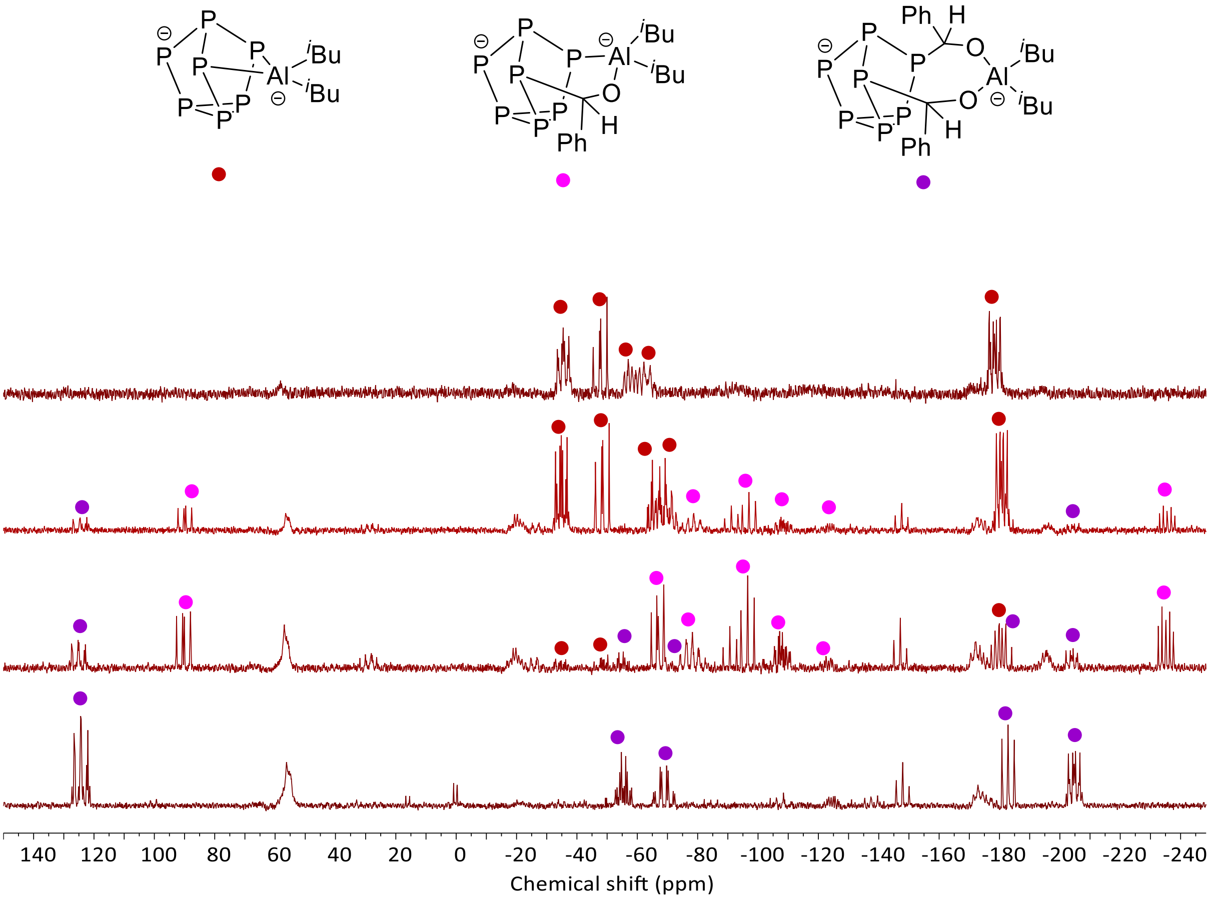

Figure S38. ^{31}P NMR spectrum (162 MHz, *o*DFB) of [Na(18c6)]$_2$[**2**] + PhCH(=O). Top spectrum: [Na(18c6)]$_2$[**2**]; second spectrum: [Na(18c6)]$_2$[**2**] + 0.5 eq. PhCH(=O); third spectrum: [Na(18c6)]$_2$[**2**] + 1 eq. PhCH(=O); bottom spectrum: [Na(18c6)]$_2$[**2**] + 2 eq. PhCH(=O).

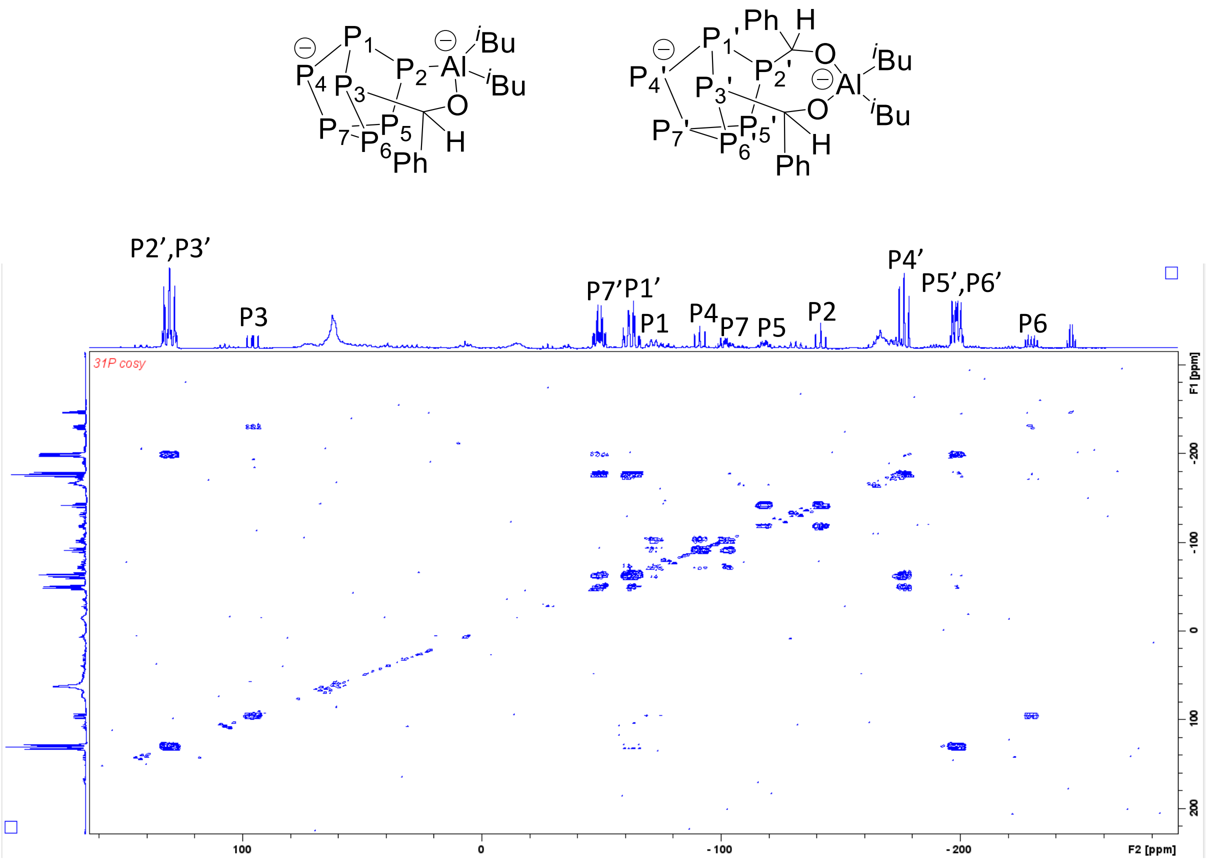

Figure S39. ^{31}P COSY NMR spectrum (162 MHz, *o*DFB) of [Na(18c6)]$_2$[**2**] + 1 eq. PhCH(=O).

Infrared spectroscopy studies

For IR spectroscopy studies the reactions above was repeat twice using 25 mg, 0.027 mmol [Na(18c6)]$_2$[**2**], once with addition of 1 eq. PhCHO (2.8 µL, 0.027 mmol) and once with the addition of 2 eq. PhCHO (5.6 µL, 0.054 mmol). The reaction mixtures were monitored by ^{31}P NMR spectroscopy. When completion was observed (30 min), the solvent was removed *in vacuo* and the residue was washed with 10 mL pentane. After the residue was dried in vacuo, the reaction mixtures were introduced onto the IR spectrometer as glassy solids. [Na(18c6)]$_2$[κ^2-(iBu$_2$Al)P$_7$] was also introduced on the IR spectrometer as a solid, whereas PhCHO was introduced as a liquid.

The distinct C–O stretch of [**5**]$^{2–}$ and [**6**]$^{2–}$ are largely obscured by the 18c6 sequestering agent and only two shouldering signals could be observed (v = 1063 and 1025 cm$^{–1}$). The IR spectra are however consisted with the disappearance of the C=O stretch of benzaldehyde (v = 1695 cm$^{–1}$) and the incorporation of aryl C=C stretches in the products (v = 1618 and 1595 cm$^{–1}$). Further strong signals for aryl C–H bending (v = 760 and 700 cm$^{–1}$) can be observed in the fingerprint region for the products.

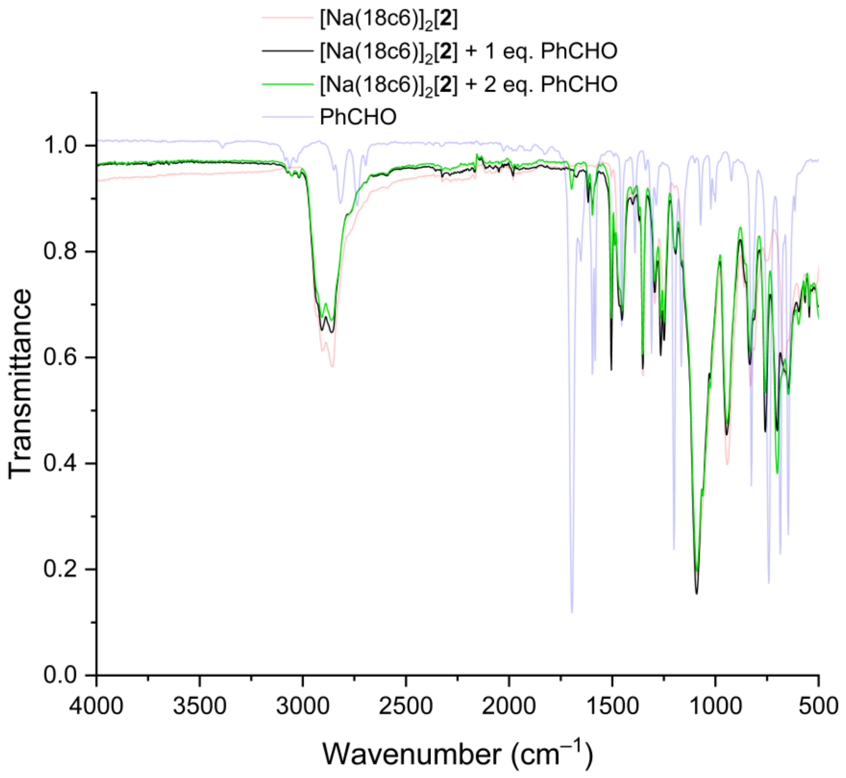

Figure S40. IR spectrum addition of benzaldehyde to [Na(18c6)]$_2$[**2**] (range 4000-500 cm$^{–1}$).

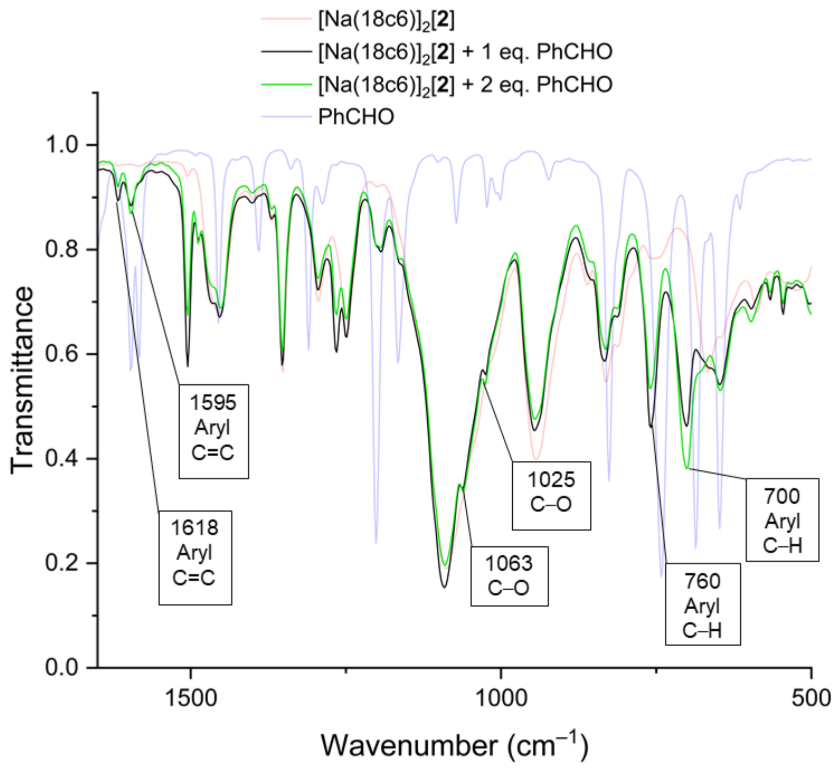

Figure S41. IR spectrum addition of benzaldehyde to $[Na(18c6)]_2$[**2**] with assignment (range 1650-500 cm^{-1}).

^{13}C NMR and ^{1}H^{13}C HSQC NMR spectroscopy studies

For these ^{13}C NMR studies, the reactions above were repeat twice using 50 mg, 0.054 mmol $[Na(18c6)]_2$[**2**], once with addition of 1 eq. PhCHO (5.6 µL, 0.054 mmol) and once with the addition of 2 eq. PhCHO (11.2 µL, 0.108 mmol). The reaction mixtures were monitored by ^{1}H and ^{31}P NMR spectroscopy. When completion was observed (30 min), the reaction mixtures were investigated by ^{13}C, ^{13}C{^{1}H}, ^{13}C DEPT135, and ^{1}H^{13}C HSQC NMR spectroscopy. Detailed spectra assignment of the ^{13}C and ^{13}C{^{1}H} spectra below in Figures S48 and S49.

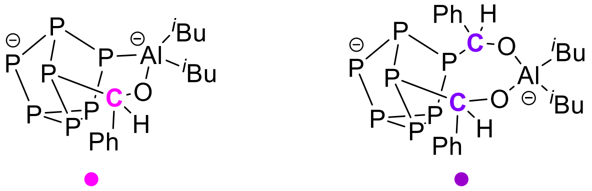

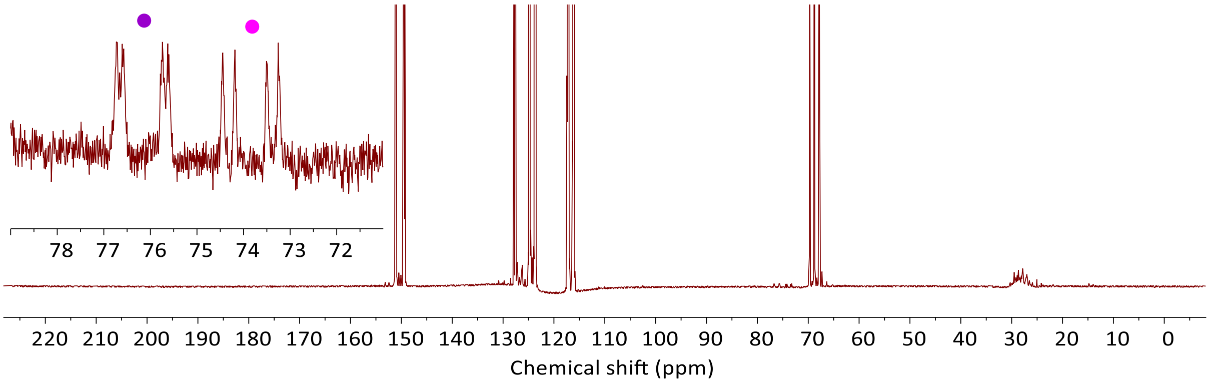

Figure S42. ^{13}C NMR spectrum (151 MHz, *o*DFB) of [Na(18c6)]$_2$[**2**] + 1 eq. PhCH(=O).

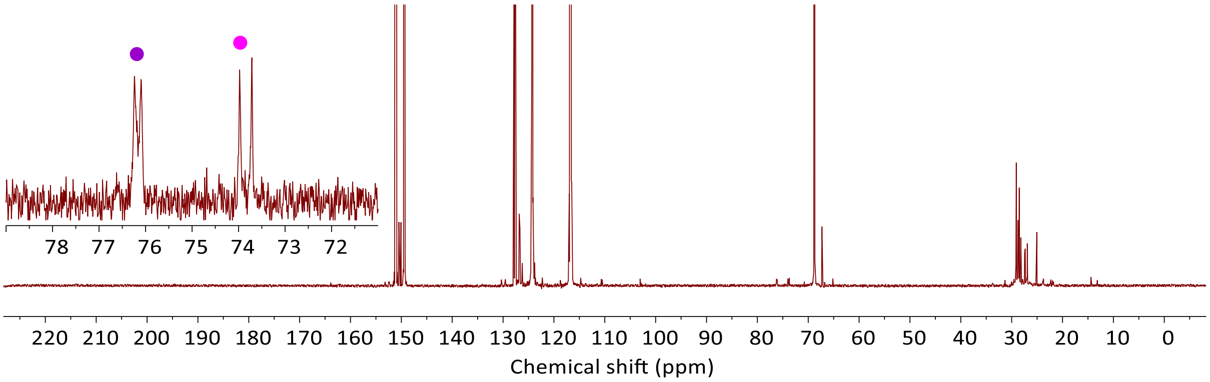

Figure S43. ^{13}C{^{1}H} NMR spectrum (151 MHz, *o*DFB) of [Na(18c6)]$_2$[**2**] + 1 eq. PhCH(=O).

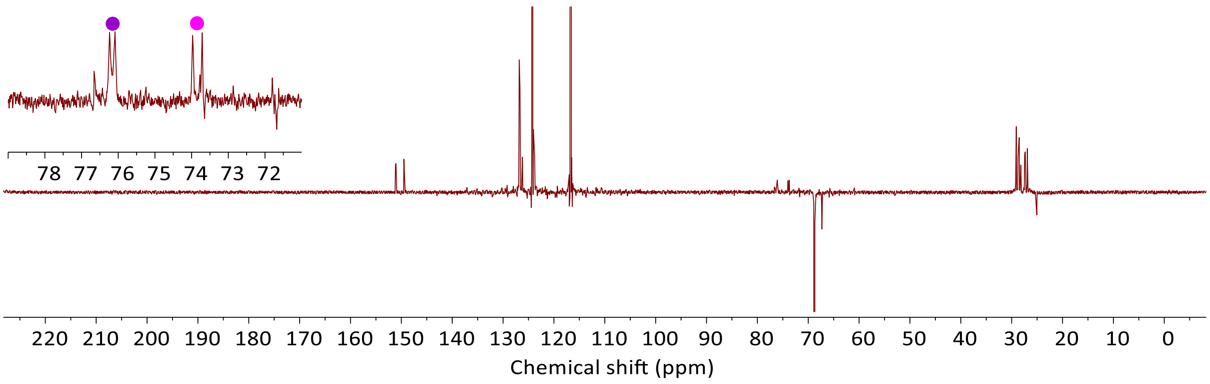

Figure S44. ^{13}C DEPT135 NMR spectrum (151 MHz, *o*DFB) of $[Na(18c6)]_2$[**2**] + 1 eq. PhCH(=O).

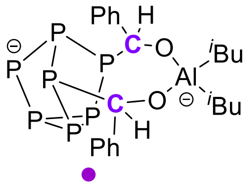

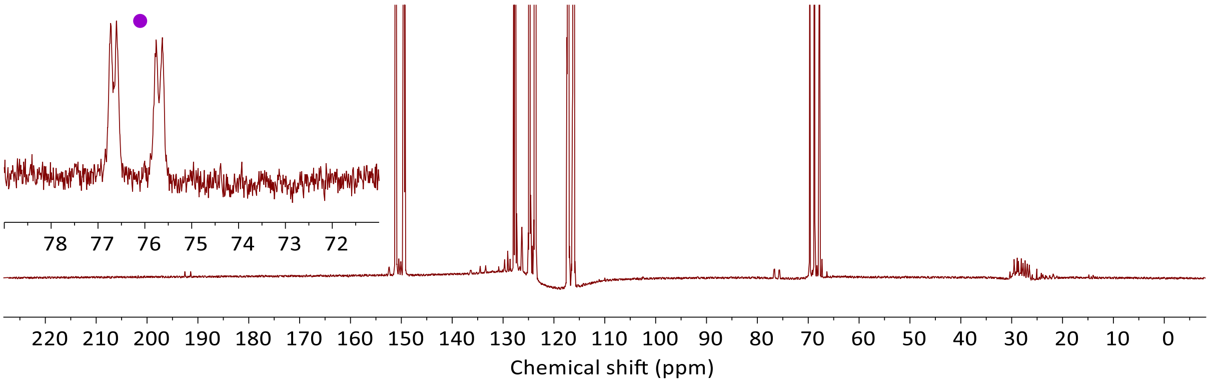

Figure S45. ^{13}C NMR spectrum (151 MHz, *o*DFB) of $[Na(18c6)]_2$[**2**] + 2 eq. PhCH(=O).

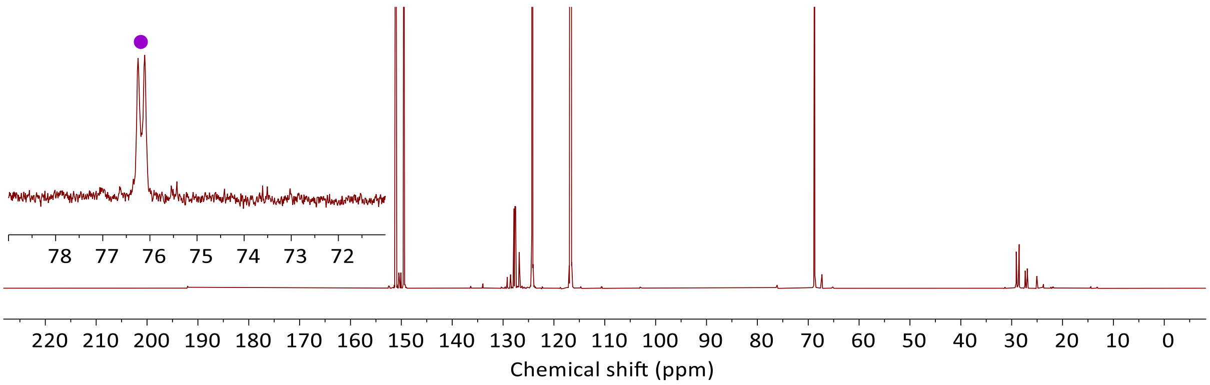

Figure S46. $^{13}C\{^1H\}$ NMR spectrum (151 MHz, *o*DFB) of $[Na(18c6)]_2$[**2**] + 2 eq. PhCH(=O).

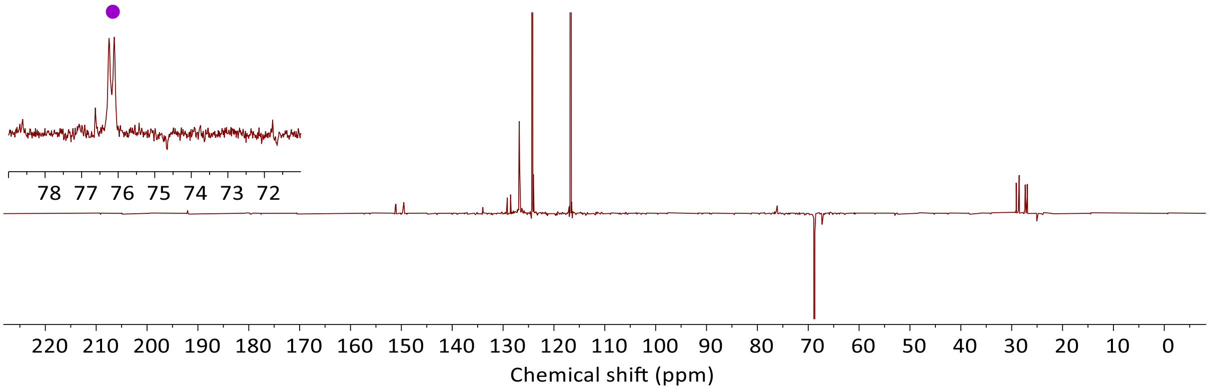

Figure S47. ^{13}C DEPT135 NMR spectrum (151 MHz, *o*DFB) of $[Na(18c6)]_2$[**2**] + 2 eq. PhCH(=O).

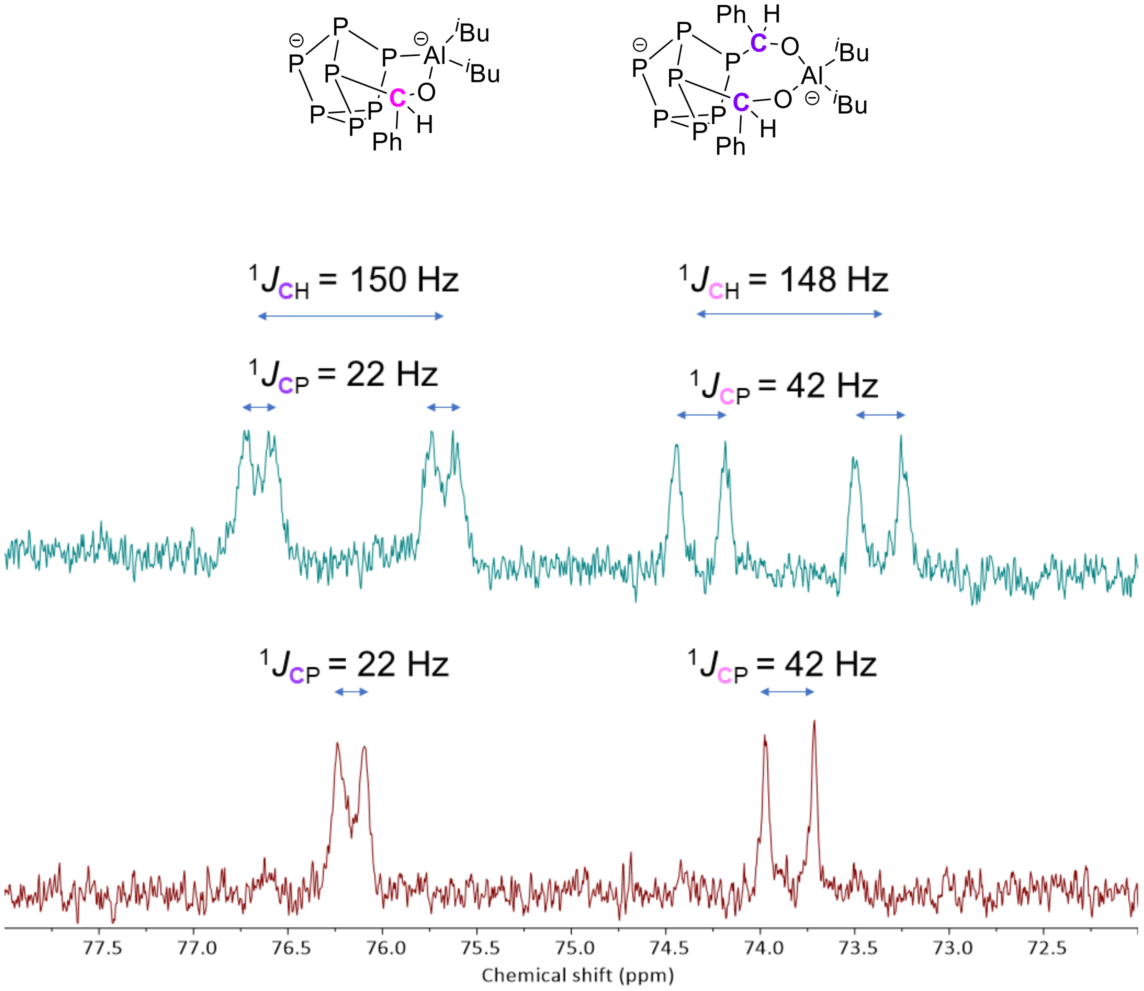

Figure S48. Detailed NMR spectrum (151 MHz, *o*DFB) of [Na(18c6)]$_2$[**2**] + 1 eq. PhCH(=O). Top: ^{13}C NMR spectrum. Bottom: ^{13}C{^{1}H} NMR spectrum.

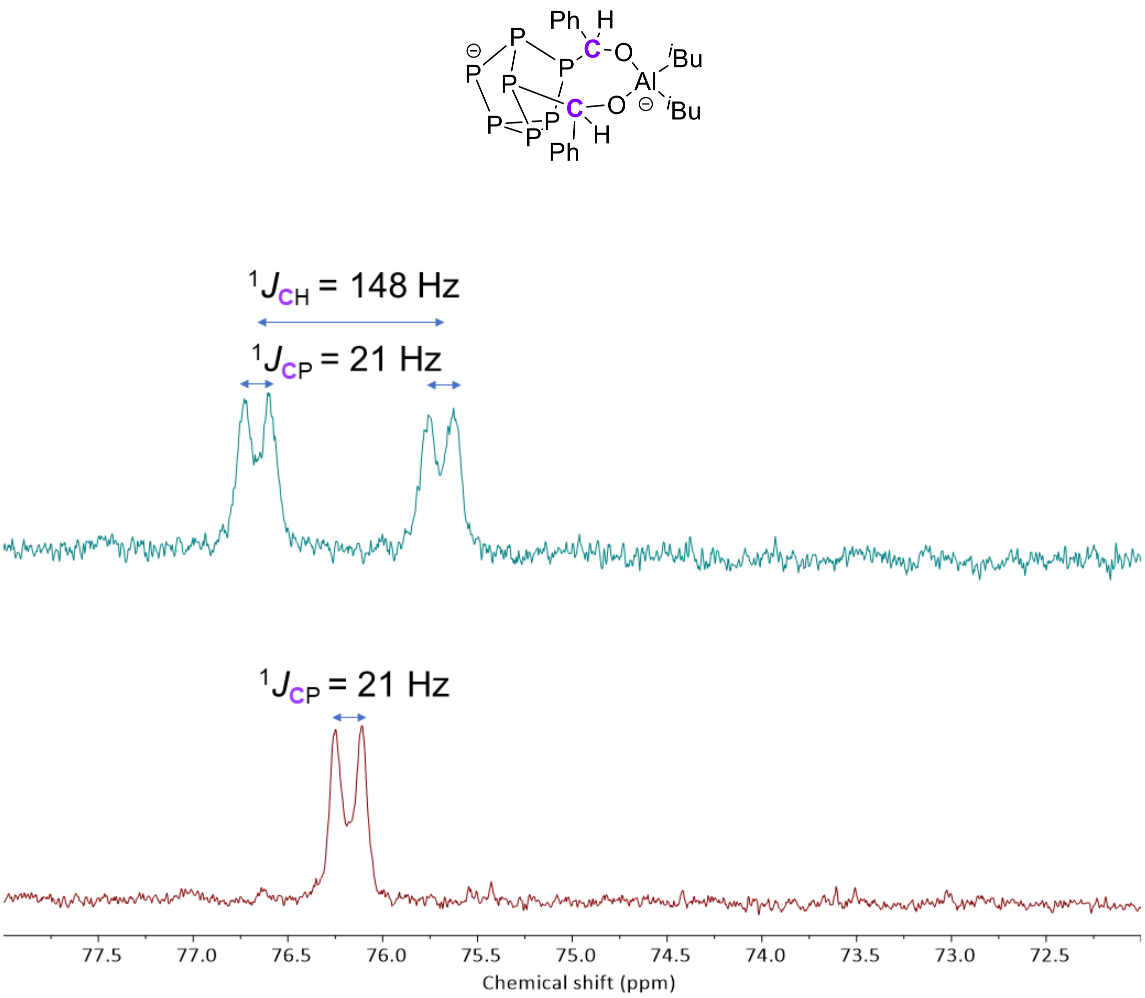

Figure S49. Detailed NMR spectrum (151 MHz, *o*DFB) of $[Na(18c6)]_2$[**2**] + 2 eq. PhCH(=O). Top: ^{13}C NMR spectrum. Bottom: $^{13}C\{^1H\}$ NMR spectrum.

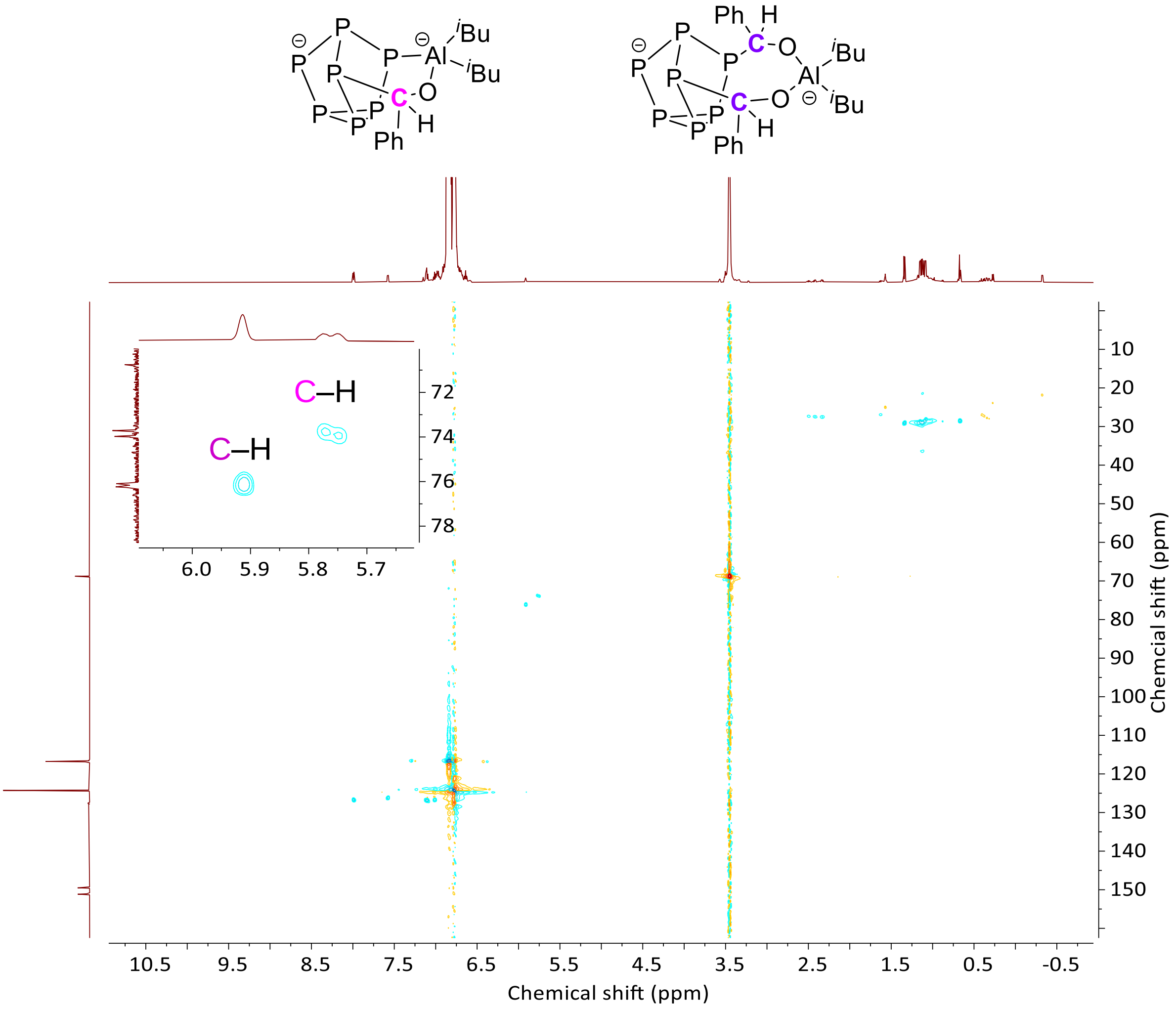

Figure S50. $^{1}H^{13}C$ HSQC NMR spectrum (600 MHz; 151 MHz, *o*DFB) of [Na(18c6)]$_2$[**2**] + 1 eq. PhCH(=O).

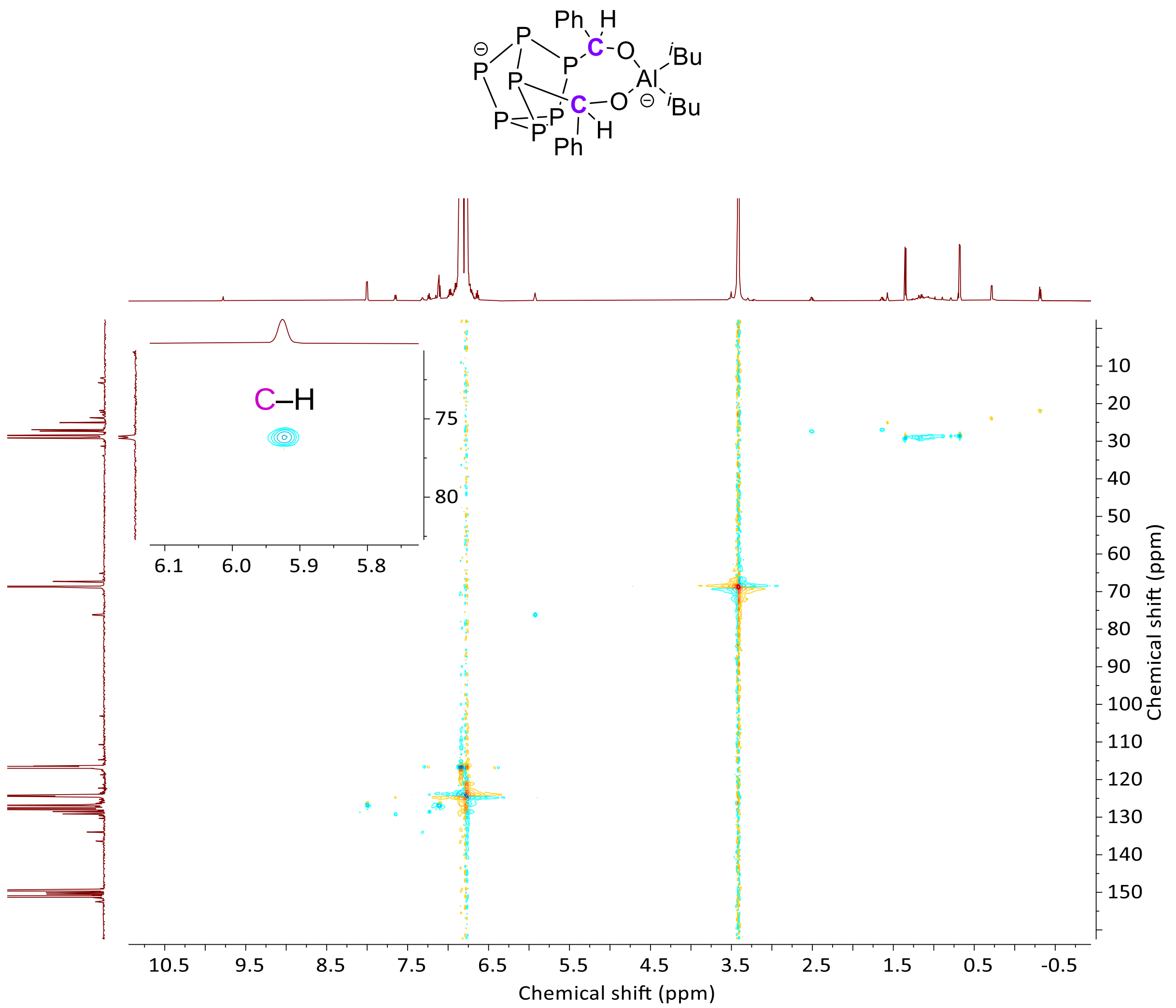

Figure S51. $^{1}H^{13}C$ HSQC NMR spectrum (600 MHz; 151 MHz, *o*DFB) of $[Na(18c6)]_2$[**2**] + 2 eq. PhCH(=O).

4.1.4. Probing Equilibrium Mono- and Bis-insertion of Benzaldehyde into [2]$^{2-}$

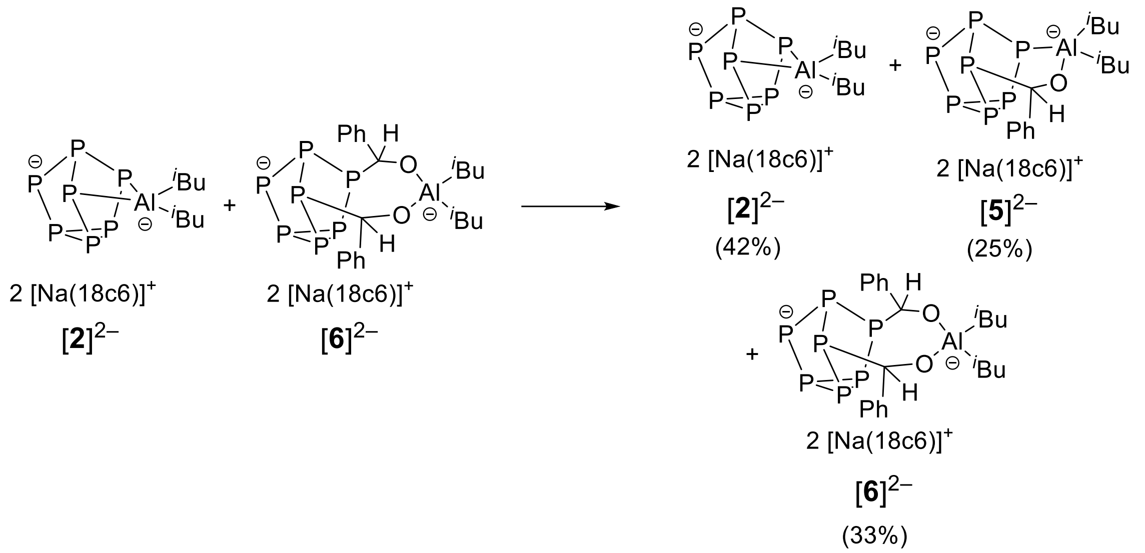

To a J Young NMR tube a solution of [Na(18c6)]$_2$[**2**] (25 mg, 0.027 mmol, 1 eq.) in *o*DFB and benzaldehyde (5.6 µL, 0.056 mmol, 2 eq.) were added. The reaction was monitored by ^{1}H and ^{31}P NMR spectroscopy to confirm complete conversion to [Na(18c6)]$_2$[**6**]. A solution of [Na(18c6)]$_2$[**2**] (25 mg, 0.027 mmol, +1 eq.) in *o*DFB was added to the reaction mixture and then monitored by ^{1}H and ^{31}P NMR spectroscopy and the product distribution was found to be 42:25:33 [**2**]$^{2-}$:[**5**]$^{2-}$:[**6**]$^{2-}$ by ^{31}P NMR spectroscopy.

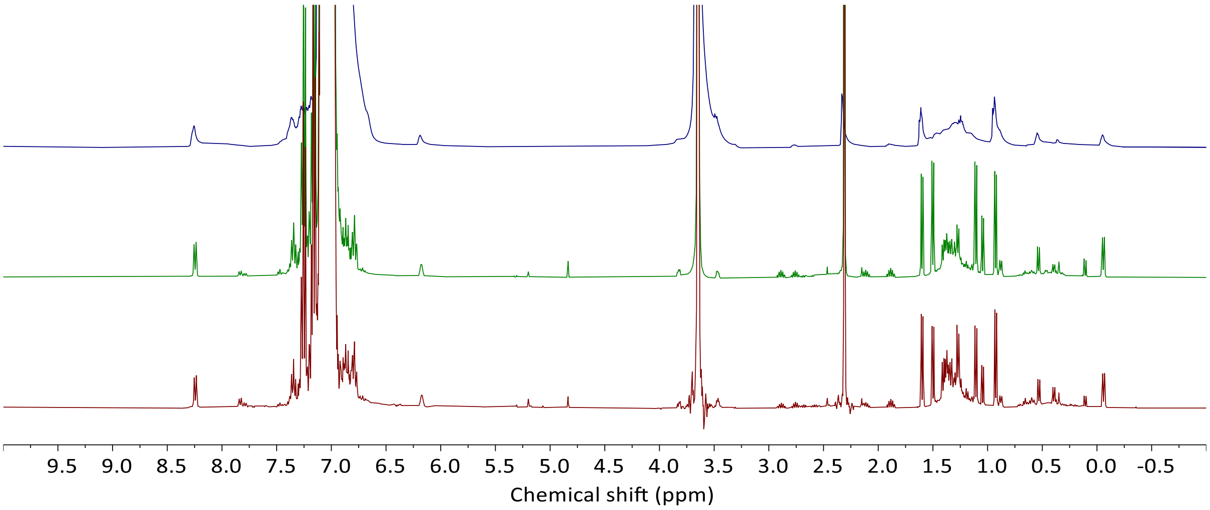

Figure S52. ^{1}H NMR spectrum (400 MHz, *o*DFB) of the addition of [Na(18c6)]$_2$[**2**] to a solution of [Na(18c6)]$_2$[**6**]. Top: solution of just [Na(18c6)]$_2$[**6**]; middle: after addition of [Na(18c6)]$_2$[**2**] to a solution of [Na(18c6)]$_2$[**6**] (3 h); bottom: after addition of [Na(18c6)]$_2$[**2**] to a solution of [Na(18c6)]$_2$[**6**] (4 days).

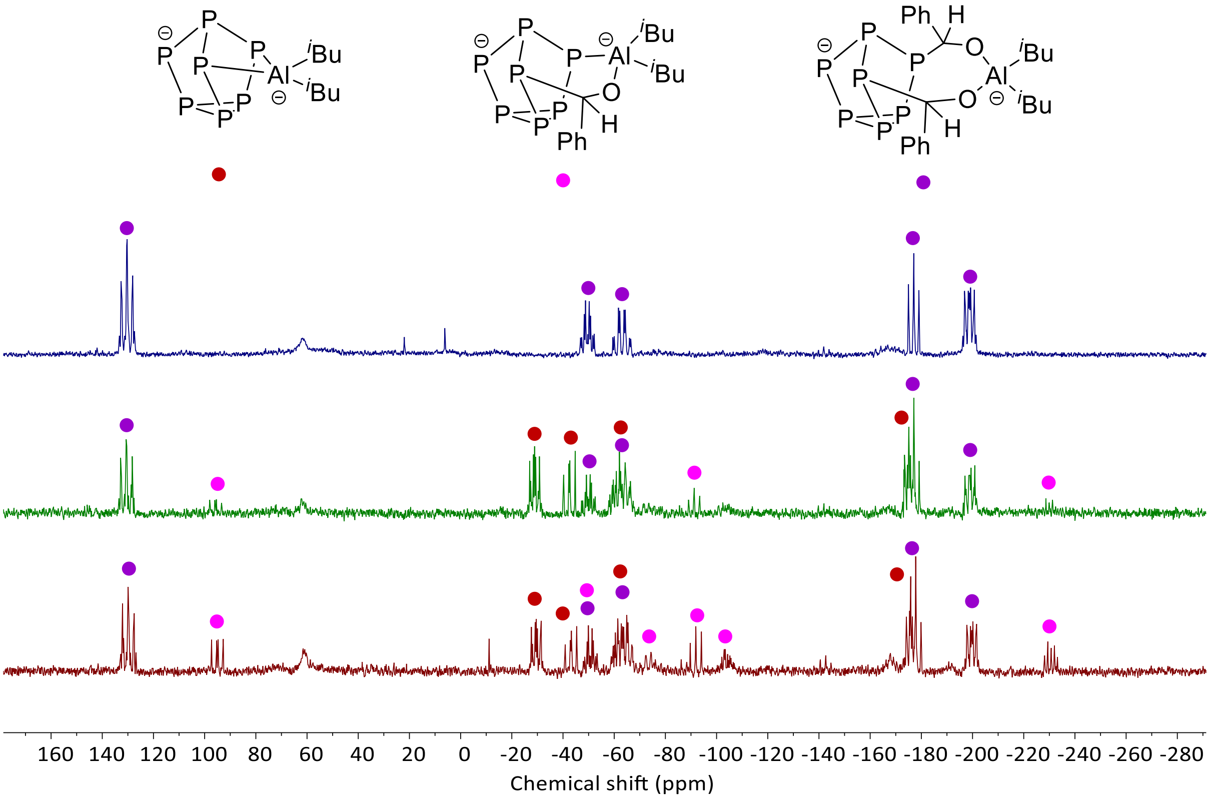

Figure S53. ^{31}P NMR spectrum (162 MHz, *o*DFB) of the addition of [Na(18c6)]$_2$[**2**] to a solution of [Na(18c6)]$_2$[**6**]. Top: solution of just [Na(18c6)]$_2$[**6**]; middle: after addition of [Na(18c6)]$_2$[**2**] to a solution of [Na(18c6)]$_2$[**6**] (3 h); bottom: after addition of [Na(18c6)]$_2$[**2**] to a solution of [Na(18c6)]$_2$[**6**] (4 days).

4.1.5. Addition of Acetophenone to [Na(18c6)]$_2$[2]

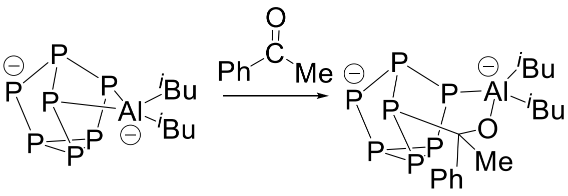

2 [Na(18c6)]$^+$ 2 [Na(18c6)]$^+$

[**7**]$^{2-}$

To a J Young NMR tube a solution of [Na(18c6)]$_2$[**2**] (25 mg, 0.027 mmol, 1 eq.) in *o*DFB and acetophenone (3.2 µL, 0.027 mmol, 1 eq.) was added and allowed to react for 30 min. The reaction was monitored by ^{31}P NMR spectroscopy. More benzaldehyde (3.2 µL, 0.027 mmol, +1 eq.) was added and allowed to react for 24h. Again the reaction was monitored by ^{31}P NMR spectroscopy.

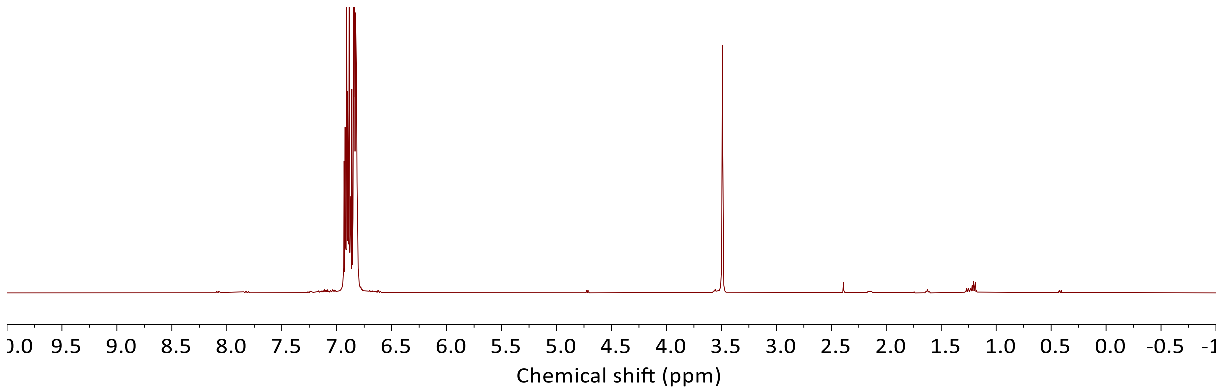

Figure S54. ^{1}H NMR spectrum (400 MHz, *o*DFB) of $[Na(18c6)]_2$[**2**] + 1 eq. PhCMe(=O). Note: the disappearance of the resonances associated for $[Na(18c6)]_2$[**2**].

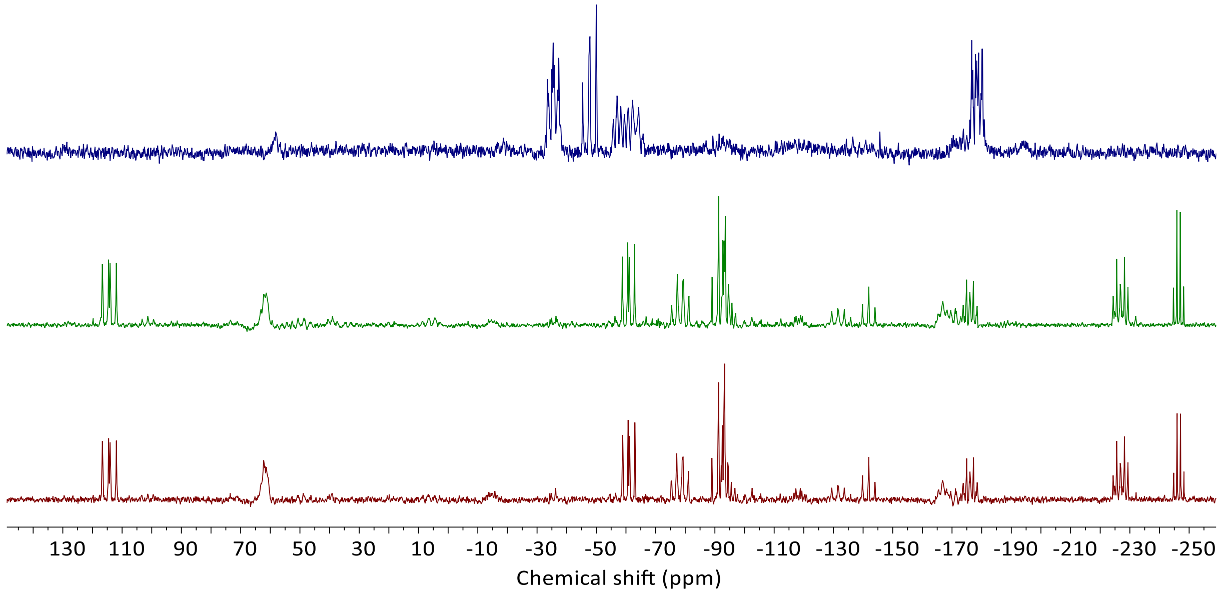

Figure S55. ^{1}H NMR spectrum (400 MHz, *o*DFB) of $[Na(18c6)]_2$[**2**] + PhCMe(=O). Top spectrum: $[Na(18c6)]_2$[**2**]; middle spectrum: $[Na(18c6)]_2$[**2**] + 1 eq. PhCH(=O); bottom spectrum: $[Na(18c6)]_2$[**2**] + 2 eq. PhCMe(=O).

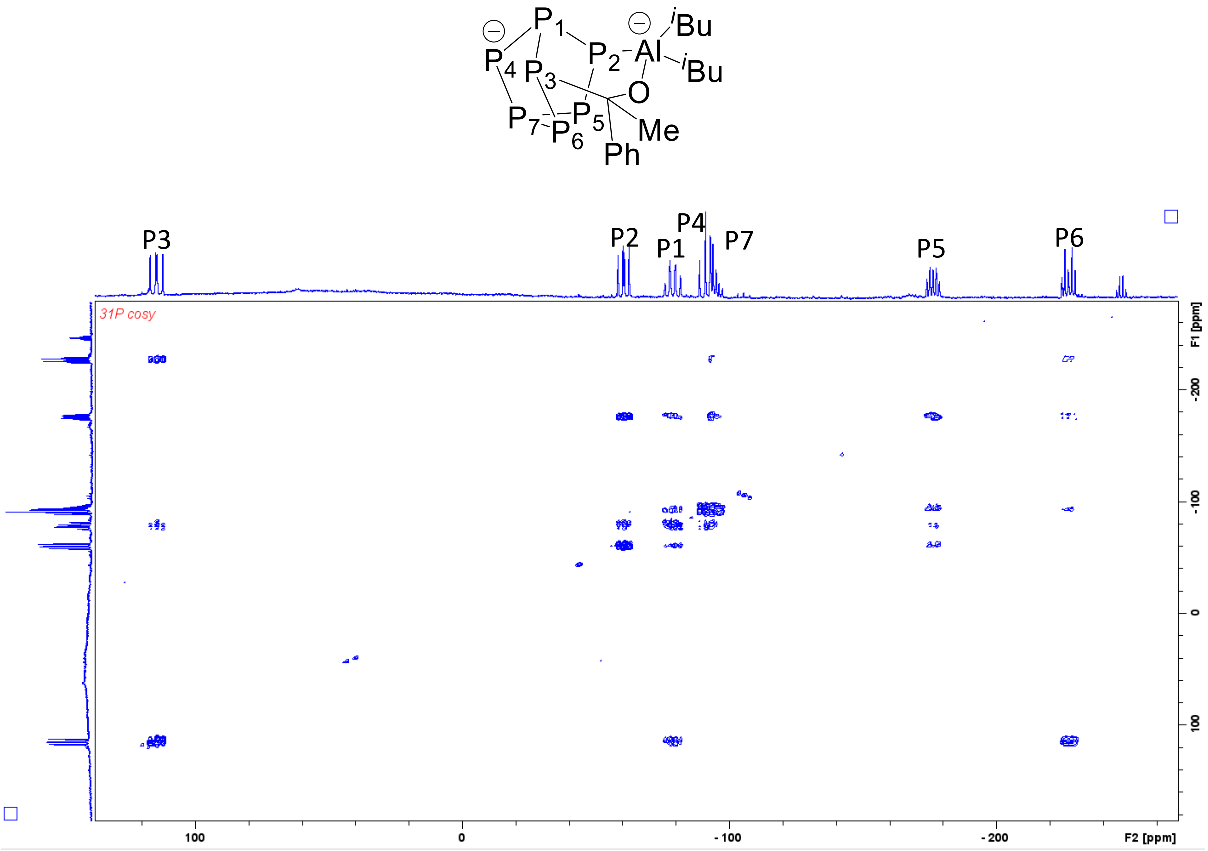

Figure S56. ^{1}H NMR spectrum (162 MHz, *o*DFB) of $[Na(18c6)]_2$[**2**] + 2 eq. PhCMe(=O).

4.1.6. Addition of Benzaldehyde to $[K(18c6)]_2$[3]

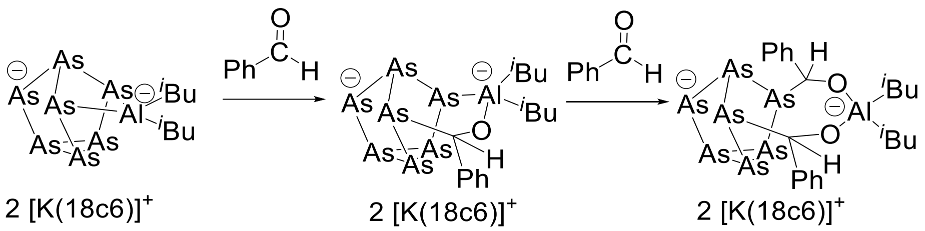

To a J Young NMR tube a solution of $[Na(18c6)]_2$[**3**] (25 mg, 0.020 mmol, 1 eq.) in *o*DFB and benzaldehyde (2.0 µL, 0.02 mmol, 1 eq.) was added and allowed to react for 30 min. The reaction was monitored by ^{1}H NMR spectroscopy. More benzaldehyde (2.0 µL, 0.02 mmol, +1 eq.) was added allowed to react for 30 min. Again the reaction was monitored by ^{1}H NMR spectroscopy. Finally, more benzaldehyde (4.0 µL, 0.04 mmol, +2 eq.) was added allowed to react for 30 min. The reaction was monitored by

^1H NMR spectroscopy. Note: In addition to the carbonyl inserted products, “free” benzaldehyde (δ= 9.98 ppm) can be observed after addition of 4 eq. benzaldehyde.

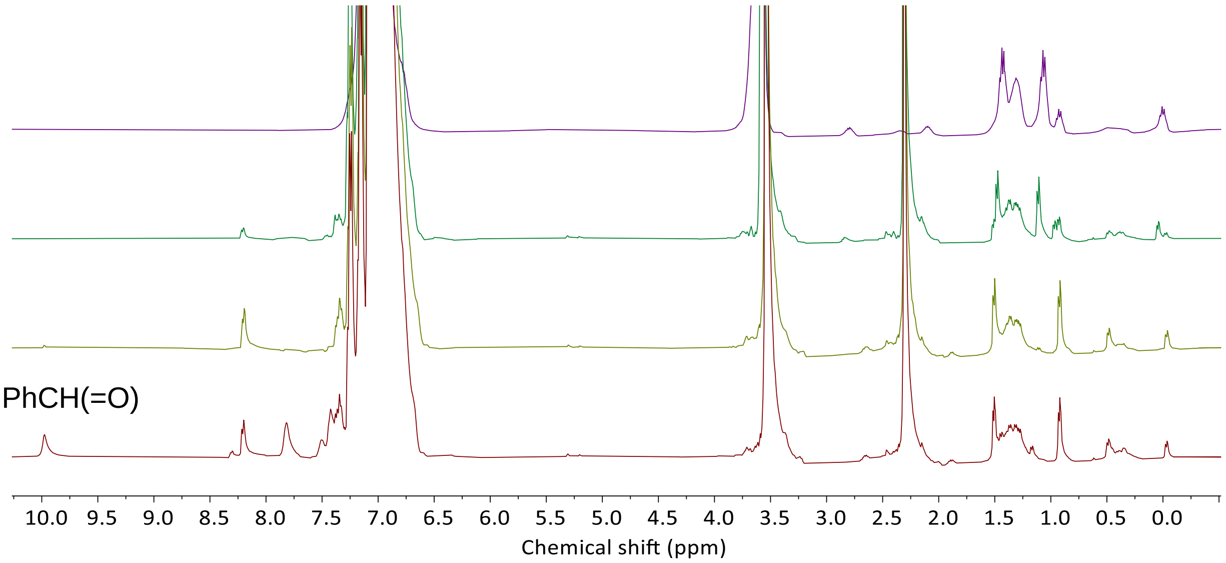

Figure S57. ^1H NMR spectrum (400 MHz, *o*DFB) of $[K(18c6)]_2$[**3**] + PhCH(=O). Top spectrum: $[K(18c6)]_2$[**3**]; second spectrum: $[K(18c6)]_2$[**3**] + 1 eq. PhCH(=O); third spectrum: $[K(18c6)]_2$[**3**] + 2 eq. PhCH(=O); bottom spectrum: $[K(18c6)]_2$[**3**] + 4 eq. PhCH(=O).

4.1.7. Addition of Acetophenone to $[K(18c6)]_2$[3]

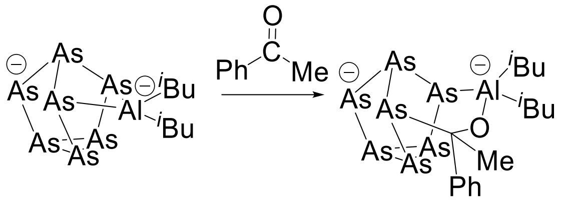

To a J Young NMR tube a solution of $[K(18c6)]_2$[**3**] (25 mg, 0.020 mmol, 1 eq.) in *o*DFB and acetophenone (2.3 µL, 0.020 mmol, 1 eq.) was added allowed to react for 24 h. The reaction was monitored by ^1H NMR spectroscopy, however which product is formed was not conclusive.

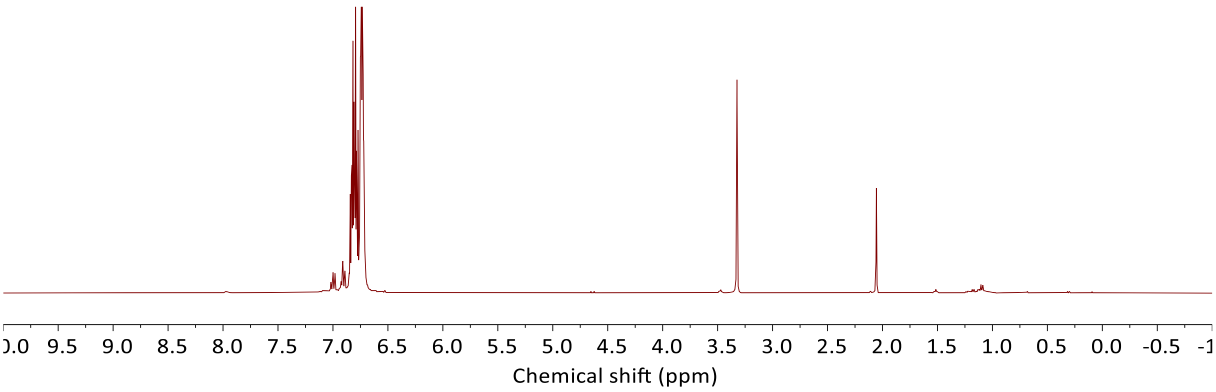

Figure S58. ^{1}H NMR spectrum (400 MHz, *o*DFB) of [Na(18c6)]$_2$[**3**] + 1 eq. PhCMe(=O). Note: the disappearance of the resonances associated for [Na(18c6)]$_2$[**3**].

4.1.8. Addition of Benzaldehyde to [Na(18c6)]$_2$[4]

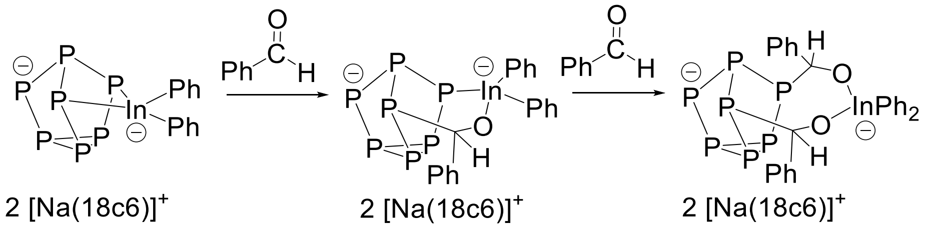

To a J Young NMR tube a solution of [Na(18c6)]$_2$[**4**] (15 mg, 0.014 mmol, 1 eq.) in *o*DFB and benzaldehyde (1.44 µL, 0.014 mmol, 1 eq.) was added and allowed to react for 30 min. The reaction was monitored by ^{1}H and ^{31}P NMR spectroscopy. More benzaldehyde (1.44 µL, 0.014 mmol, +1 eq.) was added and allowed to react for 24 h. again the reaction was monitored by ^{31}P NMR spectroscopy. Note: the [Na(18c6)]$_2$[**4**] salt is only partially soluble in *o*DFB preventing the observation of conclusive evidence of mono- and double-insertion (example ^{31}P NMR spectrum below).

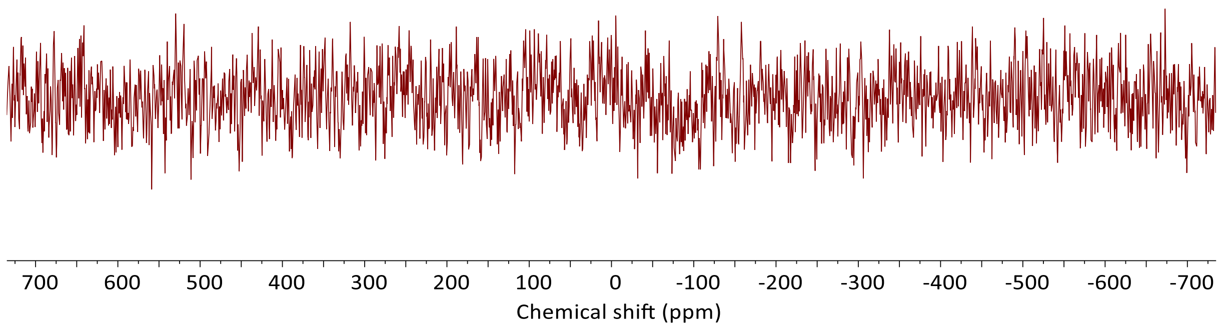

Figure S59. ^{31}P NMR spectrum (162 MHz, *o*DFB) of $[Na(18c6)]_2$[**4**] + 1 eq. PhCH(=O).

4.1.9. Addition of Acetophenone to $[Na(18c6)]_2$[4]

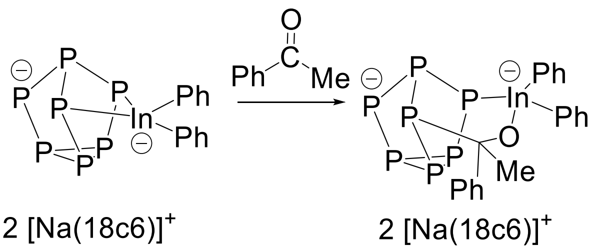

To a J Young NMR tube a solution of $[Na(18c6)]_2$[**4**] (25 mg, 0.024 mmol, 1 eq.) in *o*DFB and acetophenone (2.8 µL, 0.024 mmol, 1 eq.) was added and allowed to react for 30 min. The reaction was monitored by ^{1}H and ^{31}P NMR spectroscopy. More acetophenone (2.8 µL, 0.024 mmol, +1 eq.) was added and allowed to react for 24h. again the reaction was monitored by ^{31}P NMR spectroscopy. Note: the $[Na(18c6)]_2$[**4**] salt is only partially soluble in *o*DFB preventing the observation of conclusive evidence of mono- and double-insertion (example ^{31}P NMR spectrum below).

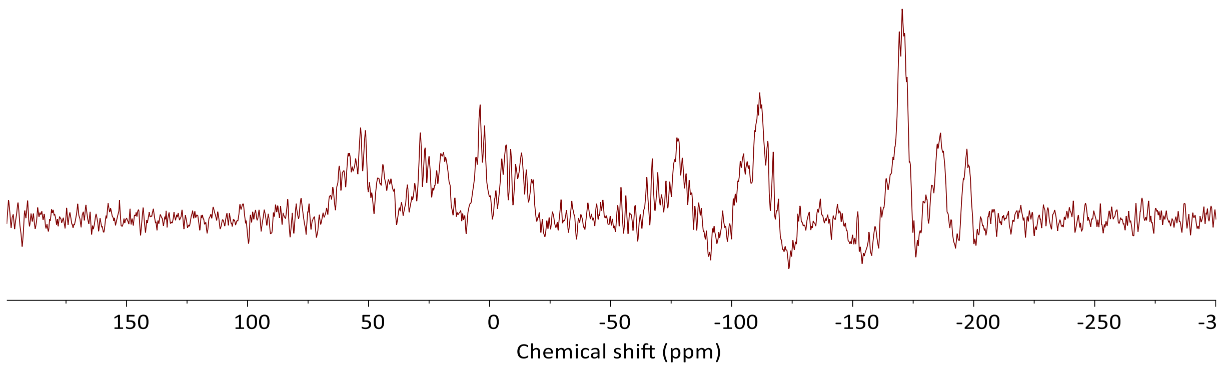

Figure S60. ^{31}P NMR spectrum (162 MHz, *o*DFB) of $[Na(18c6)]_2$[**4**] + 1 eq. PhCMe(=O).

4.2. Stoichiometric Hydroboration of Benzaldehyde Using [Na(18c6)]$_2$[1]

4.2.1. First Benzaldehyde Addition Followed by HBpin

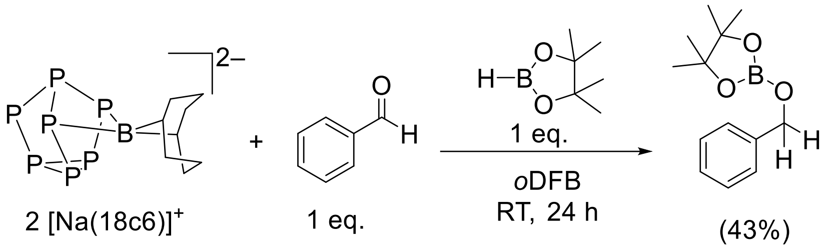

To a J Young NMR tube a solution of [Na(18c6)]$_2$[**1**] (15 mg, 0.016 mmol, 1.0 eq.) in *o*DFB and benzaldehyde (1.7 µL, 0.016 mmol, 1.0 eq.) was added and allowed to react for 1 h. Then, HBpin (2.4 µL, 0.016 mmol, 1.0 eq.) and toluene (25 µL, 0.24 mmol) were added to the reaction mixture and allowed to react for 24 h. The reaction was monitored by ^{1}H, ^{11}B, ^{11}B{^{1}H} and ^{31}P NMR spectroscopy. Using the toluene as an internal standard, the reaction gave an overall conversion of 43% conversion.

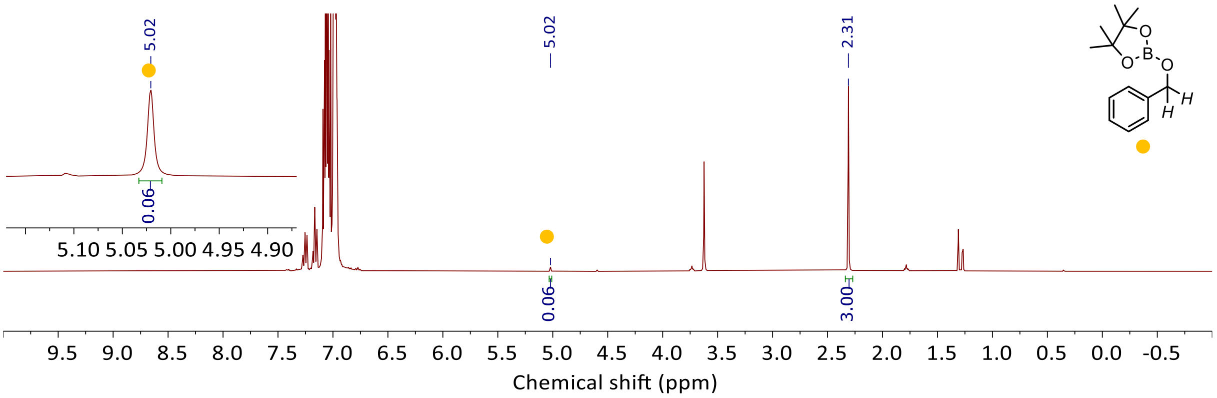

Figure S61. ^{1}H NMR spectrum (400 MHz, *o*DFB) stoichiometric reduction of PhCH(=O) using HBpin and [Na(18c6)]$_2$[**1**]. First addition of PhCH(=O).

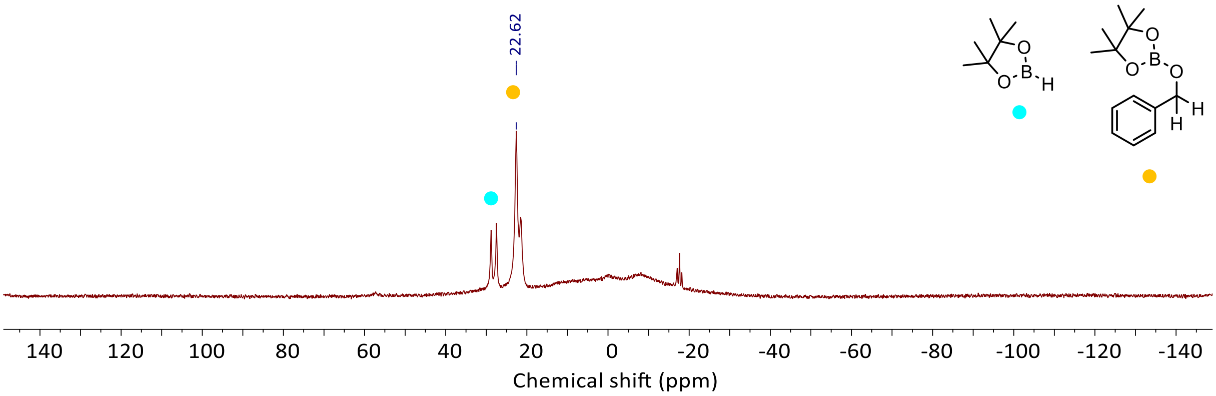

Figure S62. ^{11}B NMR spectrum (128 MHz, *o*DFB) stoichiometric reduction of PhCH(=O) using HBpin and $[Na(18c6)]_2$[**1**]. First addition of PhCH(=O).

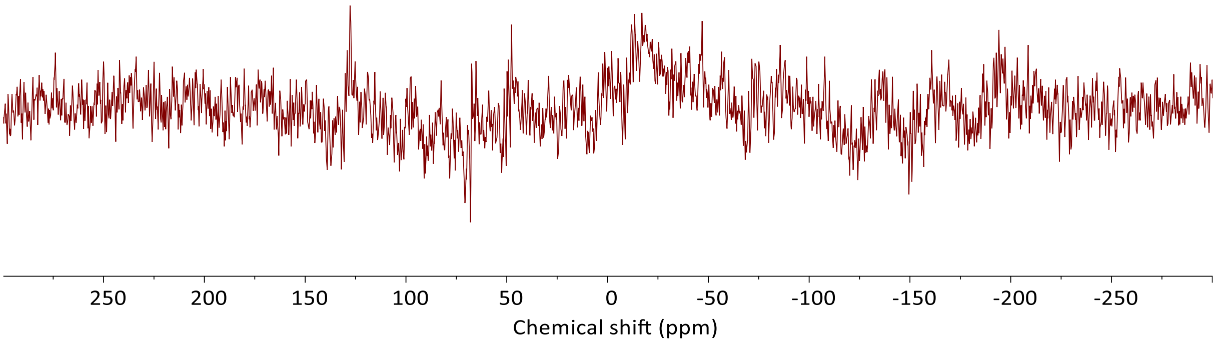

Figure S63. ^{31}P NMR spectrum (162 MHz, *o*DFB) stoichiometric reduction of PhCH(=O) using HBpin and $[Na(18c6)]_2$[**1**]. First addition of PhCH(=O).

4.2.2. First HBpin Addition Followed by Benzaldehyde

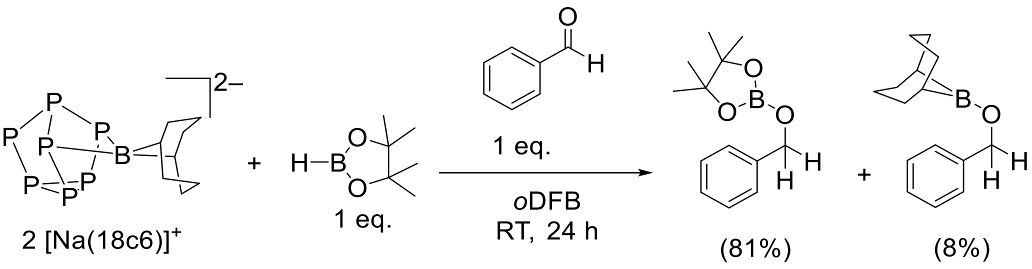

To a J Young NMR tube a solution of $[Na(18c6)]_2$[**1**] (15 mg, 0.016 mmol, 1.0 eq.) in *o*DFB and HBpin (2.4 µL, 0.016 mmol, 1.0 eq.) were added and allowed to react for 1 h. Then, benzaldehyde (1.7 µL, 0.016 mmol, 1.0 eq.) and toluene (25 µL, 0.24 mmol)

were added to the reaction mixture and allowed to react for 24 h. The reaction was monitored by ^{1}H, ^{11}B, $^{11}B\{^{1}H\}$ and ^{31}P NMR spectroscopy. Using the toluene as an internal standard, the reaction gave an overall conversion of 89% conversion with a 92:7 $PhCH_2OBpin$:$PhCH_2OBBN$ ratio.

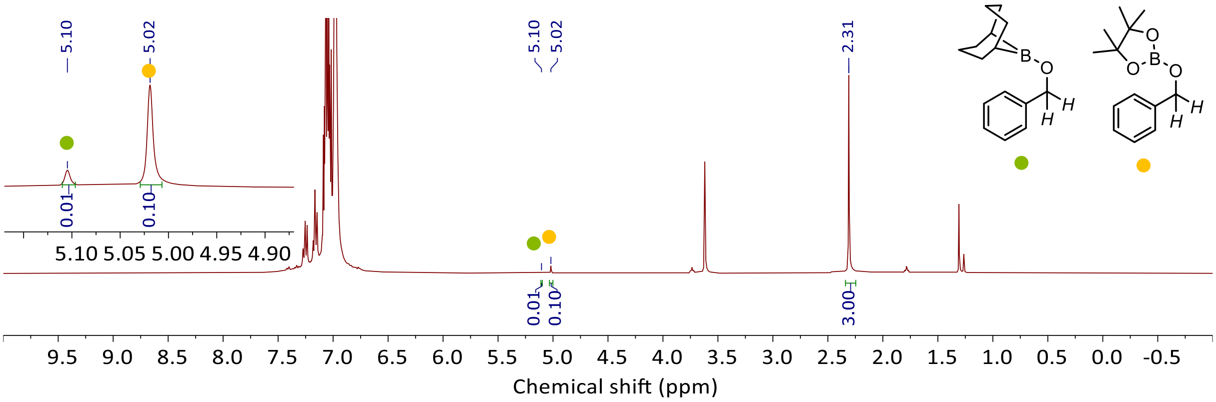

Figure S64. ^{1}H NMR spectrum (400 MHz, *o*DFB) stoichiometric reduction of PhCH(=O) using HBpin and $[Na(18c6)]_2$[**1**]. First addition of HBpin.

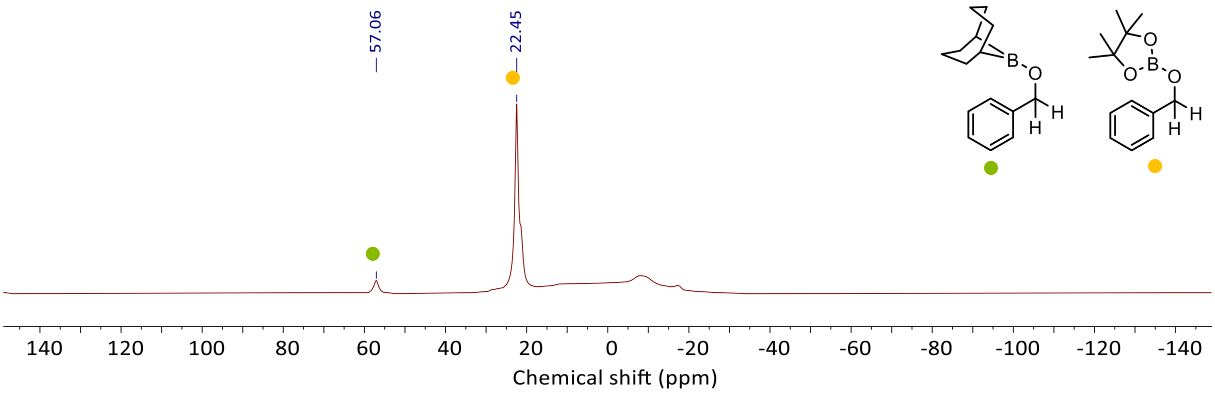

Figure S65. ^{11}B NMR spectrum (128 MHz, *o*DFB) stoichiometric reduction of PhCH(=O) using HBpin and $[Na(18c6)]_2$[**1**]. First addition of HBpin.

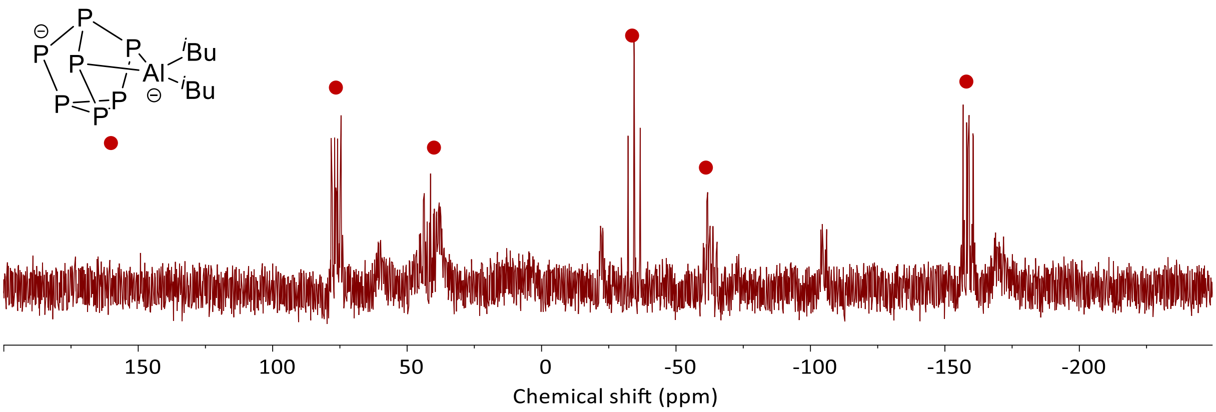

Figure S66. ^{31}P NMR spectrum (162 MHz, *o*DFB) stoichiometric reduction of PhCH(=O) using HBpin and $[Na(18c6)]_2$[**1**]. First addition of HBpin.

4.3. Stoichiometric Hydroboration of Benzaldehyde Using $[Na(18c6)]_2$[2]

4.3.1. First Benzaldehyde Addition Followed by HBpin

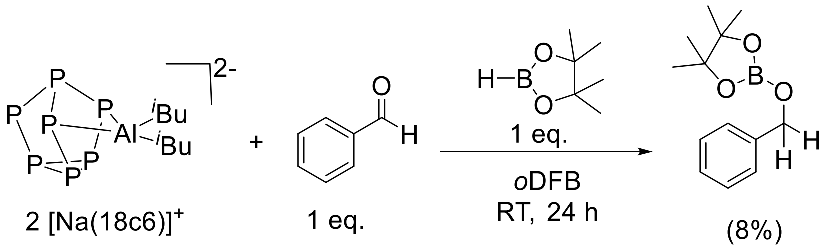

To a J Young NMR tube a solution of $[Na(18c6)]_2$[**2**] (25 mg, 0.027 mmol, 1.0 eq.) in *o*DFB and benzaldehyde (2.8 µL, 0.027 mmol, 1.0 eq.) was added and allowed to react for 1 h. Then, HBpin (3.9 µL, 0.027 mmol, 1.0 eq.) and toluene (30 µL, 0.28 mmol) were added to the reaction mixture and allowed to react for 24 h. The reaction was monitored by ^{1}H, ^{11}B, $^{11}B\{^{1}H\}$ and ^{31}P NMR spectroscopy. Using the toluene as an internal standard, the reaction gave an overall conversion of 8% conversion.

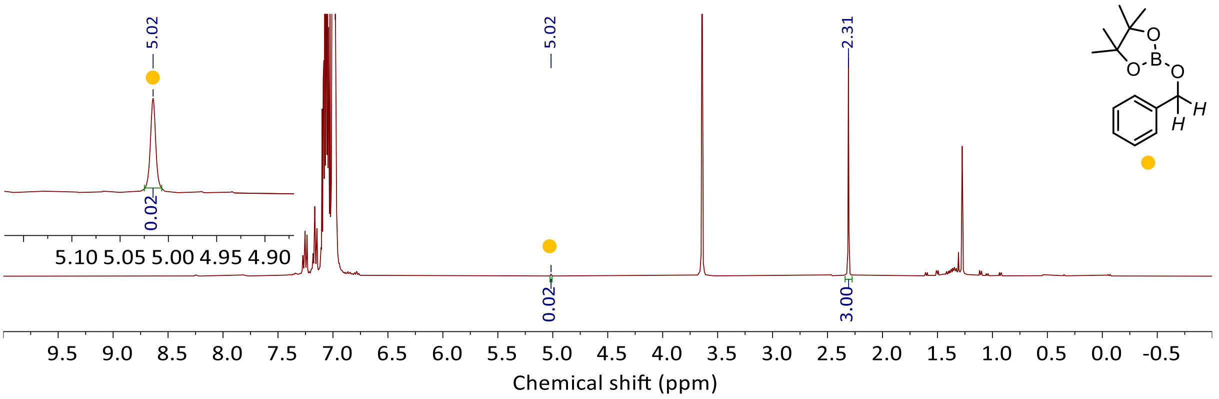

Figure S67. ^{1}H NMR spectrum (400 MHz, *o*DFB) stoichiometric reduction of PhCH(=O) using HBpin and [Na(18c6)]$_2$[**2**]. First addition of PhCH(=O).

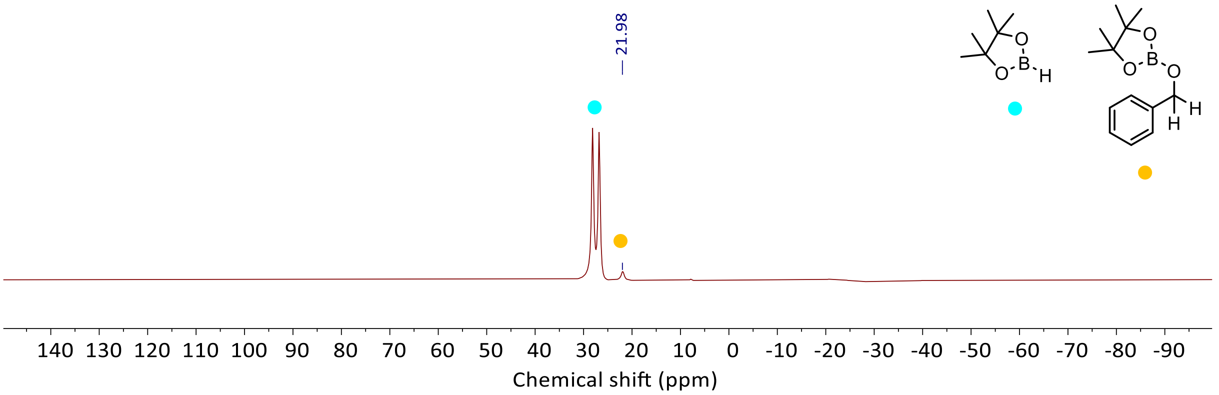

Figure S68. ^{11}B NMR spectrum (128 MHz, *o*DFB) stoichiometric reduction of PhCH(=O) using HBpin and [Na(18c6)]$_2$[**2**]. First addition of PhCH(=O).

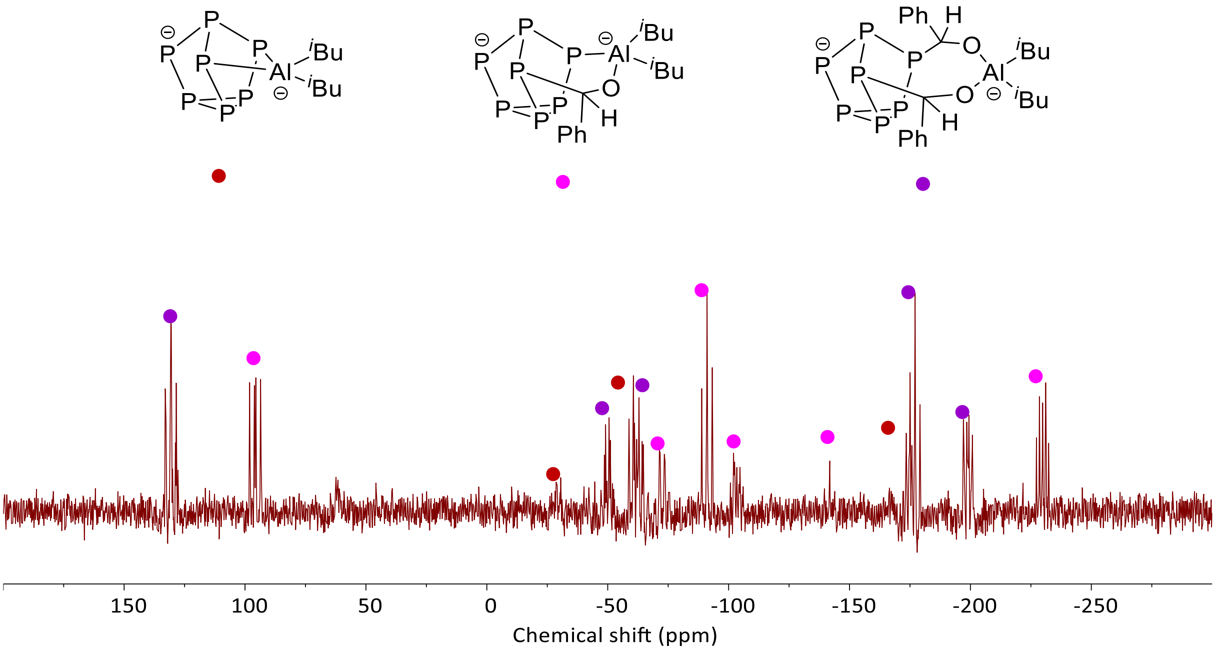

Figure S69. ^{31}P NMR spectrum (162 MHz, *o*DFB) stoichiometric reduction of PhCH(=O) using HBpin and [Na(18c6)]$_2$[**2**]. First addition of PhCH(=O).

4.3.2. First HBpin Addition Followed by Benzaldehyde

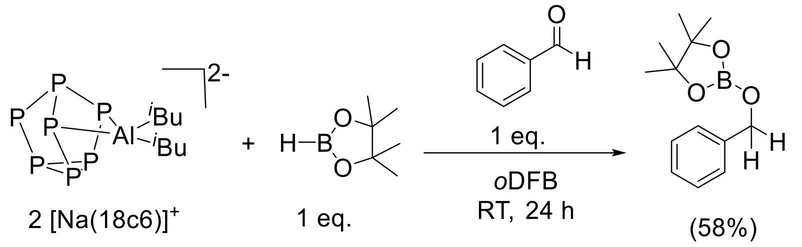

To a J Young NMR tube a solution of [Na(18c6)]$_2$[**2**] (25 mg, 0.027 mmol, 1.0 eq.) in *o*DFB and HBpin (3.9 µL, 0.027 mmol, 1.0 eq.) were added and allowed to react for 1 h. Then, benzaldehyde (2.8 µL, 0.027 mmol, 1.0 eq.) and toluene (30 µL, 0.28 mmol) were added to the reaction mixture and allowed to react for 24 h. The reaction was monitored by ^{1}H, ^{11}B, ^{11}B{^{1}H} and ^{31}P NMR spectroscopy. Using the toluene as an internal standard, the reaction gave an overall conversion of 58% conversion.

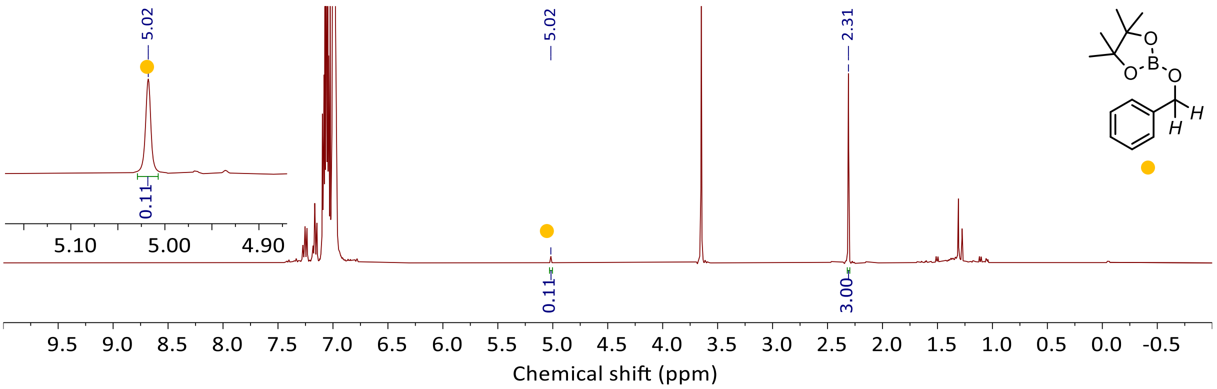

Figure S70. ^{1}H NMR spectrum (400 MHz, *o*DFB) stoichiometric reduction of PhCH(=O) using HBpin and [Na(18c6)]$_2$[**2**]. First addition of HBpin.

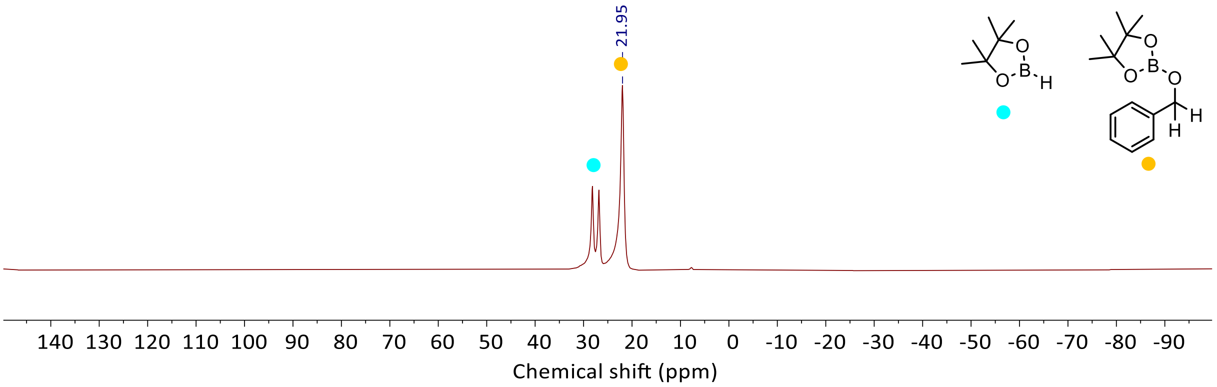

Figure S71. ^{11}B NMR spectrum (128 MHz, *o*DFB) stoichiometric reduction of PhCH(=O) using HBpin and [Na(18c6)]$_2$[**2**]. First addition of HBpin.

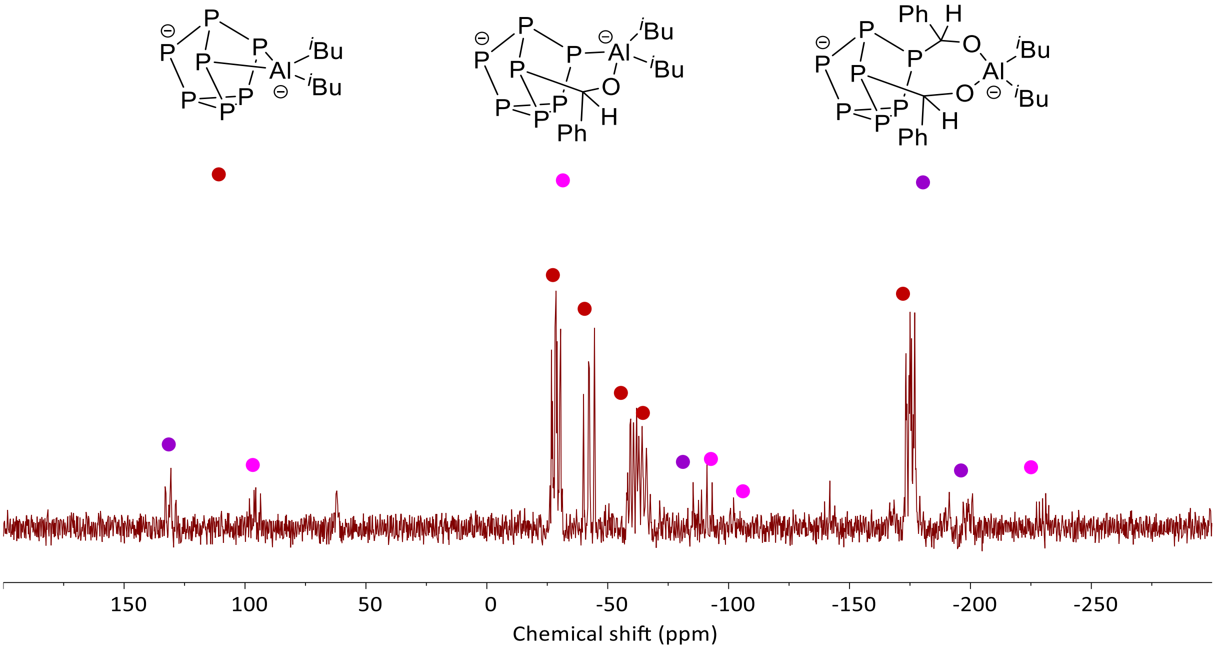

Figure S72. ^{31}P NMR spectrum (162 MHz, *o*DFB) stoichiometric reduction of PhCH(=O) using HBpin and $[Na(18c6)]_2$[**2**]. First addition of HBpin.

4.4. Stoichiometric Hydroboration of Benzaldehyde Using $[K(18c6)]_2$[3]

4.4.1. First Benzaldehyde Addition Followed by HBpin

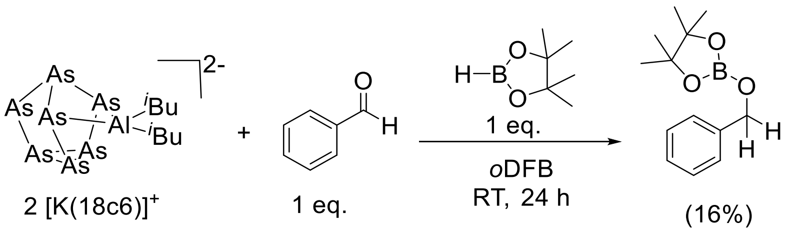

To a J Young NMR tube a solution of $[Na(18c6)]_2$[**2**] (25 mg, 0.02 mmol, 1 eq.) in *o*DFB and benzaldehyde (2.0 µL, 0.02 mmol, 1 eq.) was added and allowed to react for 1 h. Then, HBpin (2.8 µL, 0.02 mmol, 1 eq.) and toluene (25 µL, 0.24 mmol) were added to the reaction mixture and allowed to react for 24 h. The reaction was monitored by ^{1}H and ^{11}B NMR spectroscopy. Using the toluene as an internal standard, the reaction gave an overall conversion of 16% conversion.

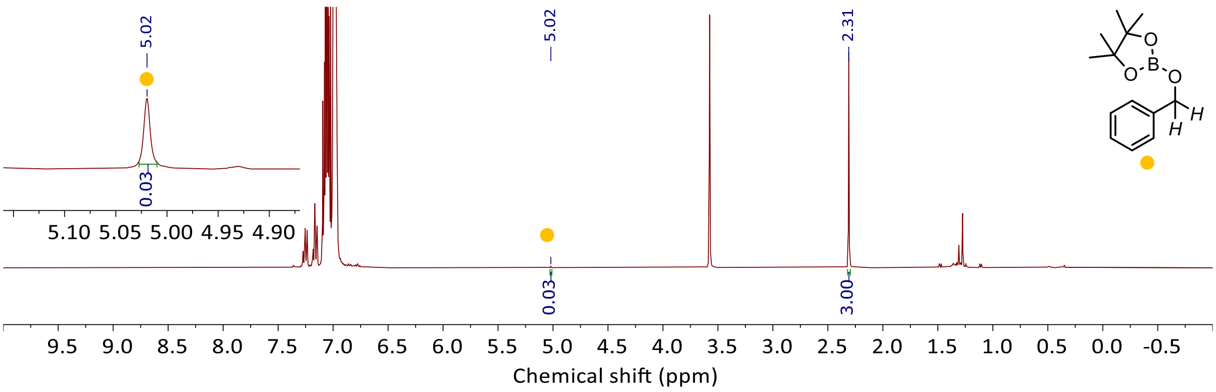

Figure S73. ^{1}H NMR spectrum (400 MHz, *o*DFB) stoichiometric reduction of PhCH(=O) using HBpin and $[K(18c6)]_2$[**3**]. First addition of PhCH(=O).

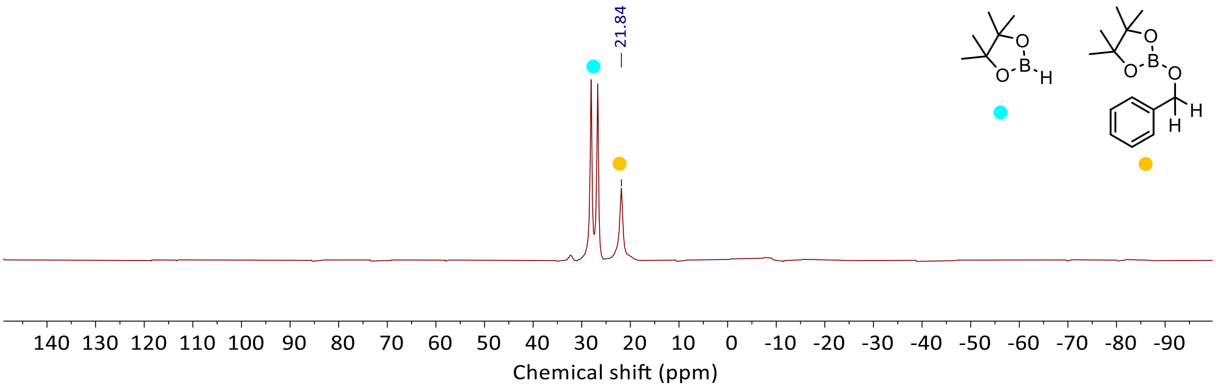

Figure S74. ^{11}B NMR spectrum (128 MHz, *o*DFB) stoichiometric reduction of PhCH(=O) using HBpin and $[K(18c6)]_2$[**3**]. First addition of PhCH(=O).

4.4.2. First HBpin Addition Followed by Benzaldehyde

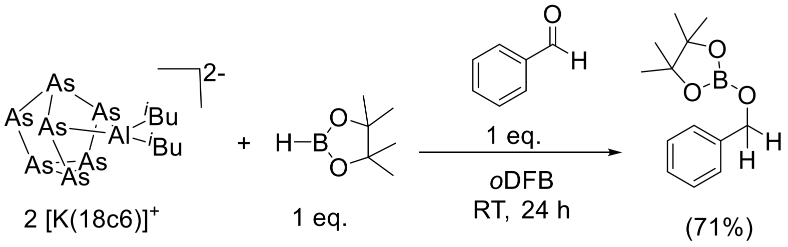

To a J Young NMR tube a solution of $[Na(18c6)]_2$[**3**] (25 mg, 0.020 mmol, 1.0 eq.) in *o*DFB and HBpin (2.8 µL, 0.020 mmol, 1.0 eq.) was added and allowed to react for 1 h. Then, benzaldehyde (2.0 µL, 0.020 mmol, 1.0 eq.) and toluene (25 µL, 0.24 mmol) were added to the reaction mixture and allowed to react for 24 h. The reaction was monitored by ^{1}H and ^{11}B NMR spectroscopy. Using the toluene as an internal standard, the reaction gave an overall conversion of 71% conversion.

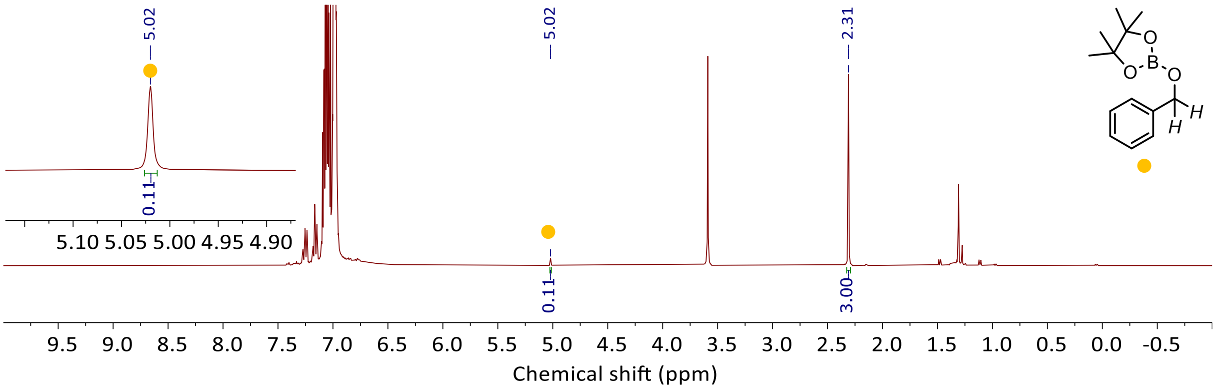

Figure S75. ^{1}H NMR spectrum (400 MHz, *o*DFB) stoichiometric reduction of PhCH(=O) using HBpin and [K(18c6)]$_2$[**3**]. First addition of HBpin.

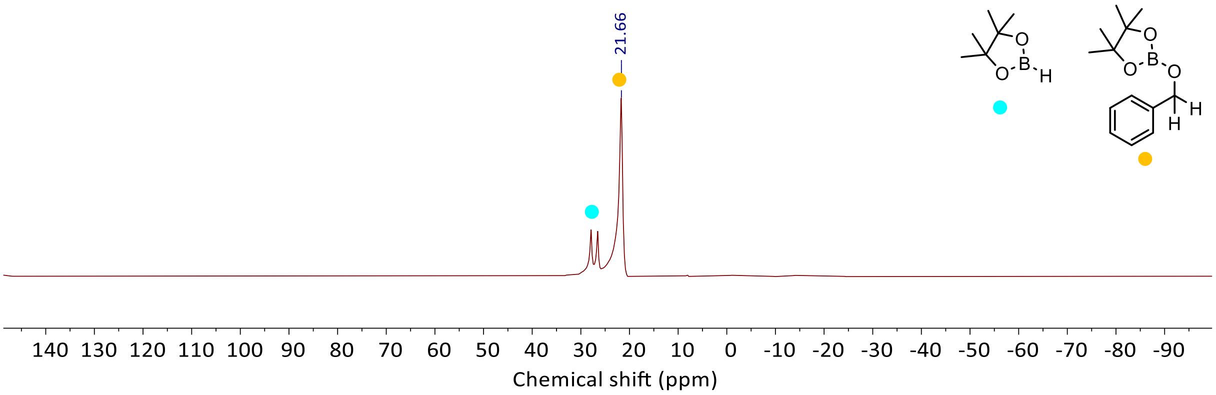

Figure S76. ^{11}B NMR spectrum (128 MHz, *o*DFB) stoichiometric reduction of PhCH(=O) using HBpin and [K(18c6)]$_2$[**3**]. First addition of HBpin.

4.5. Stoichiometric Hydroboration of Benzaldehyde Using [K(18c6)]$_2$[3]

4.5.1. First Benzaldehyde Addition Followed by HBpin

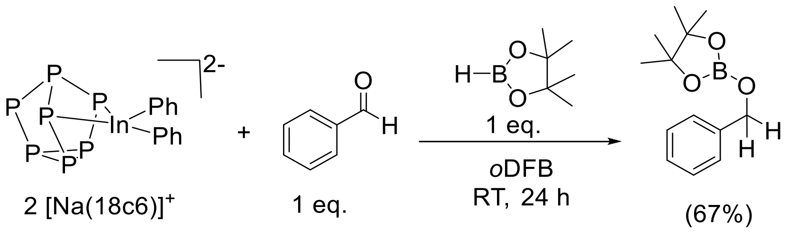

To a J Young NMR tube a solution of [Na(18c6)]$_2$[**4**] (15 mg, 0.014 mmol, 1 eq.) in *o*DFB and benzaldehyde (1.44 µL, 0.014 mmol, 1 eq.) were added and allowed to react for 1 h. Then, HBpin (2.0 µL, 0.014 mmol, 1 eq.) and toluene (25 µL, 0.24 mmol)

were added to the reaction mixture and allowed to react for 24 h. The reaction was monitored by ^{1}H and ^{11}B NMR spectroscopy. Using the toluene as an internal standard, the reaction gave an overall conversion of 67% conversion.

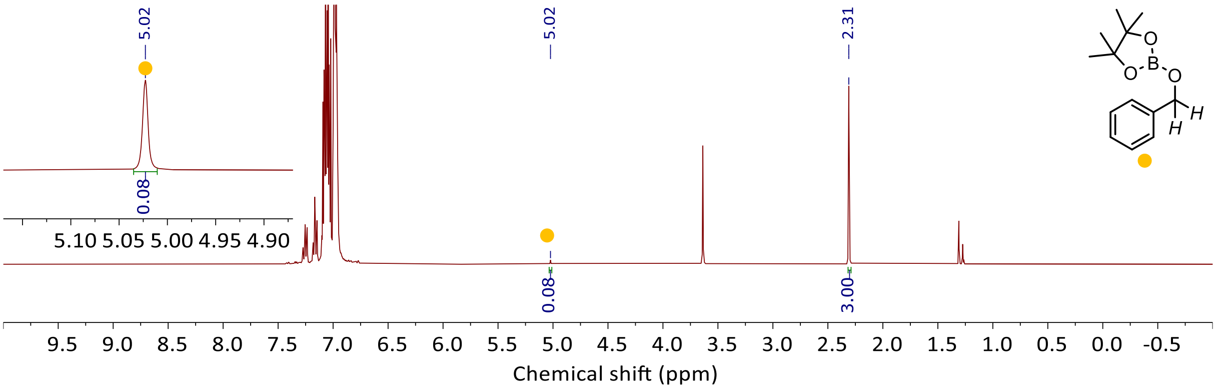

Figure S77. ^{1}H NMR spectrum (400 MHz, *o*DFB) stoichiometric reduction of PhCH(=O) using HBpin and $[Na(18c6)]_2$[**4**]. First addition of PhCH(=O).

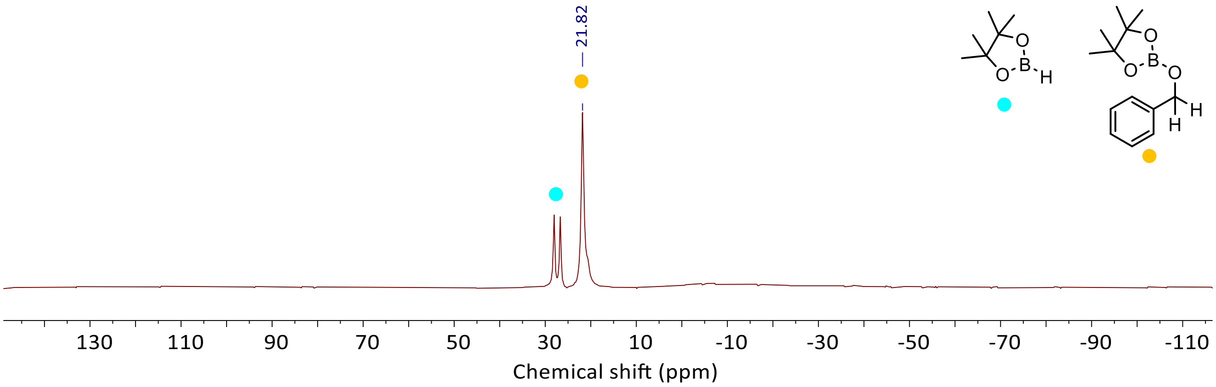

Figure S78. ^{11}B NMR spectrum (128 MHz, *o*DFB) stoichiometric reduction of PhCH(=O) using HBpin and $[Na(18c6)]_2$[**4**]. First addition of PhCH(=O).

4.5.2. First HBpin addition followed by benzaldehyde

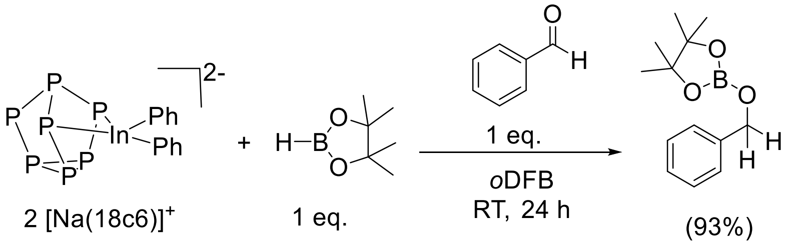

To a J Young NMR tube a solution of [Na(18c6)]$_2$[**4**] (15 mg, 0.014 mmol, 1.0 eq.) in *o*DFB and HBpin (2.0 µL, 0.014 mmol, 1.0 eq.) were added and allowed to react for 1 h. Then, benzaldehyde (1.44 mL, 0.014 mmol, 1.0 eq.) and toluene (25 µL, 0.24 mmol) were added to the reaction mixture and allowed to react for 24 h. The reaction was monitored by ^{1}H and ^{11}B NMR spectroscopy. Using the toluene as an internal standard, the reaction gave an overall conversion of 93% conversion.

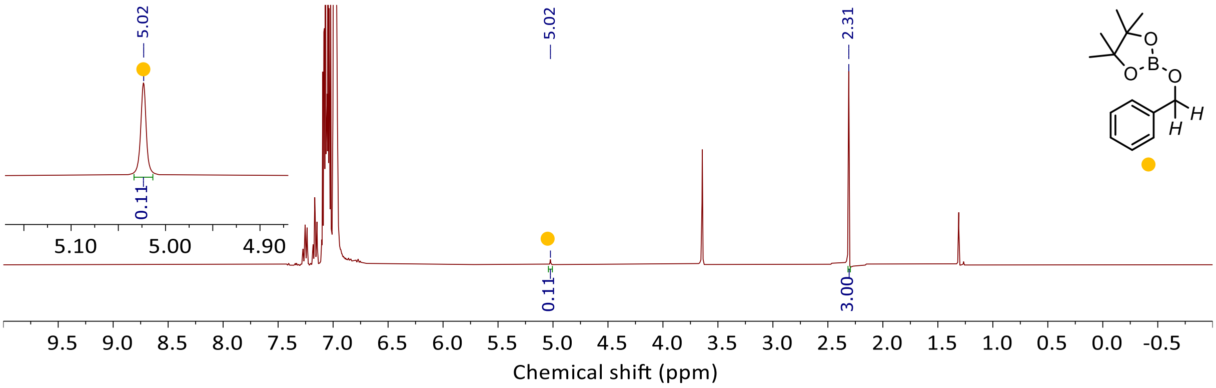

Figure S79. ^{1}H NMR spectrum (400 MHz, *o*DFB) stoichiometric reduction of PhCH(=O) using HBpin and [Na(18c6)]$_2$[**4**]. First addition of HBpin.

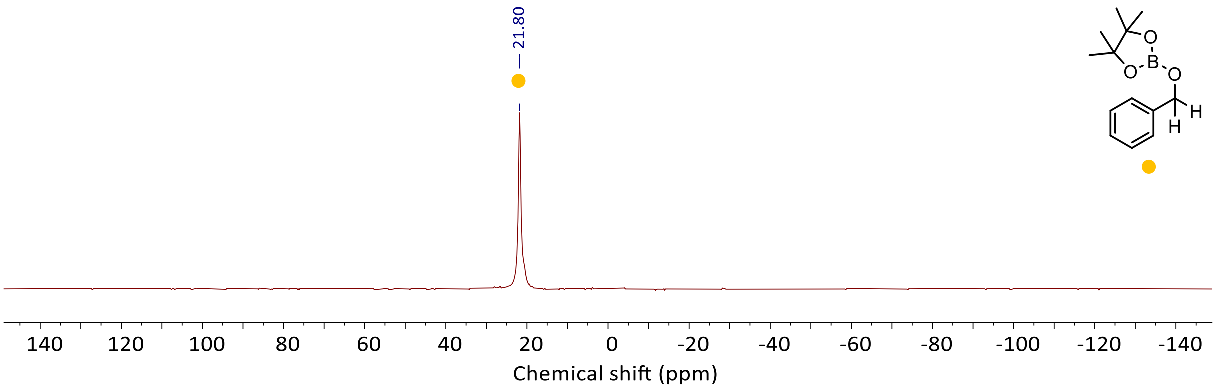

Figure S80. ^{11}B NMR spectrum (128 MHz, *o*DFB) stoichiometric reduction of PhCH(=O) using HBpin and [Na(18c6)]$_2$[**4**]. First addition of HBpin.

5. Variable Time Normalization Analysis

Benzaldehyde was used as a model substrate to investigate the mechanism of the CO_2 hydroboration. Mimicking conditions used in the hydroboration of CO_2, variable time normalization analysis was applied to elaborate the order of the reaction.[28, 29] A J Young NMR tube was charged with a solution of [Na(18c6)][**X**] (**X** = **1**, **2**) in *o*DFB. To this solution was added internal standard, borane and benzaldehyde. The total volume of the reaction mixtures was 0.6 mL (protic solvent). Reaction profiles were tracked by performing the reaction in an NMR spectrometer (a Bruker 400 AVIII spectrometer) until ~70% conversion was achieved (~40% conversion for two experiments). Samples were loaded into the NMR spectrometer within 2-2.5 min, exact time was measured. While transferring the sample to the NMR spectrometer it was found that shaking the mixture irregularly leads to mixed results with more shaking resulting in faster conversion during the transfer, therefore the sample was only turned-upside-down once. The NMR spectrometer was lock, tuned and shimmed onto a sample of benzaldehyde in C_6D_6 prior to analysis due to the protic nature of the solvent used in the catalysis. Receiver gain parameter was set to 10 and line broadening was set to 0.3 Hz as balance between signal-to-noise and preventing overloading the FID digitizer due to the protic solvent resonances.

5.1. Order in Reagents of the Hydroboration of Benzaldehyde Using HBBN dimer

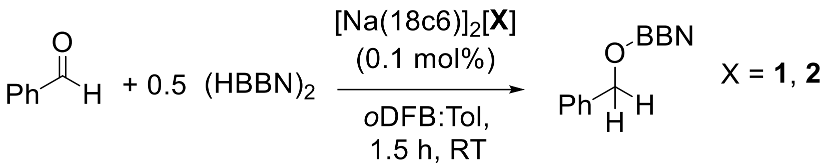

Concentrations of the product (^{1}H δ = 5.04 ppm), $(HBBN)_2$ (^{1}H δ = 1.81–1.67 ppm), and benzaldehyde (^{1}H δ = 9.84 ppm) were calculated by integration of the ^{1}H NMR spectrum using C_6Me_6 as an internal standard (^{1}H δ = 2.20 ppm). Following the variable time normalization analysis as described by Burés,[28, 29] the reaction order can be obtained via a graphical representation. The analysis supports a fitting of a zero order in the concentration of aldehyde and catalyst and a half order in the concentration of BBN dimer, represented in Formula 1. This suggests that the rate limiting step is a reaction including $(HBBN)_2$ but not the catalysts, consistent with breaking of the $(HBBN)_2$ dimer be rate limiting.

Formula 1. $[\mathrm{P}] = \mathrm{k} \int [\mathrm{Benzaldehyde}]^0 \times [\mathrm{BBN\ dimer}]^{0.5} \times [\mathrm{Catalyst}]^0 \ \mathrm{dt}$

Table S4. VTNA experiment hydroboration of benzaldehyde using HBBN dimer.

Exp.	Added Benzaldehyde (mmol) (A)	Added $(HBBN)_2$ (mmol) (B)	Added Catalyst (mmol) (Cat)
1 (▲)	0.22	0.11	0.00022
2 (●)	0.11	0.11	0.00022
3 (■)	0.22	0.06	0.00022
4 (◆)	0.22	0.1	0.00033

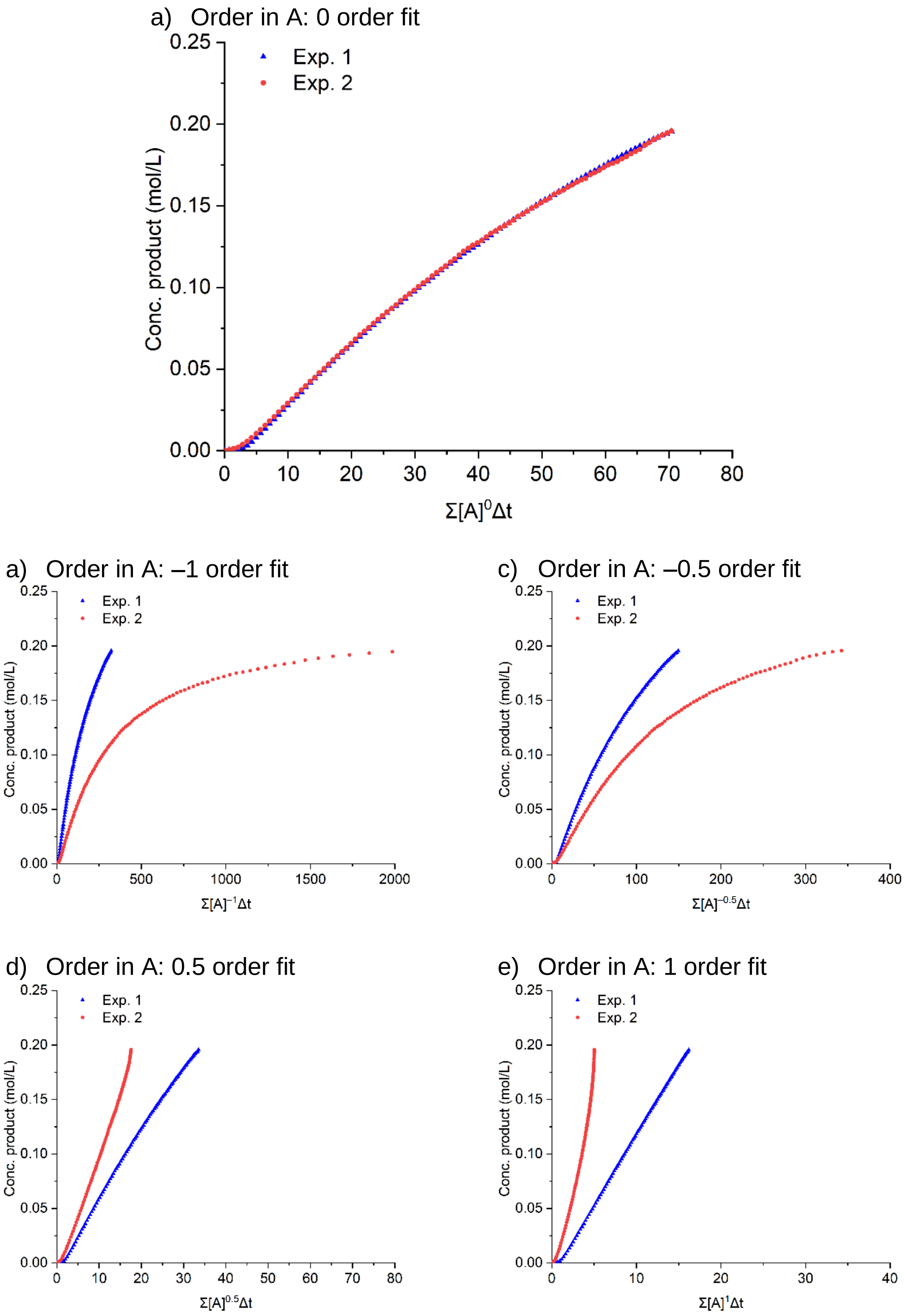

Figure S81. VTNA hydroboration benzaldehyde using HBBN dimer as reducing agent and 0.1 mol% $[Na(18c6)]_2$[**1**] as catalyst. Graphs a, b, c, d, and e are the graphical representation of different orders in concentration benzaldehyde ([A]) using the concentration of the product, obtained from the analysis.

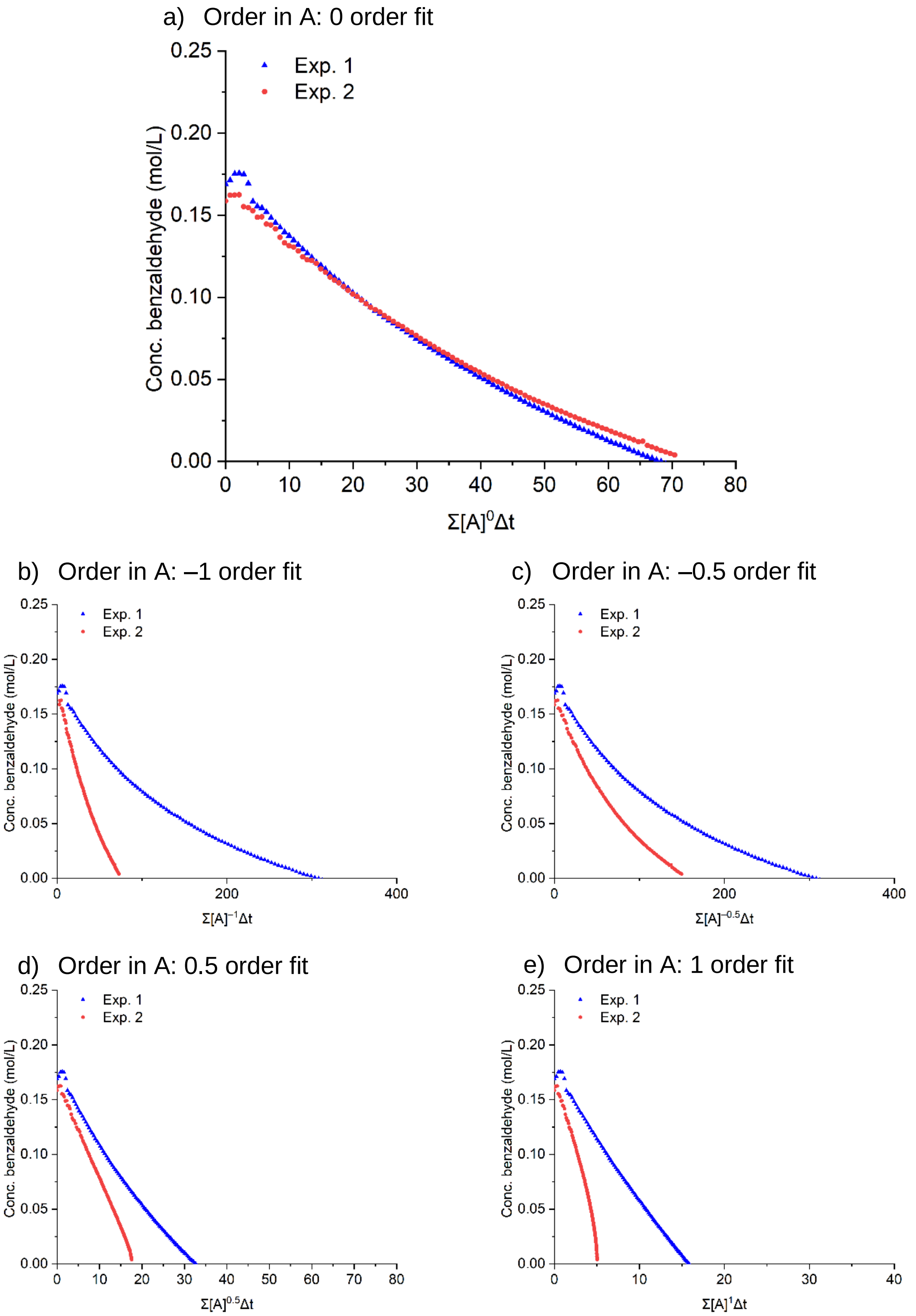

Figure S82. VTNA hydroboration benzaldehyde using HBBN dimer as reducing agent and 0.1 mol% $[Na(18c6)]_2$[**1**] as catalyst. Graphs a, b, c, d, and e are the graphical representation of different orders in concentration benzaldehyde ([A]) using the concentration of benzaldehyde ([A]), obtained from the analysis.

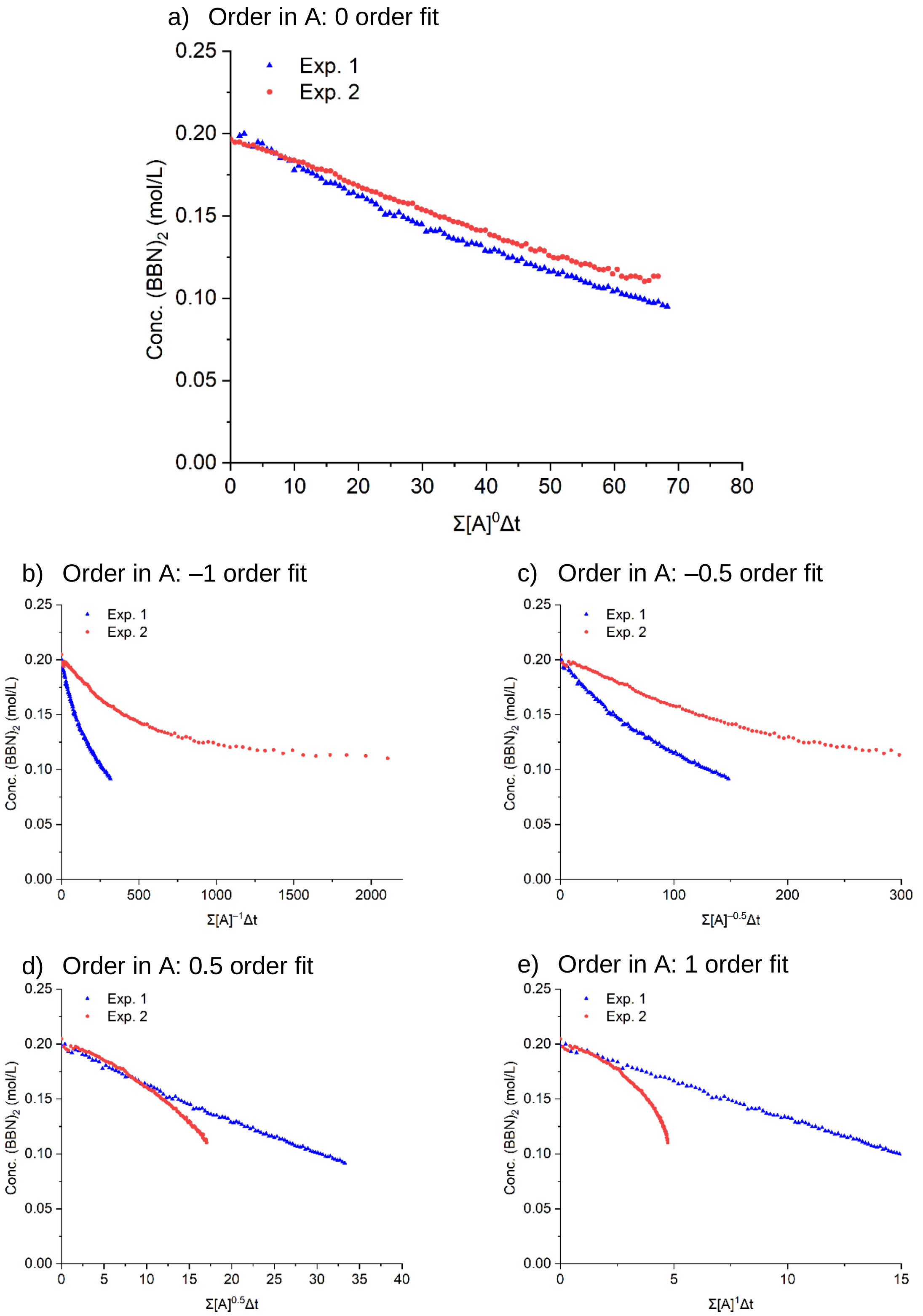

Figure S83. VTNA hydroboration benzaldehyde using HBBN dimer as reducing agent and 0.1 mol% $[Na(18c6)]_2$[**1**] as catalyst. Graphs a, b, c, d, and e are the graphical representation of different orders in concentration benzaldehyde ([A]) using the concentration of HBBN dimer ([B]), obtained from the analysis.

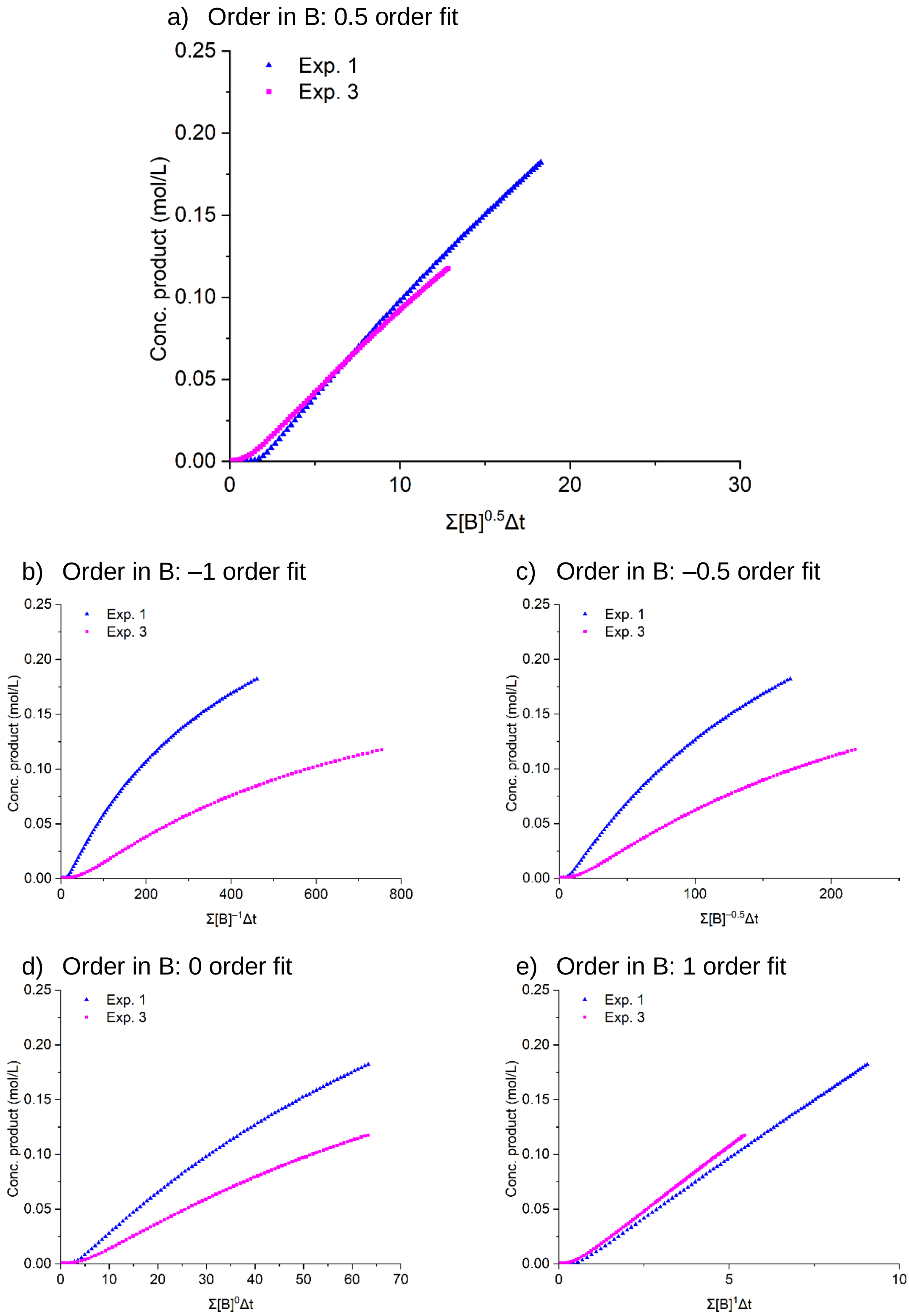

Figure S84. VTNA hydroboration benzaldehyde using HBBN dimer as reducing agent and 0.1 mol% [Na(18c6)]$_2$[**1**] as catalyst. Graphs a, b, c, d, and e are the graphical representation of different orders in HBBN dimer catalyst ([B]) using the concentration of the product, obtained from the analysis.

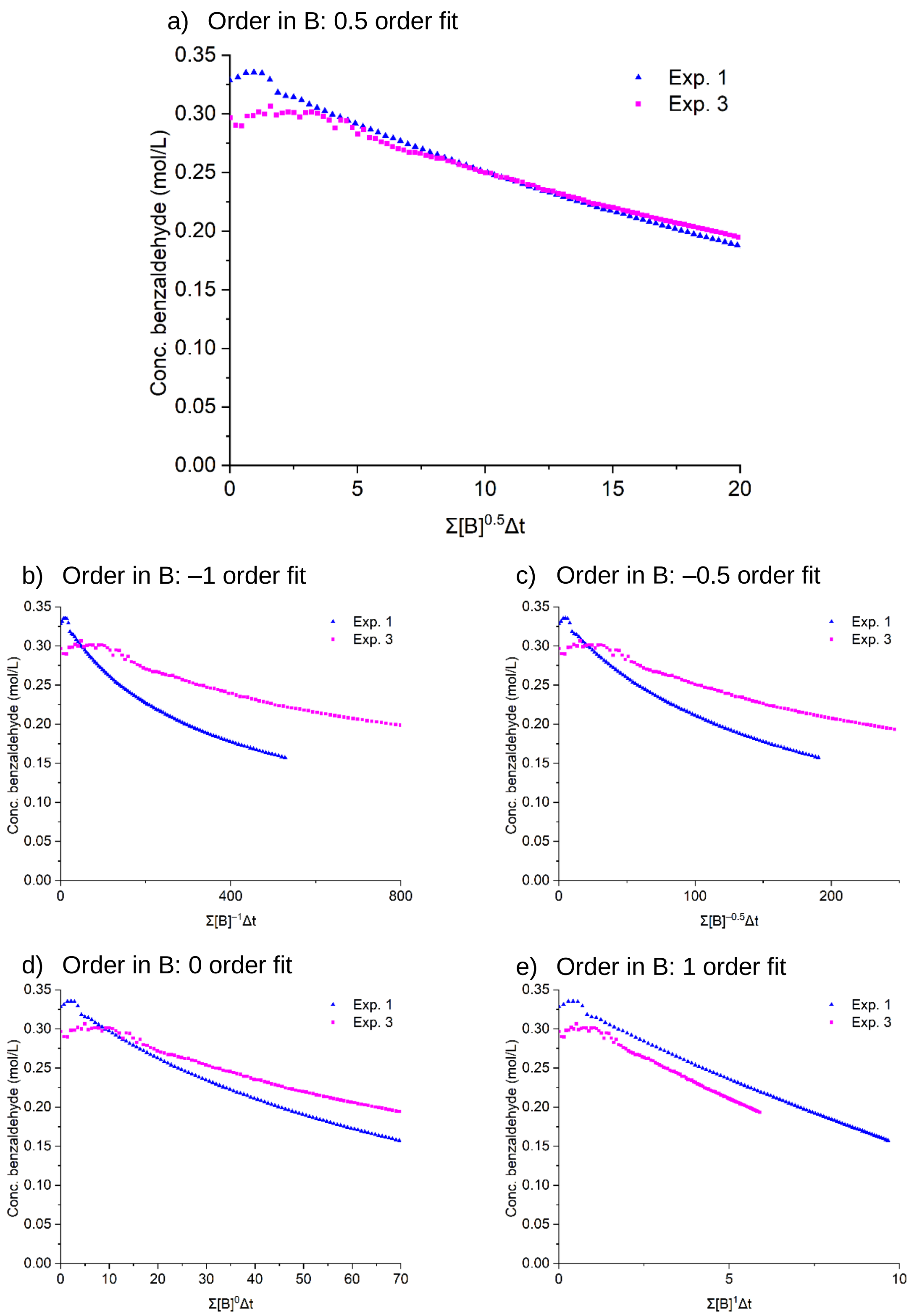

Figure S85. VTNA hydroboration benzaldehyde using HBBN dimer as reducing agent and 0.1 mol% $[Na(18c6)]_2$[**1**] as catalyst. Graphs a, b, c, d, and e are the graphical representation of different orders in HBBN dimer catalyst ([B]) using the concentration of benzaldehyde ([A]), obtained from the analysis.

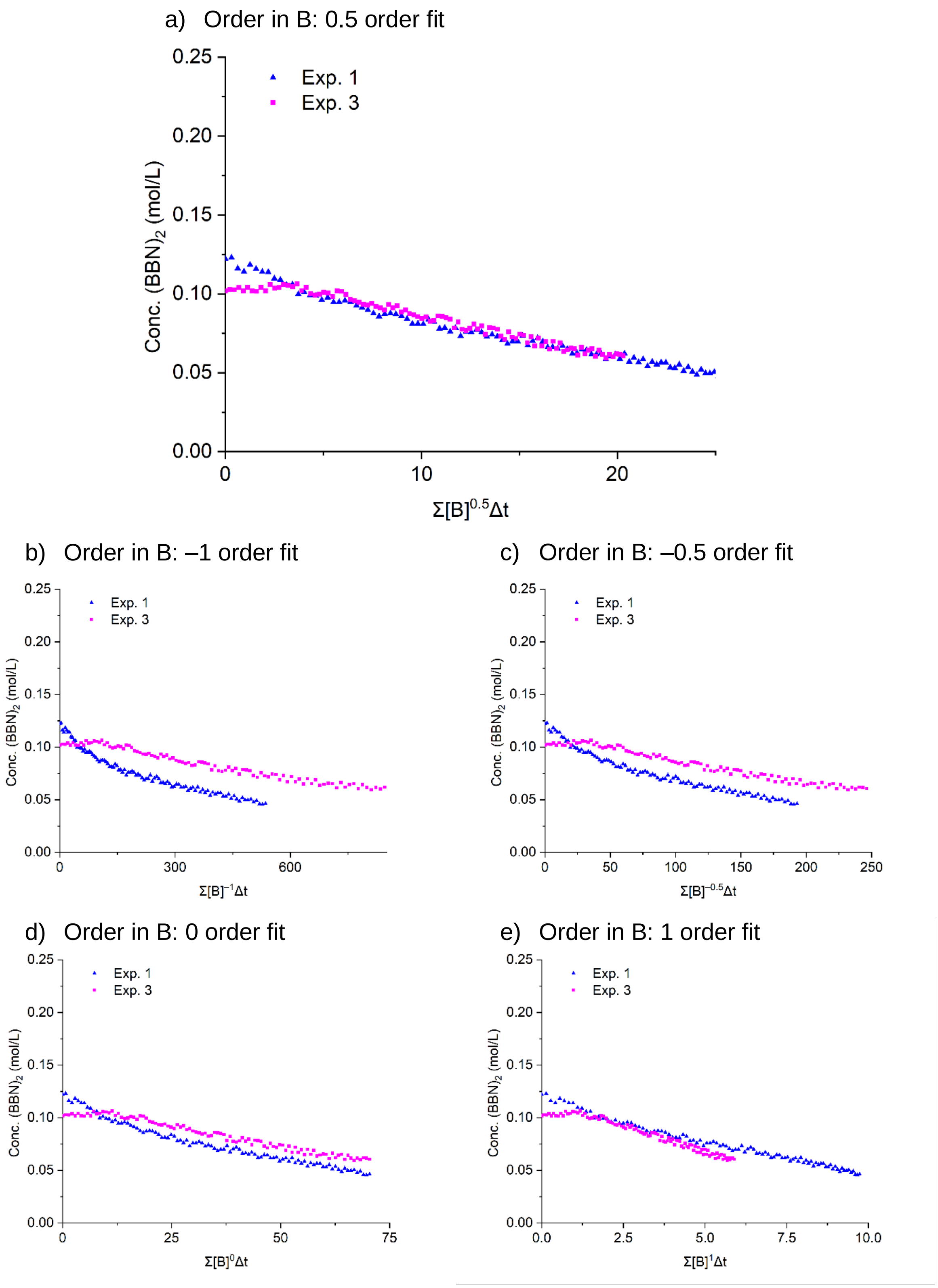

Figure S86. VTNA hydroboration benzaldehyde using HBBN dimer as reducing agent and 0.1 mol% $[Na(18c6)]_2$[**1**] as catalyst. Graphs a, b, c, d, and e are the graphical representation of different orders in HBBN dimer catalyst ([B]) using the concentration of HBBN dimer ([B]), obtained from the analysis.

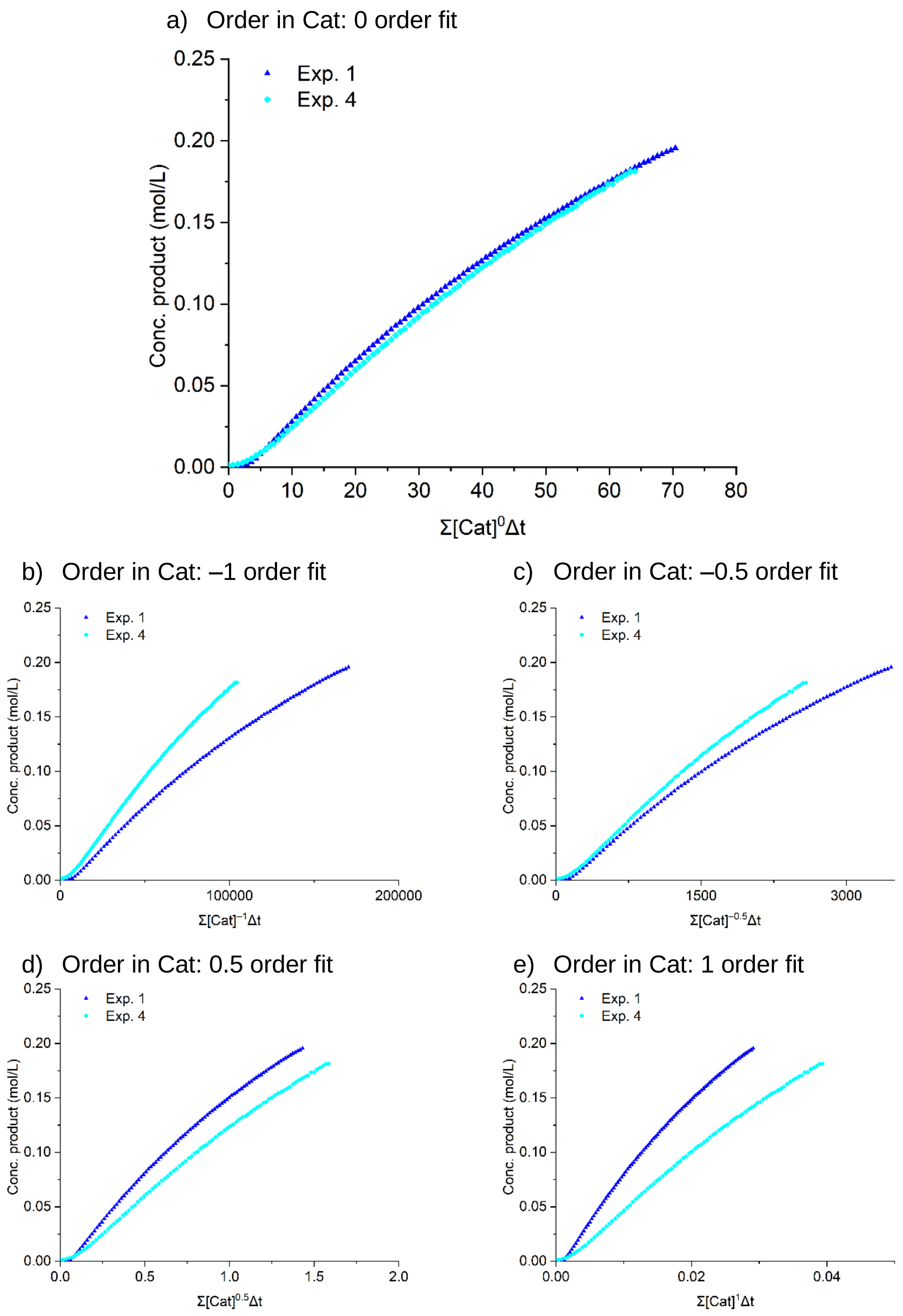

Figure S87. VTNA hydroboration benzaldehyde using HBBN dimer as reducing agent and 0.1 mol% $[Na(18c6)]_2$[**1**] as catalyst. Graphs a, b, c, d, and e are the graphical representation of different orders in concentration catalyst ([Cat]) using the concentration of the product, obtained from the analysis.

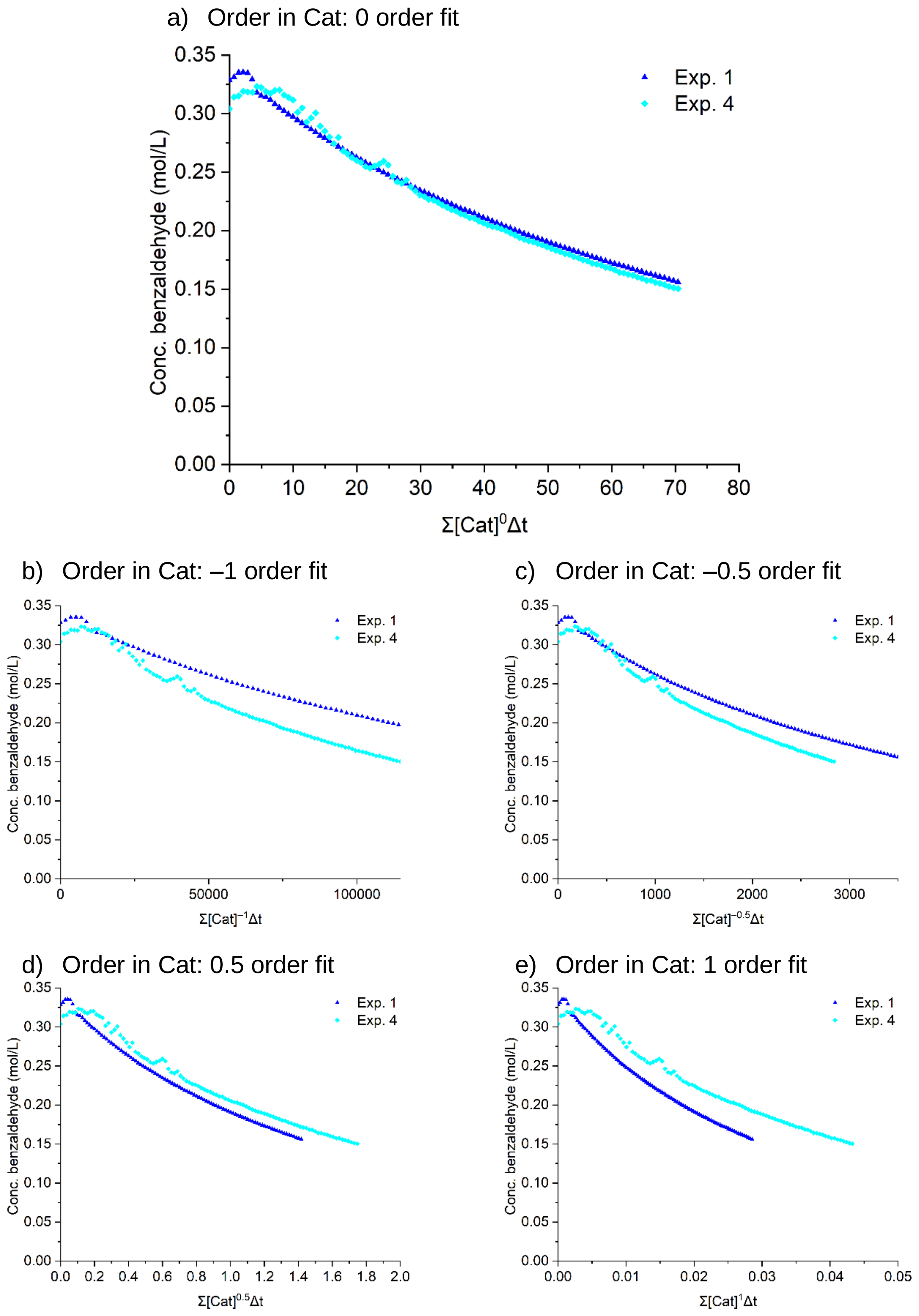

Figure S88. VTNA hydroboration benzaldehyde using HBBN dimer as reducing agent and 0.1 mol% [Na(18c6)]$_2$[**1**] as catalyst. Graphs a, b, c, d, and e are the graphical representation of different orders in concentration catalyst ([Cat]) using the concentration of benzaldehyde ([A]), obtained from the analysis.

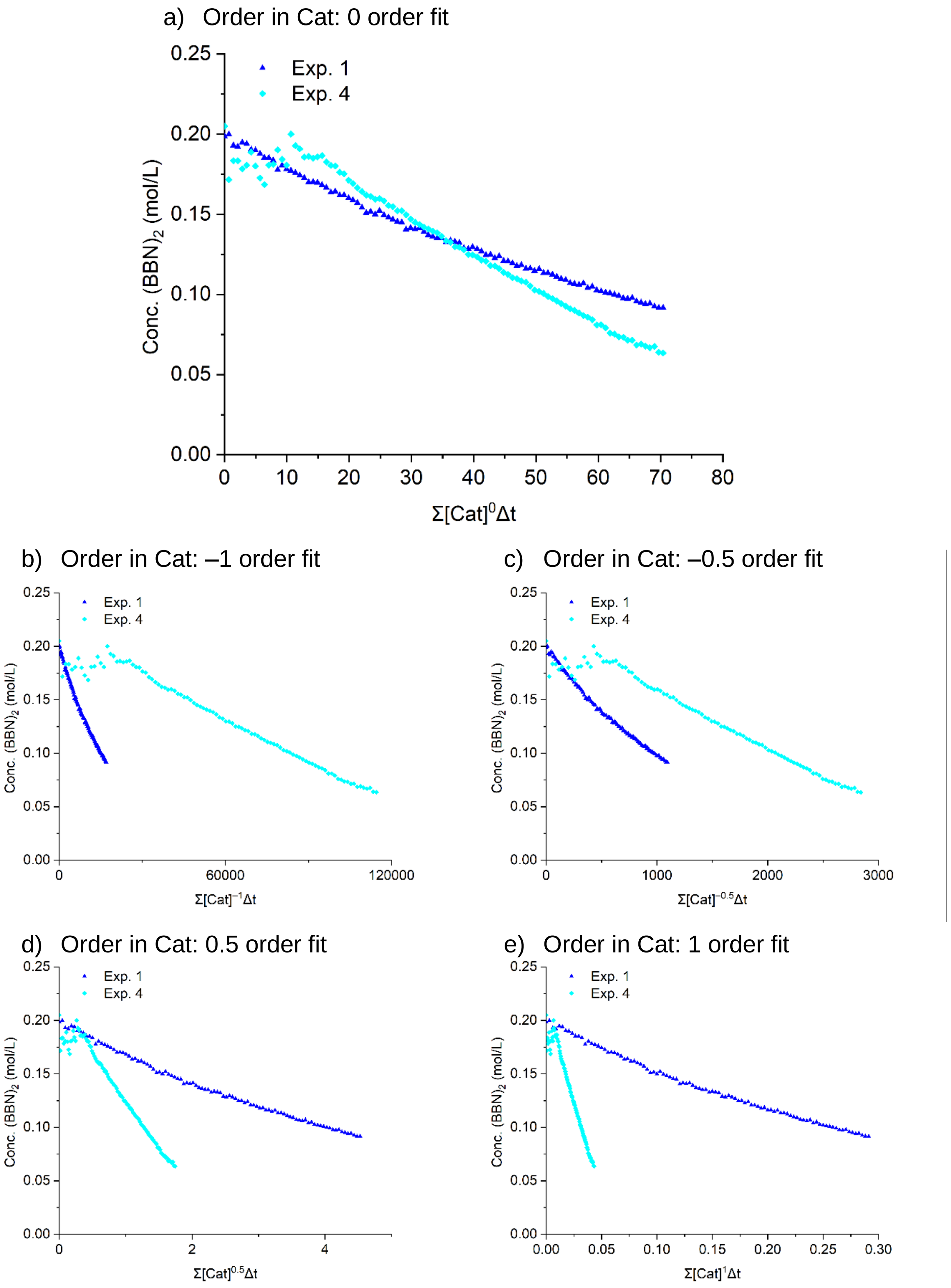

Figure S89. VTNA hydroboration benzaldehyde using HBBN dimer as reducing agent and 0.1 mol% $[Na(18c6)]_2$[**1**] as catalyst. Graphs a, b, c, d, and e are the graphical representation of different orders in concentration catalyst ([Cat]) using the concentration of HBBN dimer ([B]), obtained from the analysis.

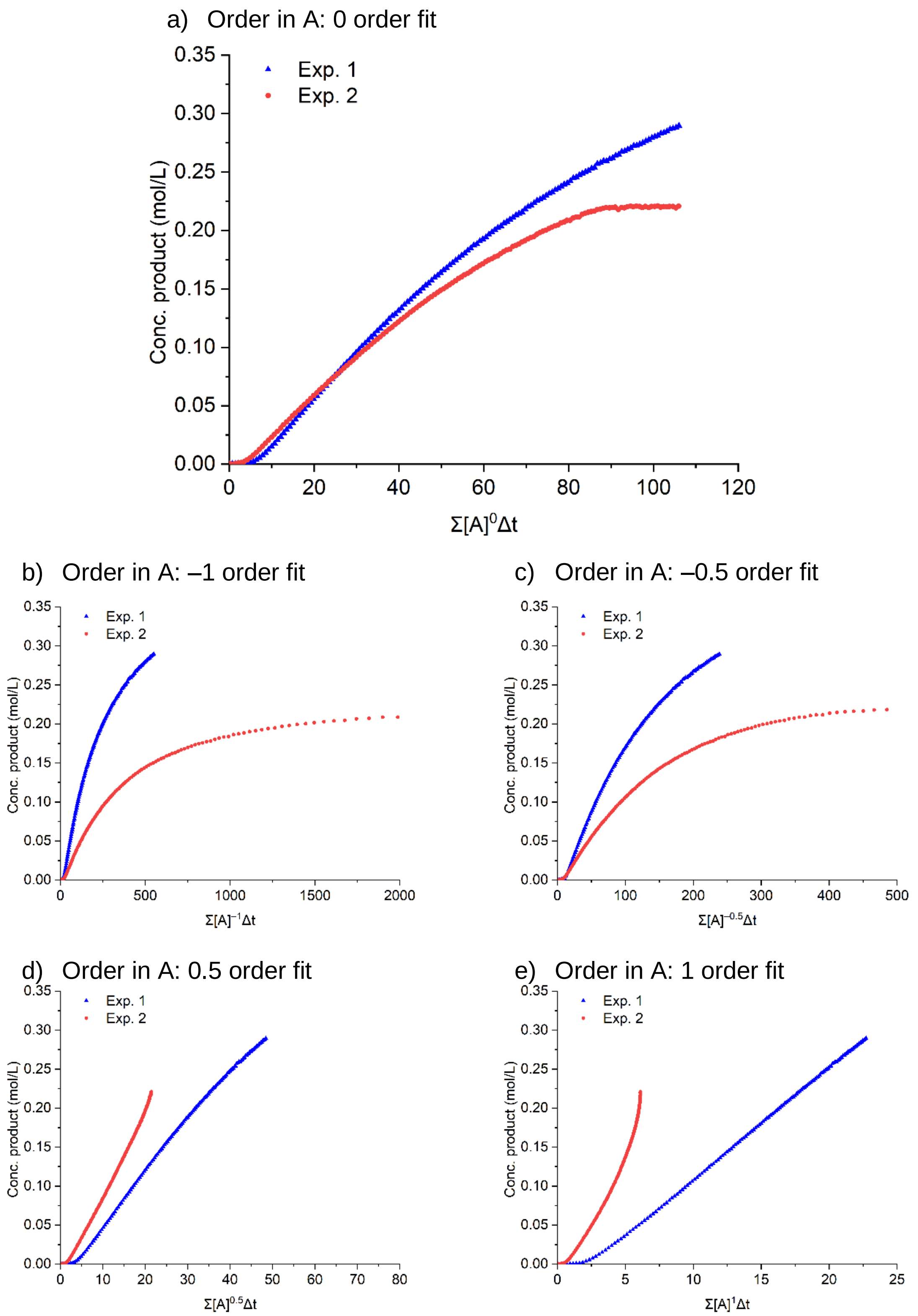

Figure S90. VTNA hydroboration benzaldehyde using HBBN dimer as reducing agent and 0.1 mol% $[Na(18c6)]_2$[**2**] as catalyst. Graphs a, b, c, d, and e are the graphical representation of different orders in concentration benzaldehyde ([A]) using the concentration of the product, obtained from the analysis.

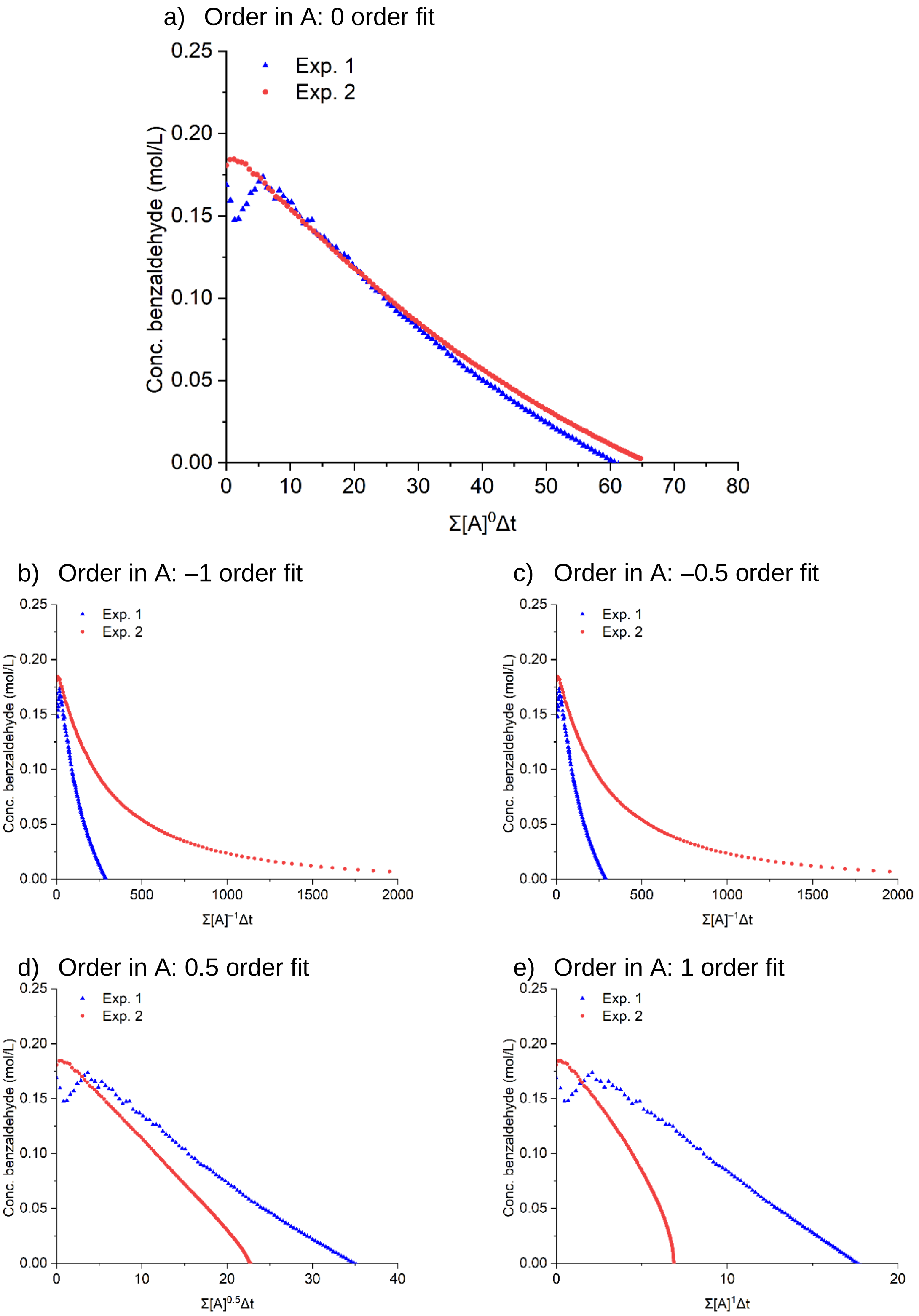

Figure S91. VTNA hydroboration benzaldehyde using HBBN dimer as reducing agent and 0.1 mol% $[Na(18c6)]_2$[**2**] as catalyst. Graphs a, b, c, d, and e are the graphical representation of different orders in concentration benzaldehyde ([A]) using the concentration of benzaldehyde ([A]), obtained from the analysis.

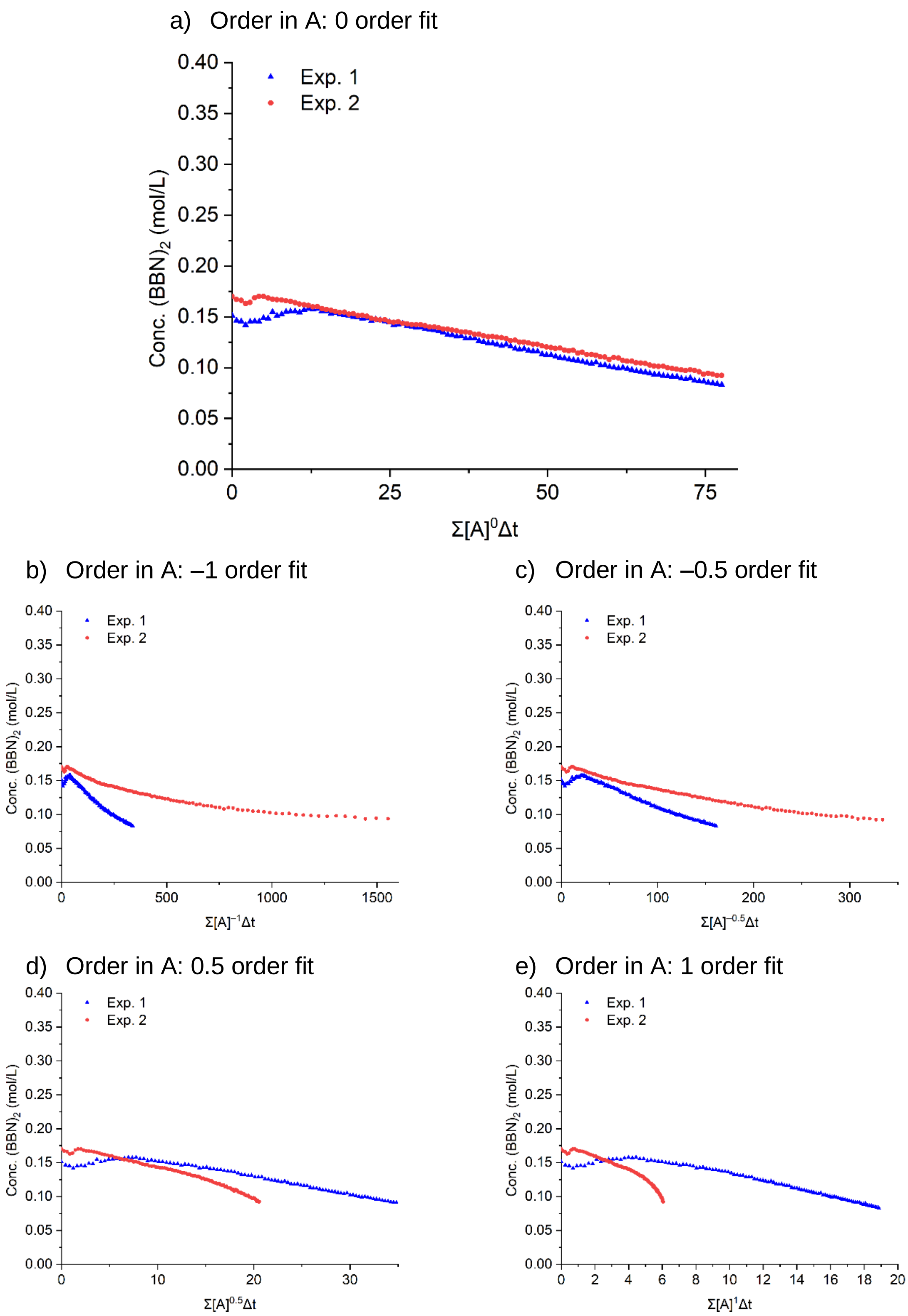

Figure S92. VTNA hydroboration benzaldehyde using HBBN dimer as reducing agent and 0.1 mol% $[Na(18c6)]_2$[**2**] as catalyst. Graphs a, b, c, d, and e are the graphical representation of different orders in concentration benzaldehyde ([A]) using the concentration of HBBN dimer ([B]), obtained from the analysis.

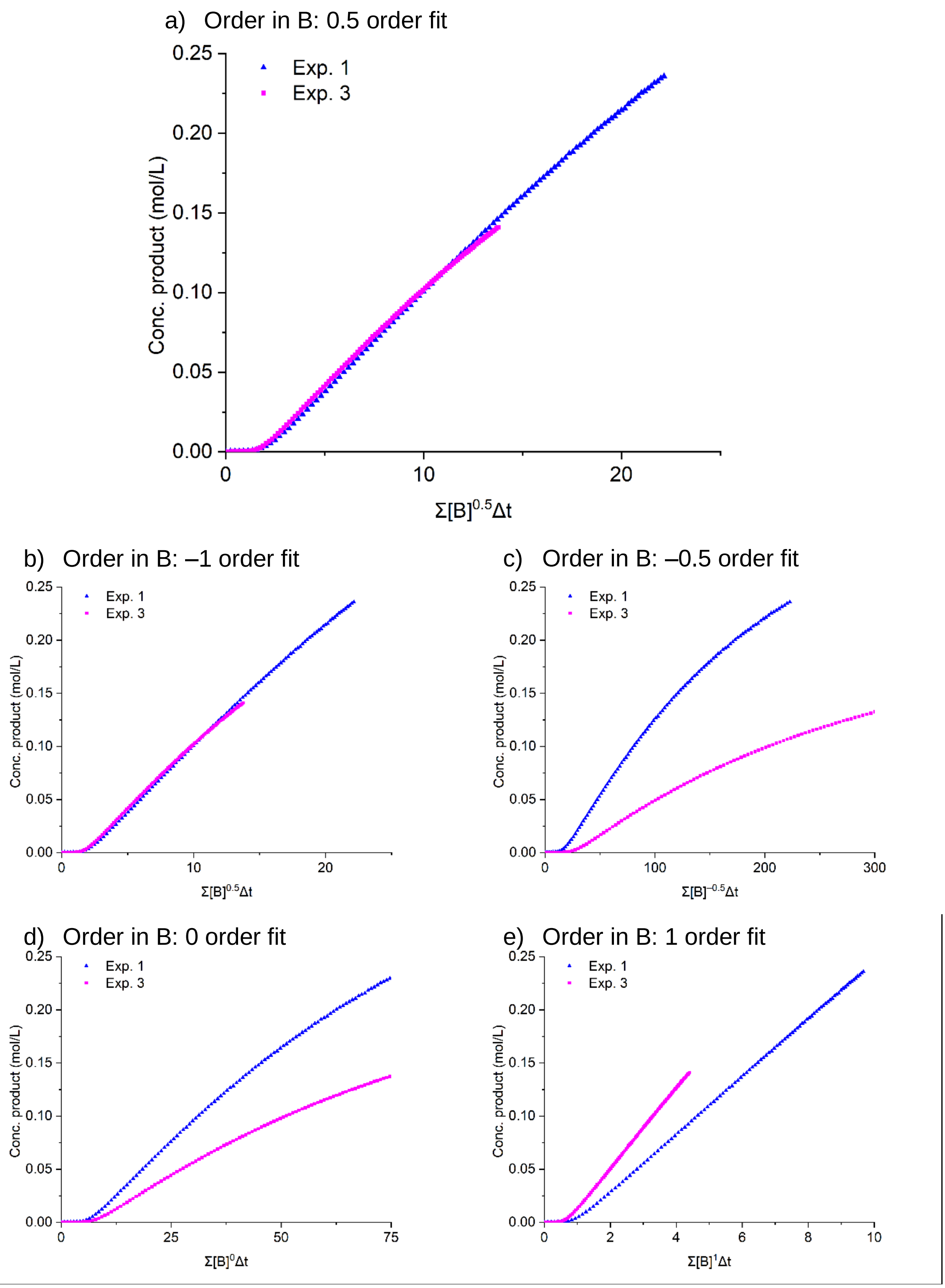

Figure S93. VTNA hydroboration benzaldehyde using HBBN dimer as reducing agent and 0.1 mol% $[Na(18c6)]_2$[**2**] as catalyst. Graphs a, b, c, d, and e are the graphical representation of different orders in concentration HBBN dimer ([B]) using the concentration of the product, obtained from the analysis.

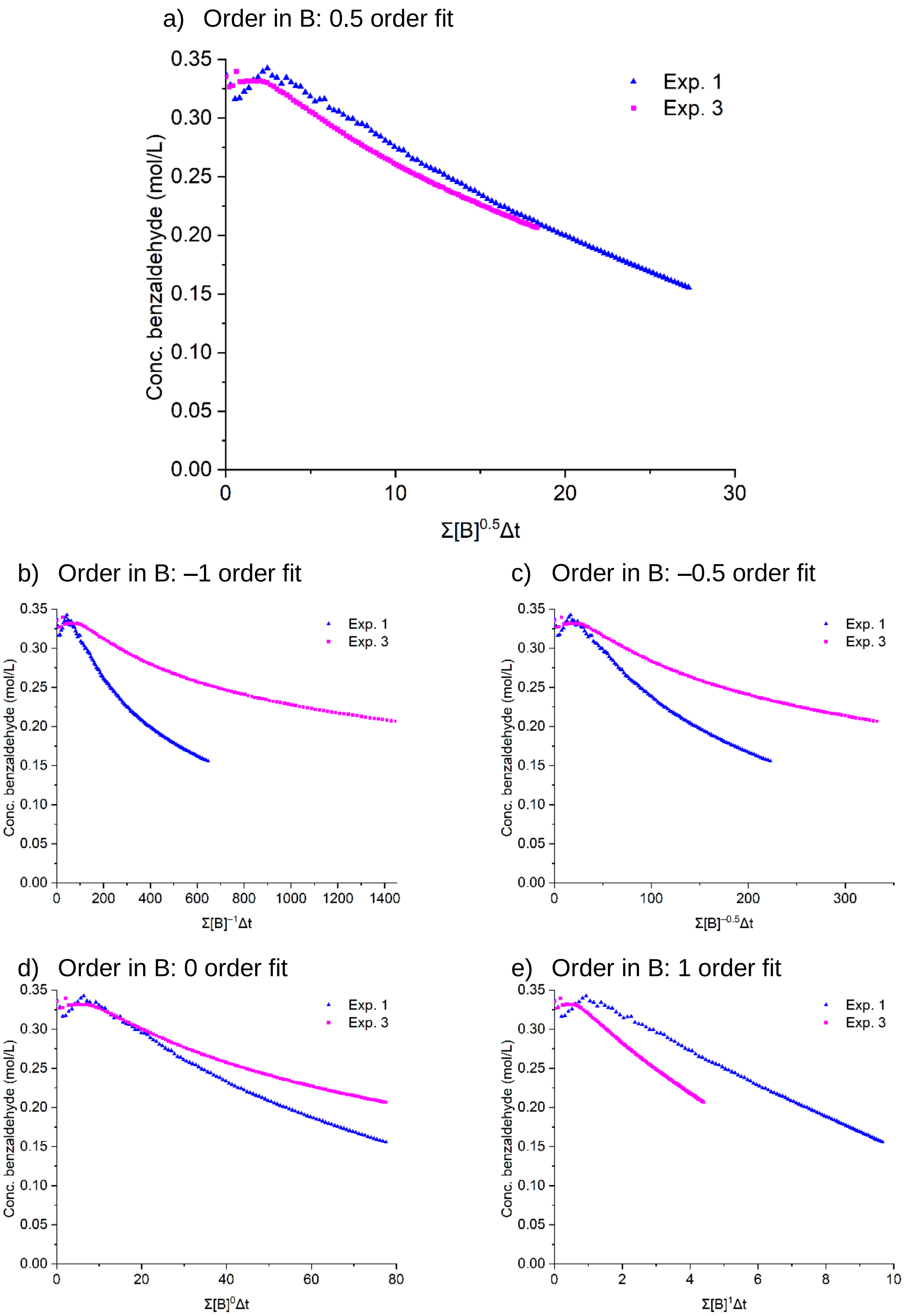

Figure S94. VTNA hydroboration benzaldehyde using HBBN dimer as reducing agent and 0.1 mol% [Na(18c6)]$_2$[**2**] as catalyst. Graphs a, b, c, d, and e are the graphical representation of different orders in concentration HBBN dimer ([B]) using the concentration of benzaldehyde ([A]), obtained from the analysis.

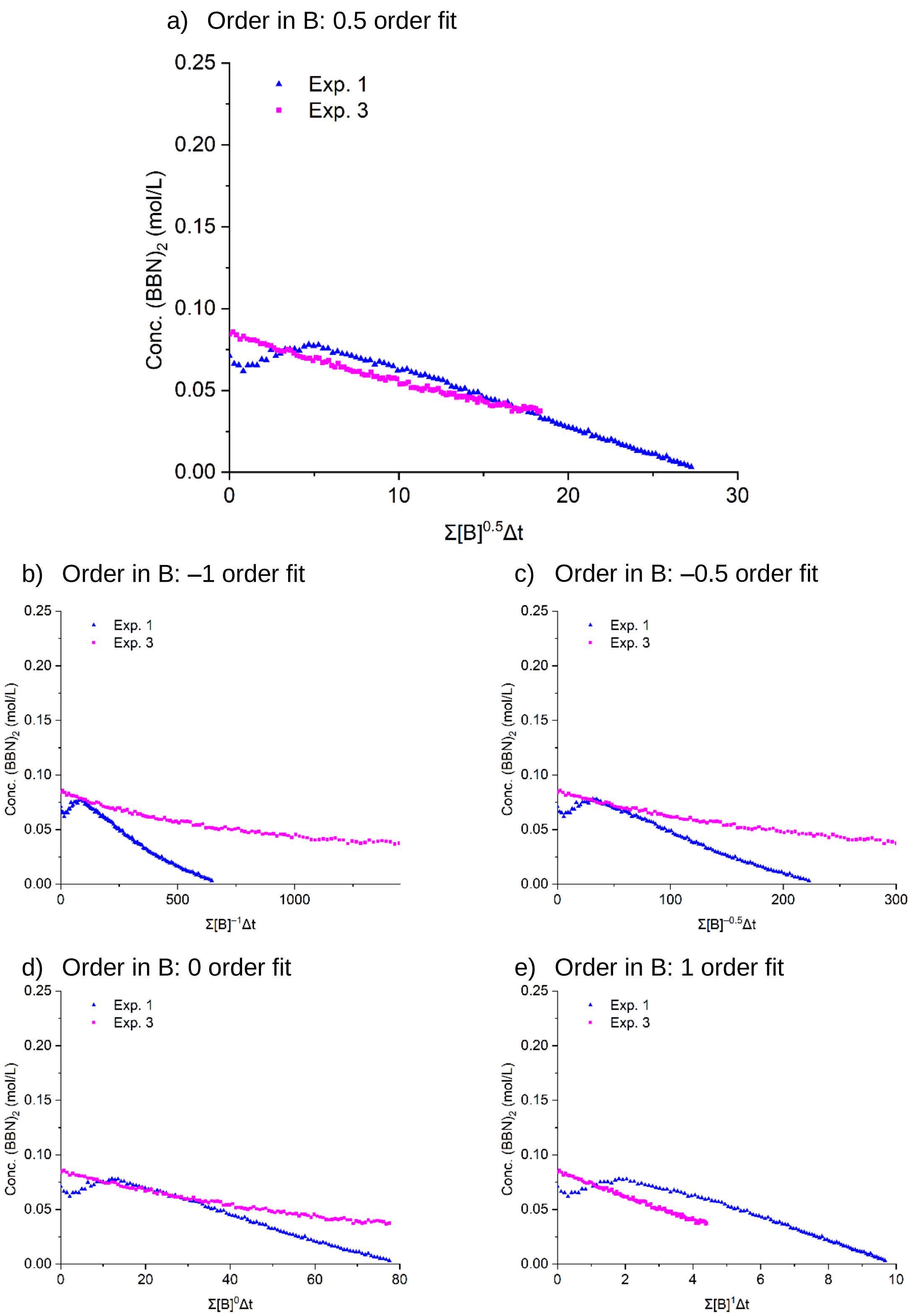

Figure S95. VTNA hydroboration benzaldehyde using HBBN dimer as reducing agent and 0.1 mol% $[Na(18c6)]_2$[**2**] as catalyst. Graphs a, b, c, d, and e are the graphical representation of different orders in concentration HBBN dimer ([B]) using the concentration of HBBN dimer ([B]), obtained from the analysis.

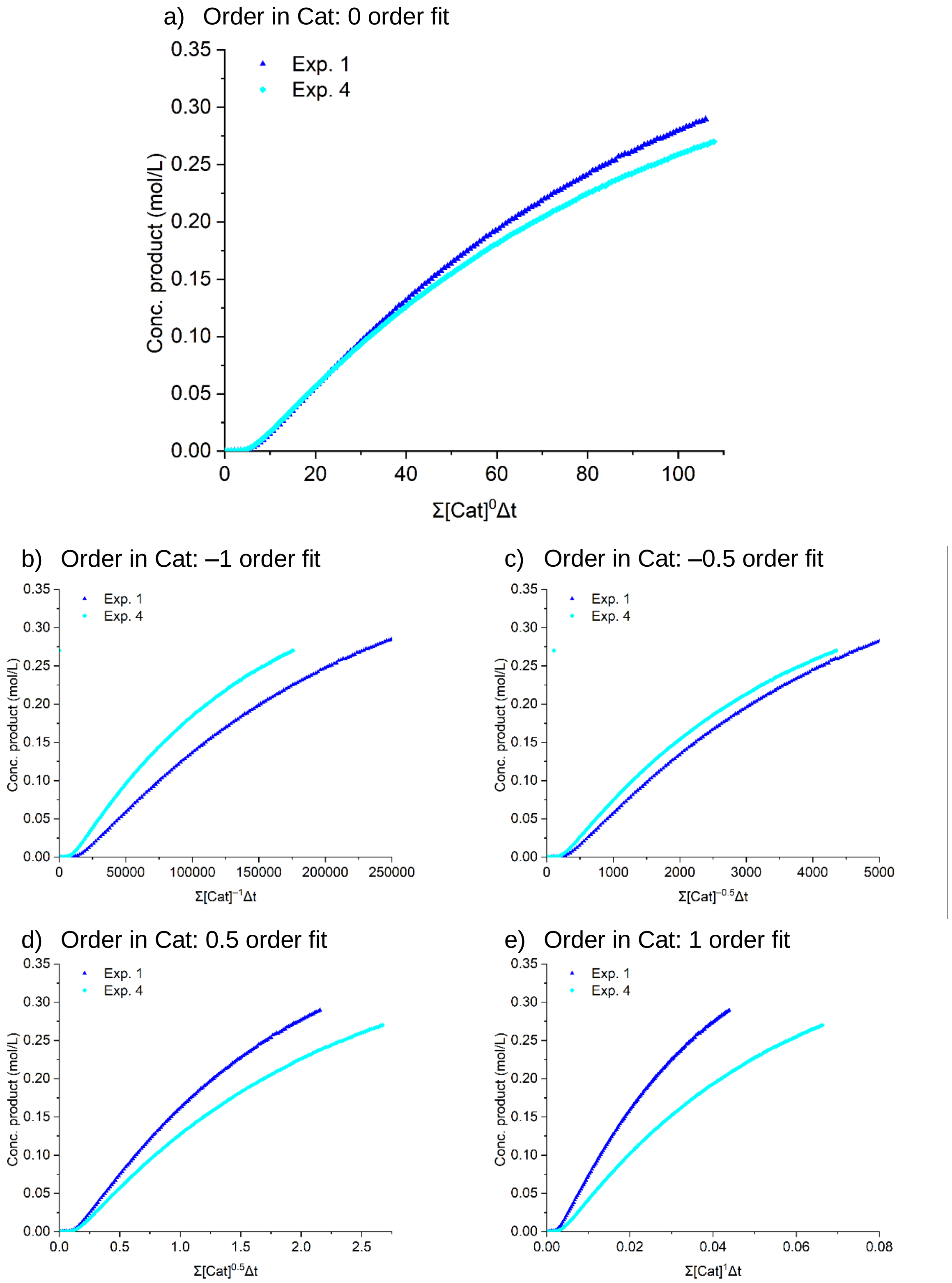

Figure S96. VTNA hydroboration benzaldehyde using HBBN dimer as reducing agent and 0.1 mol% [Na(18c6)]$_2$[**2**] as catalyst. Graphs a, b, c, d, and e are the graphical representation of different orders in concentration catalyst ([Cat]) using the concentration of the product, obtained from the analysis.

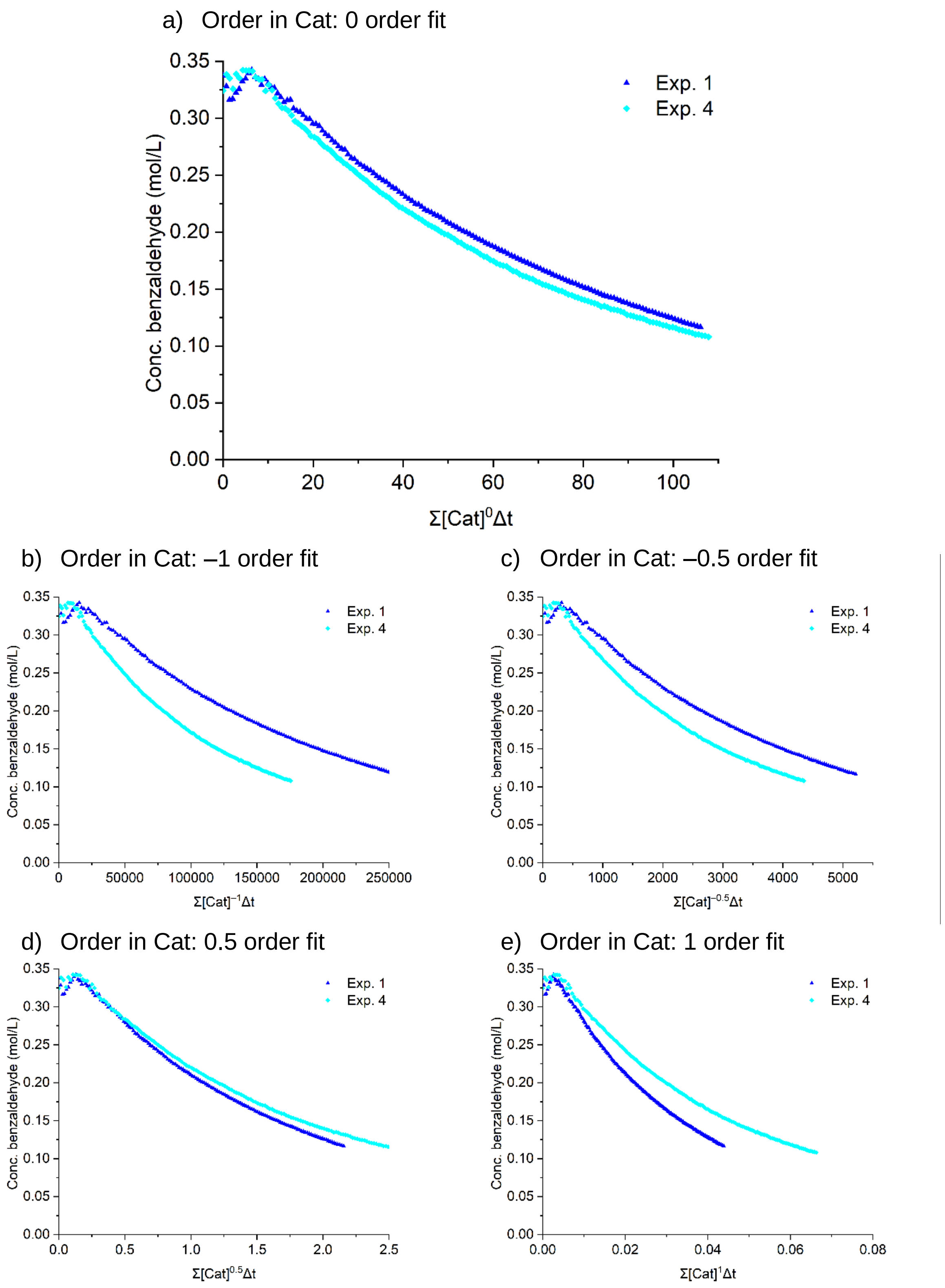

Figure S97. VTNA hydroboration benzaldehyde using HBBN dimer as reducing agent and 0.1 mol% [Na(18c6)]$_2$[**2**] as catalyst. Graphs a, b, c, d, and e are the graphical representation of different orders in concentration catalyst ([Cat]) using the concentration of benzaldehyde ([A]), obtained from the analysis.

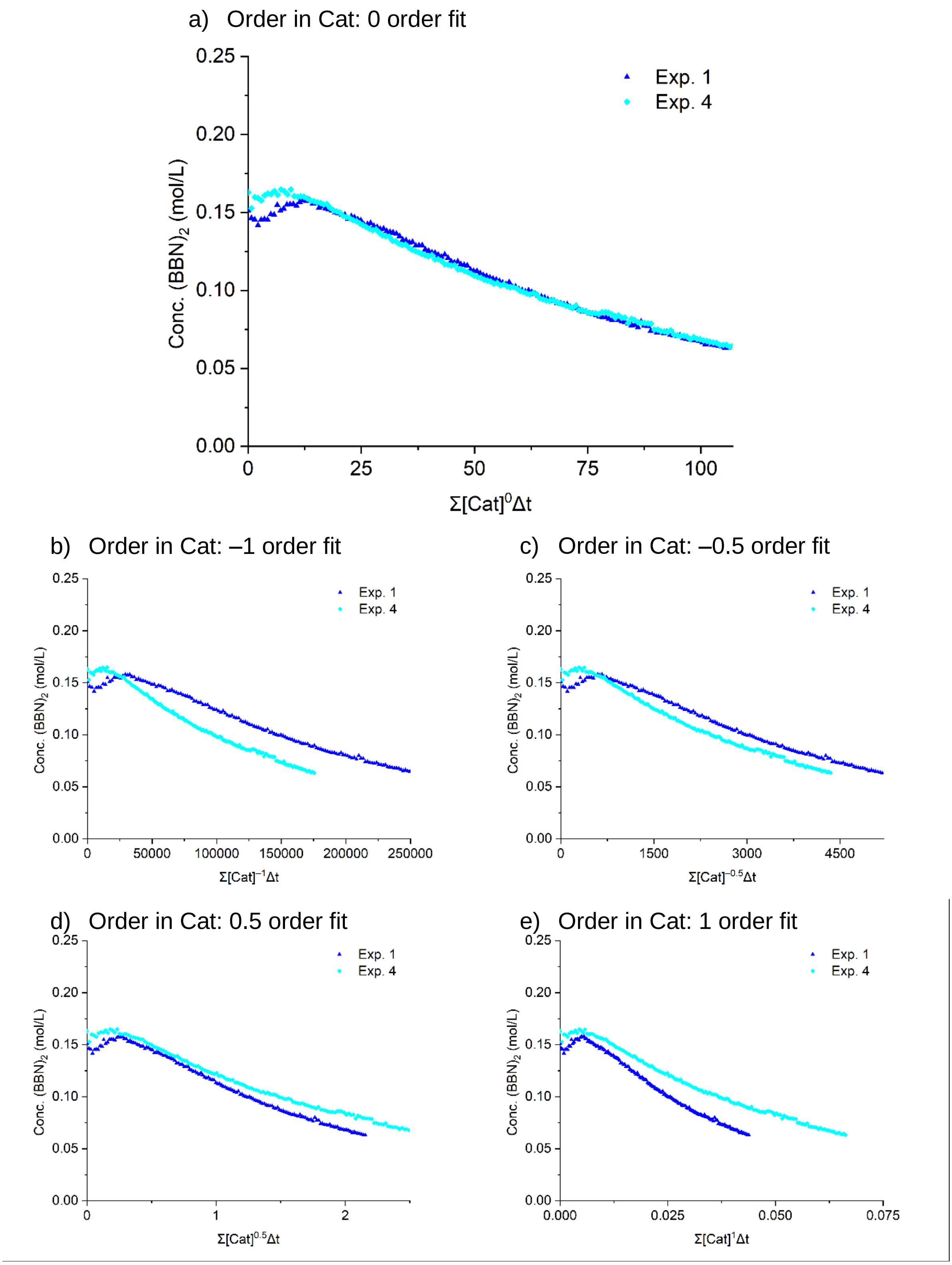

Figure S98. VTNA hydroboration benzaldehyde using HBBN dimer as reducing agent and 0.1 mol% $[Na(18c6)]_2$[**2**] as catalyst. Graphs a, b, c, d, and e are the graphical representation of different orders in concentration catalyst ([Cat]) using the concentration of HBBN dimer ([B]), obtained from the analysis.

5.2. Order in Reagents of the Hydroboration of Benzaldehyde Using HBpin

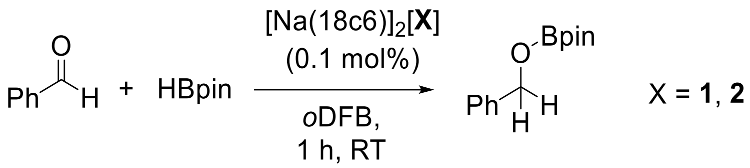

Concentrations of the product (^{1}H δ = 5.02 ppm), HBpin (^{1}H δ = 1.28 ppm), and benzaldehyde (^{1}H δ = 9.98 ppm) were calculated by integration of the ^{1}H NMR spectrum using toluene as an internal standard (^{1}H δ = 2.31 ppm). Following the variable time normalization analysis as described by Burés,[28, 29] the reaction order can be obtained via a graphical representation. The analysis supports a fitting of a zero order in the concentration of aldehyde and first order in the concentration of HBpin and, in the concentration of catalysts, represented in Formula 2. The same observation was made using 0.5 mol% catalysts loading.

Formula 2. $[\mathrm{P}] = \mathrm{k}\int[\mathrm{Benzaldehyde}]^0 \times [\mathrm{HBpin}]^1 \times [\mathrm{Catalyst}]^1\,\mathrm{dt}$

Table S5. VTNA experiment hydroboration of benzaldehyde using HBpin.

Exp.	Added Benzaldehyde (mmol) (A)	Added HBpin (mmol) (B)	Added Catalyst (mmol) (Cat)
1 (▲)	0.22	0.22	0.0002
2 (●)	0.11	0.22	0.0002
3 (●)	0.33	0.22	0.0002
4 (■)	0.22	0.11	0.0002
5 (■)	0.22	0.33	0.0002
6 (◆)	0.22	0.22	0.0001
7 (◆)	0.22	0.22	0.0003

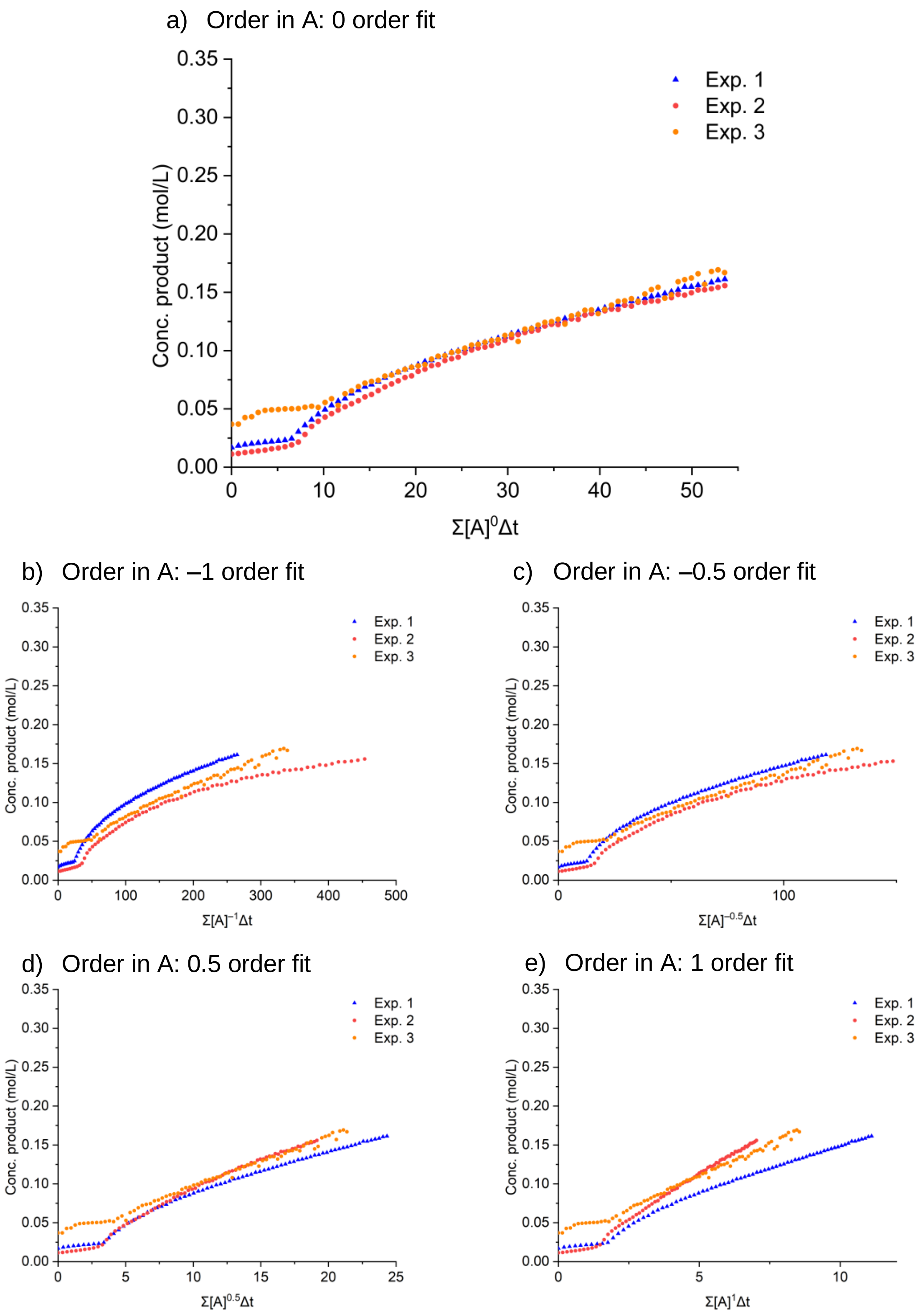

Figure S99. VTNA hydroboration benzaldehyde using HBpin as reducing agent and 0.1 mol% $[Na(18c6)]_2$[**1**] as catalyst. Graphs a, b, c, d, and e are the graphical representation of different orders in concentration benzaldehyde ([A]) using the concentration of the product, obtained from the analysis.

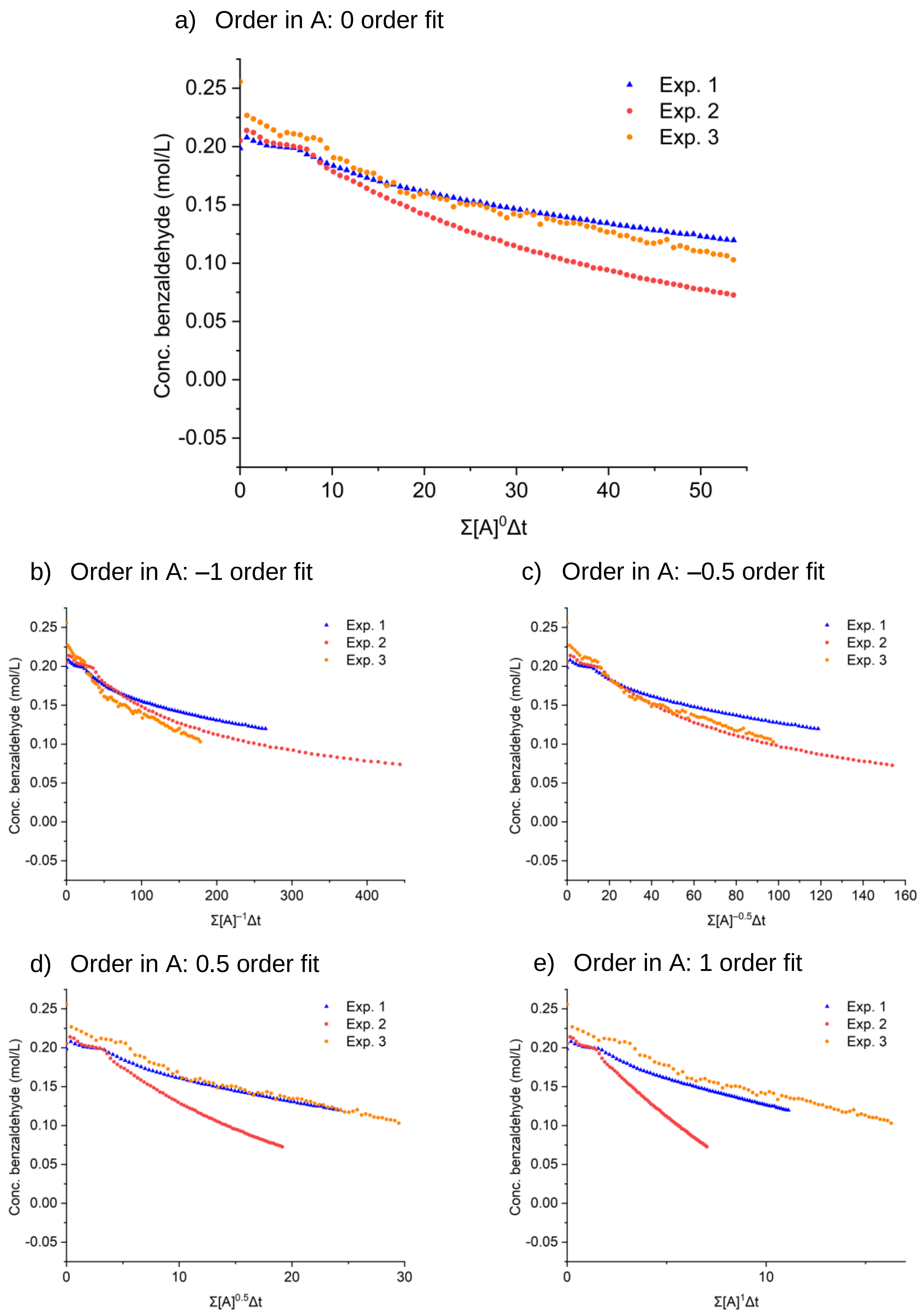

Figure S100. VTNA hydroboration benzaldehyde using HBpin as reducing agent and 0.1 mol% $[Na(18c6)]_2$[**1**] as catalyst. Graphs a, b, c, d, and e are the graphical representation of different orders in concentration benzaldehyde ([A]) using the concentration of benzaldehyde ([A]), obtained from the analysis.

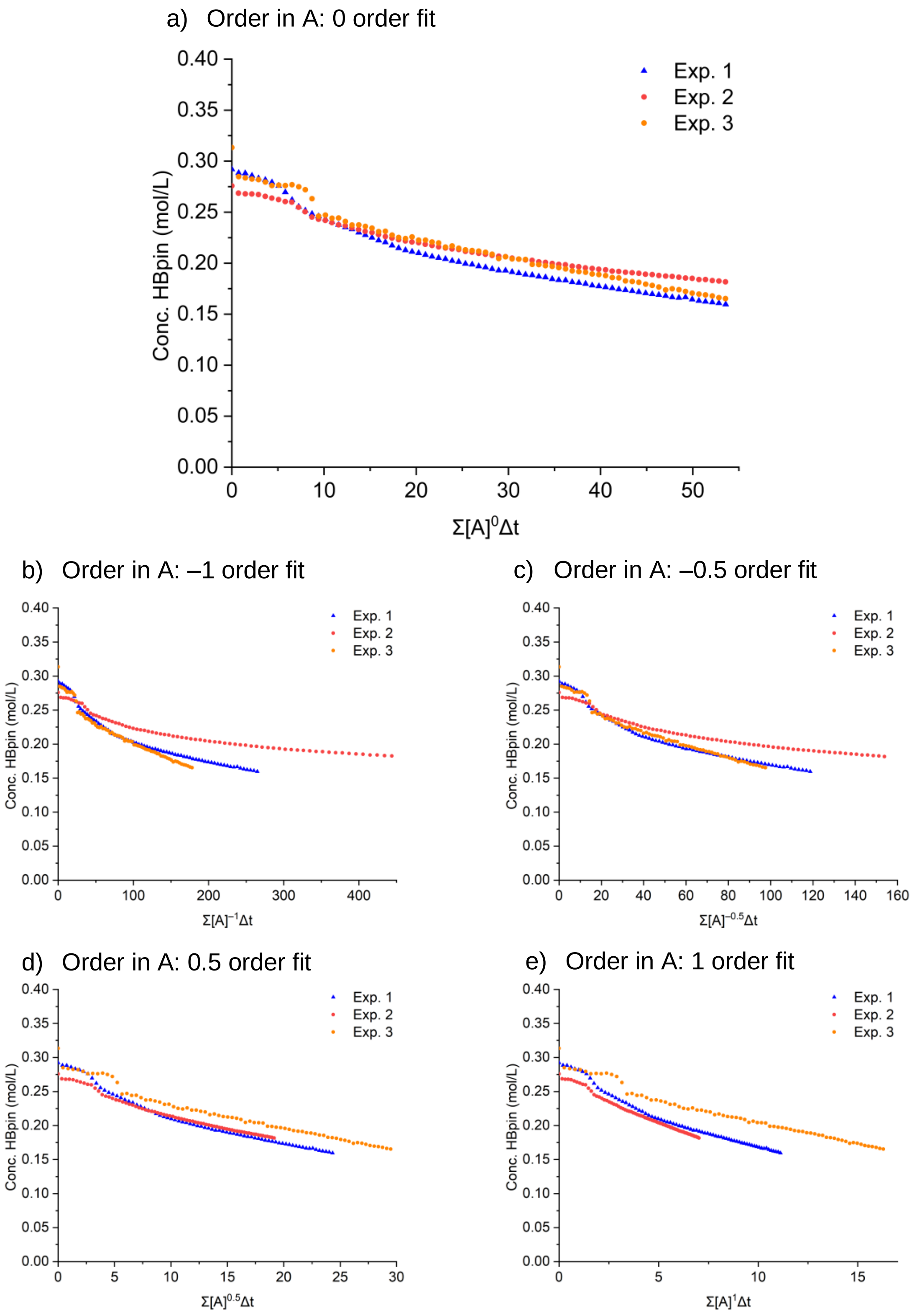

Figure S101. VTNA hydroboration benzaldehyde using HBpin as reducing agent and 0.1 mol% [Na(18c6)]$_2$[**1**] as catalyst. Graphs a, b, c, d, and e are the graphical representation of different orders in concentration benzaldehyde ([A]) using the concentration of HBpin ([B]), obtained from the analysis.

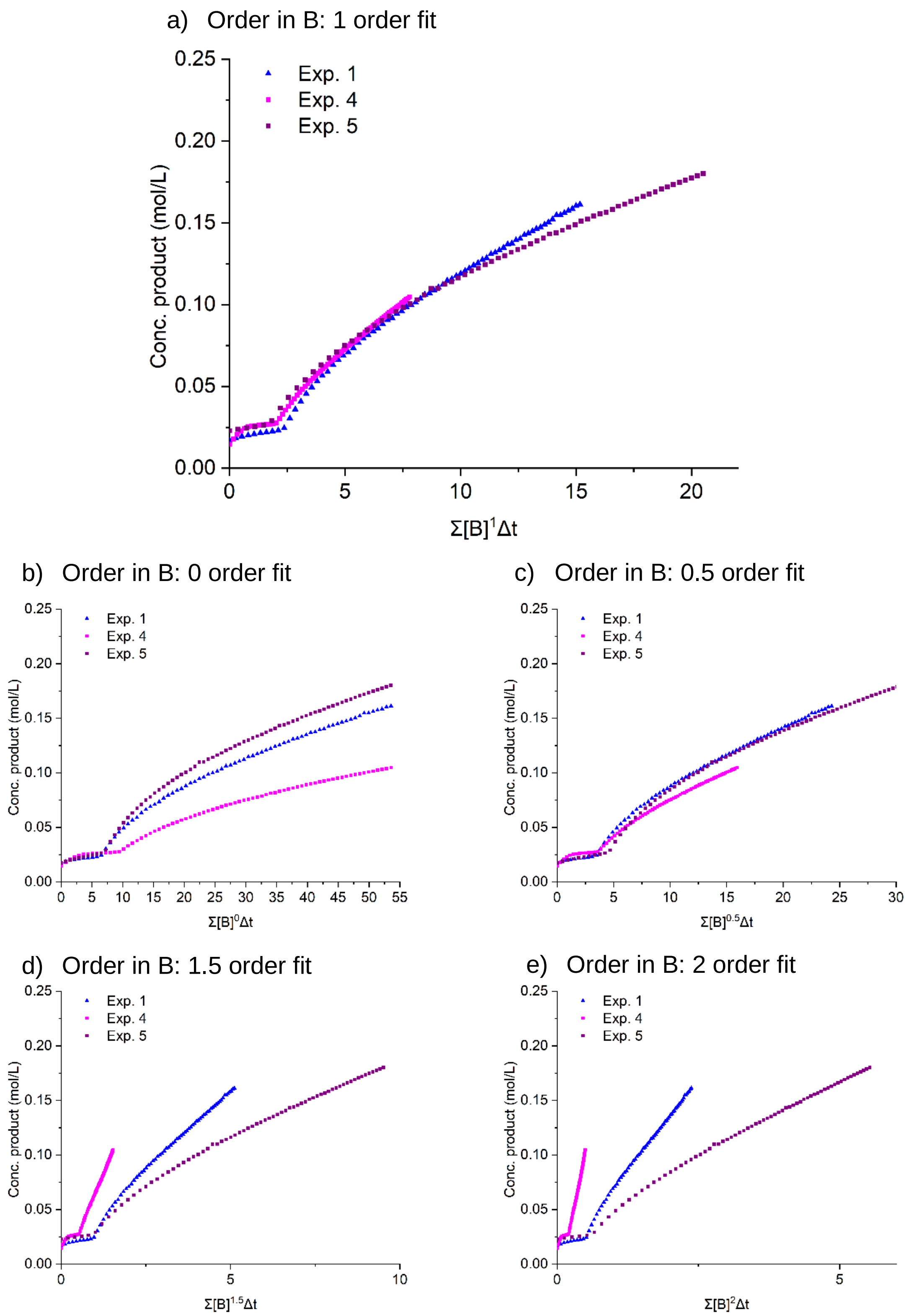

Figure S102. VTNA hydroboration benzaldehyde using HBpin as reducing agent and 0.1 mol% $[Na(18c6)]_2$[**1**] as catalyst. Graphs a, b, c, d, and e are the graphical representation of different orders in concentration HBpin ([B]) using the concentration of the product, obtained from the analysis.

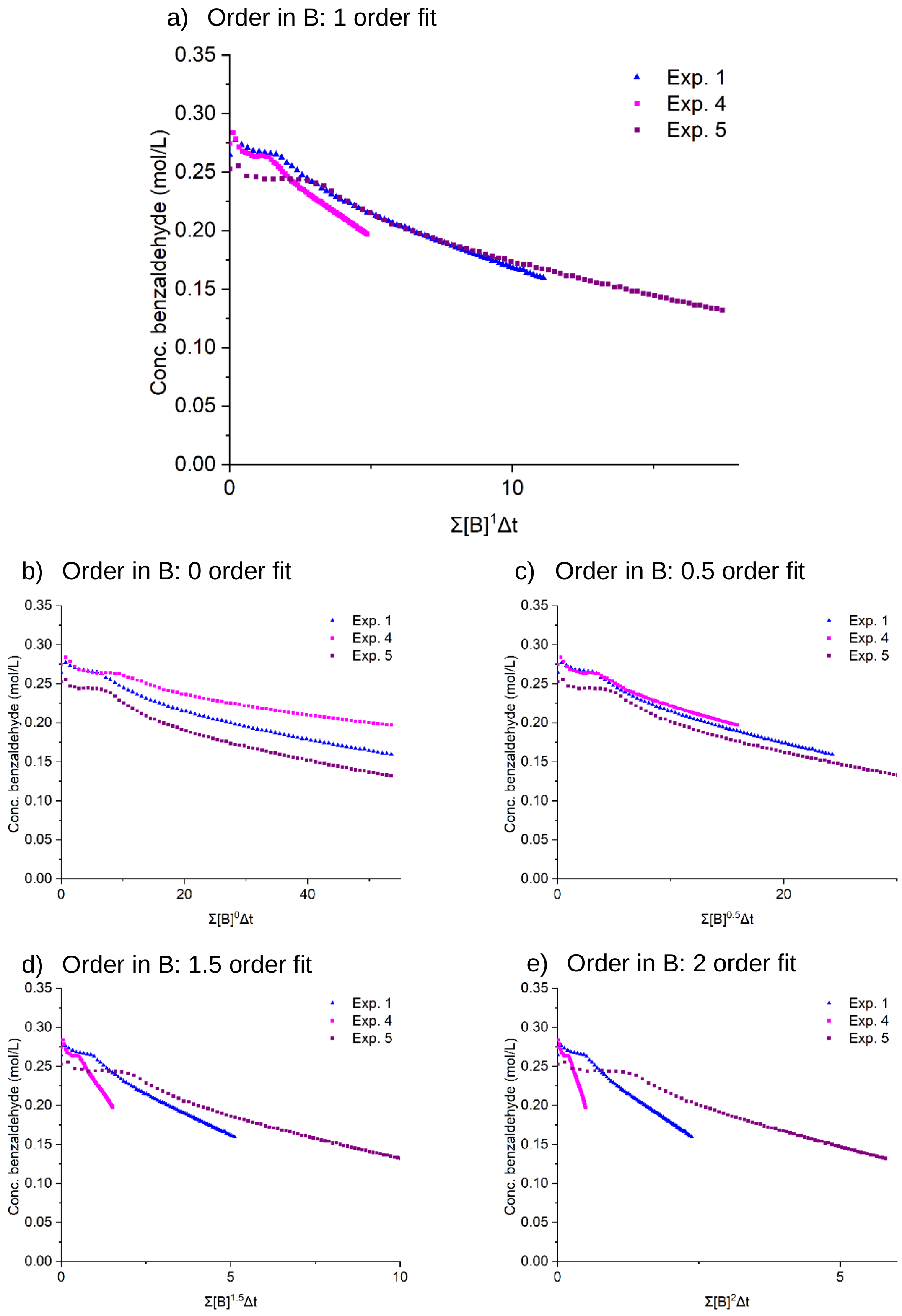

Figure S103. VTNA hydroboration benzaldehyde using HBpin as reducing agent and 0.1 mol% [Na(18c6)]$_2$[**1**] as catalyst. Graphs a, b, c, d, and e are the graphical representation of different orders in concentration HBpin ([B]) using the concentration of benzaldehyde ([A]), obtained from the analysis.

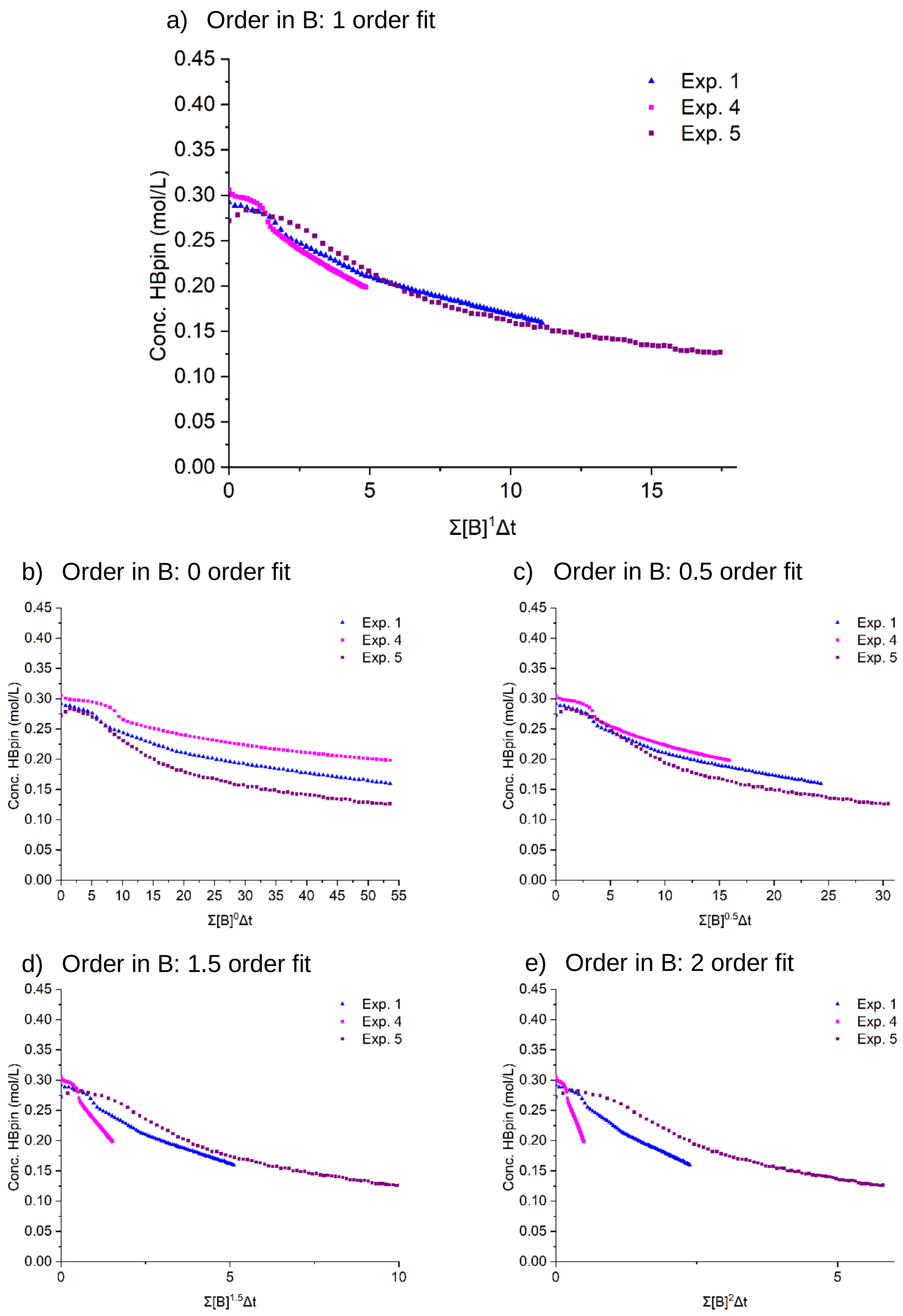

Figure S104. VTNA hydroboration benzaldehyde using HBpin as reducing agent and 0.1 mol% [Na(18c6)]$_2$[**1**] as catalyst. Graphs a, b, c, d, and e are the graphical representation of different orders in concentration HBpin ([B]) using the concentration of HBpin ([B]), obtained from the analysis.

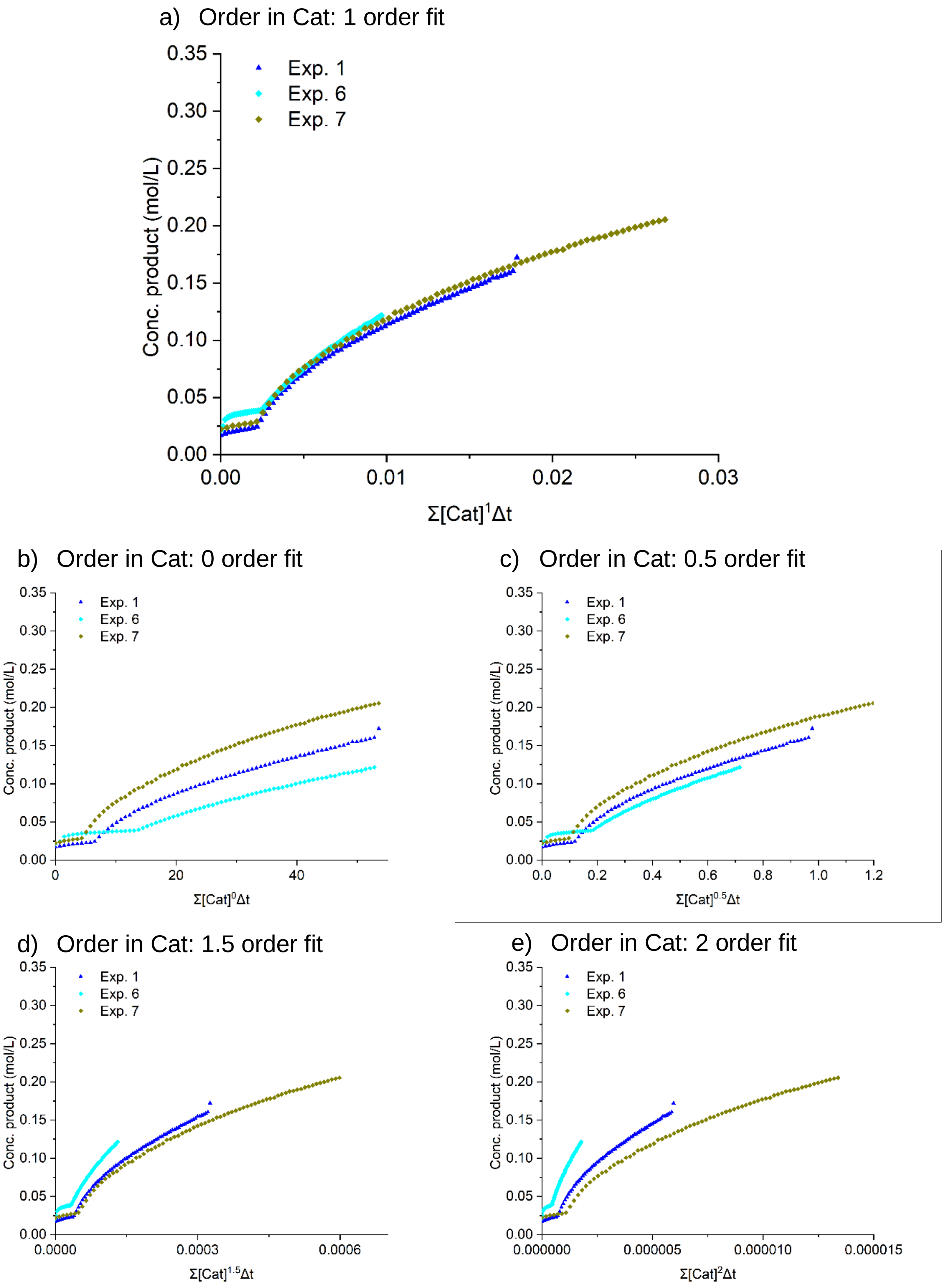

Figure S105. VTNA hydroboration benzaldehyde using HBpin as reducing agent and 0.1 mol% $[Na(18c6)]_2$[**1**] as catalyst. Graphs a, b, c, d, and e are the graphical representation of different orders in concentration catalyst ([Cat]) using the concentration of the product, obtained from the analysis.

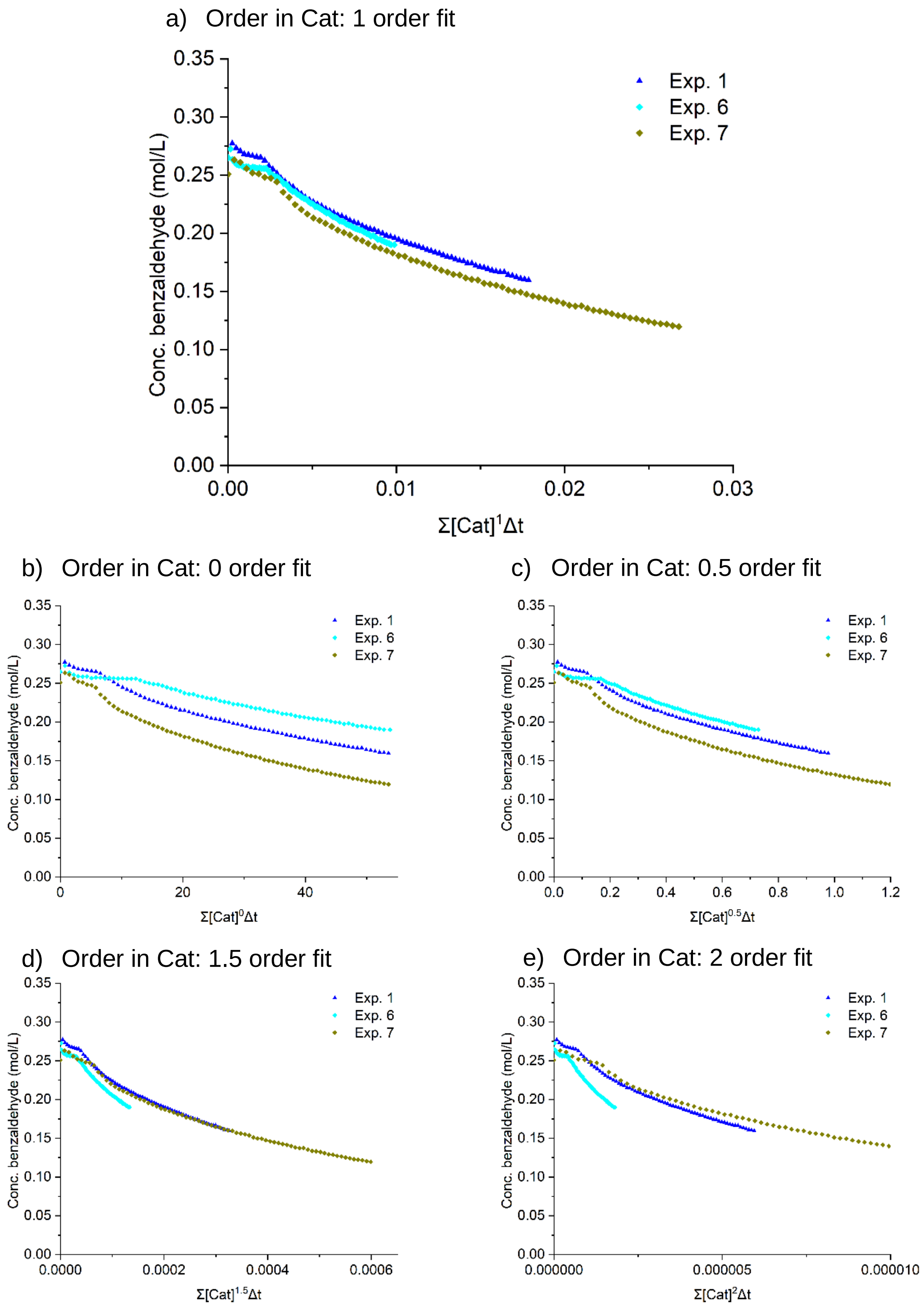

Figure S106. VTNA hydroboration benzaldehyde using HBpin as reducing agent and 0.1 mol% $[Na(18c6)]_2$[**1**] as catalyst. Graphs a, b, c, d, and e are the graphical representation of different orders in concentration catalyst ([Cat]) using the concentration of benzaldehyde ([A]), obtained from the analysis.

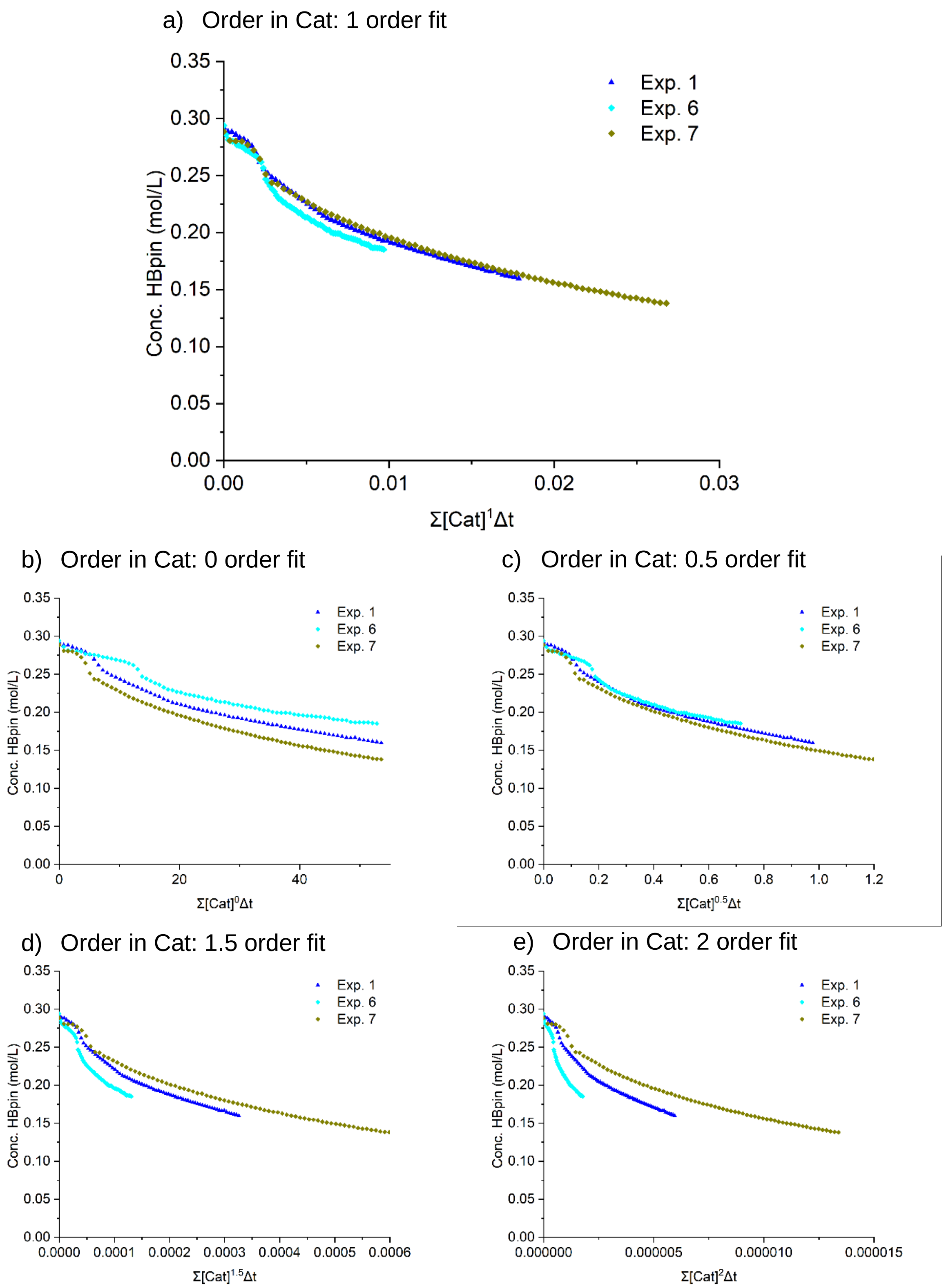

Figure S107. VTNA hydroboration benzaldehyde using HBpin as reducing agent and 0.1 mol% [Na(18c6)]$_2$[**1**] as catalyst. Graphs a, b, c, d, and e are the graphical representation of different orders in concentration catalyst ([Cat]) using the concentration of HBpin ([B]), obtained from the analysis.

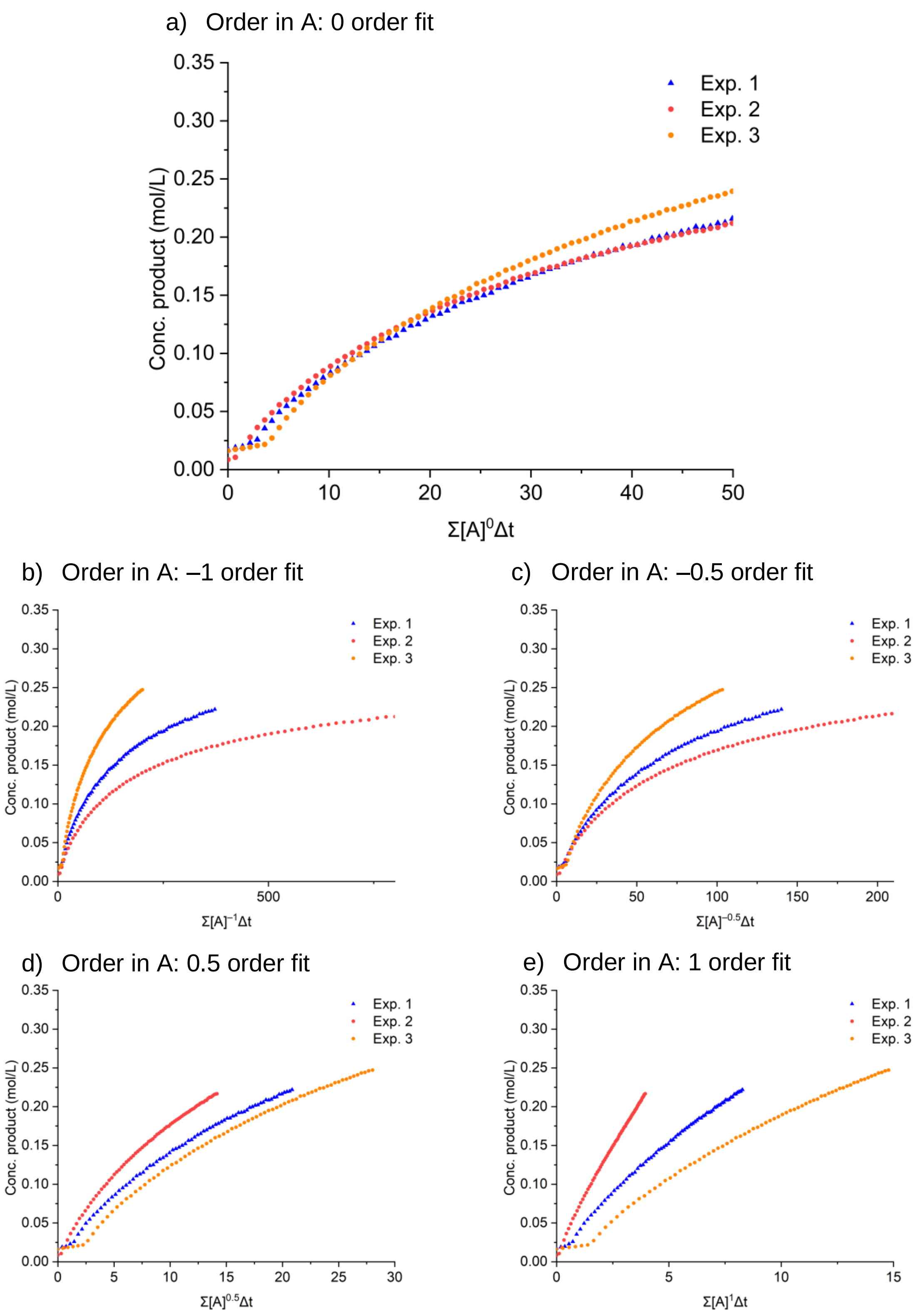

Figure S108. VTNA hydroboration benzaldehyde using HBpin as reducing agent and 0.1 mol% $[Na(18c6)]_2$[**2**] as catalyst. Graphs a, b, c, d, and e are the graphical representation of different orders in concentration benzaldehyde ([A]) using the concentration of the product, obtained from the analysis.

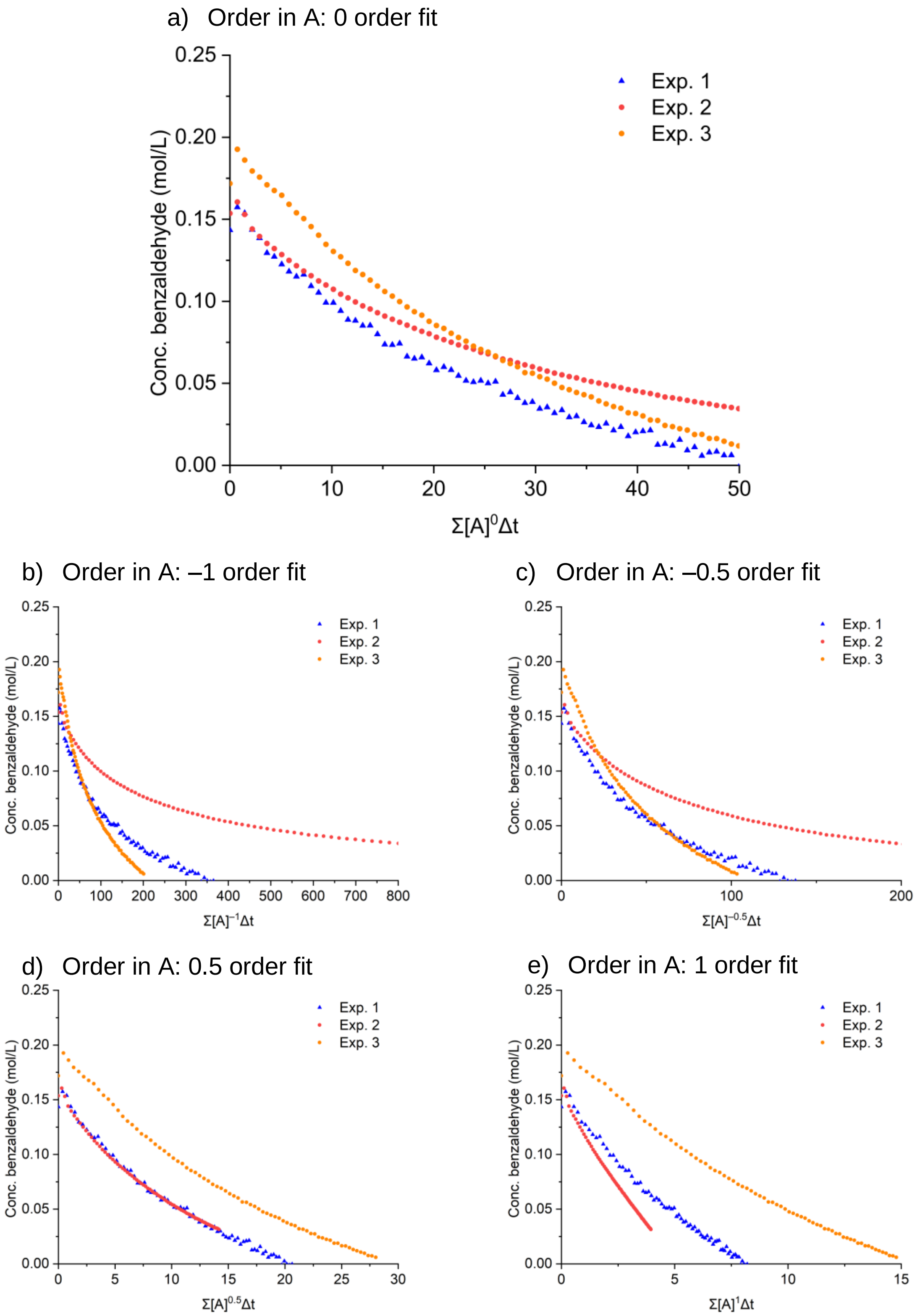

Figure S109. VTNA hydroboration benzaldehyde using HBpin as reducing agent and 0.1 mol% [Na(18c6)]$_2$[**2**] as catalyst. Graphs a, b, c, d, and e are the graphical representation of different orders in concentration benzaldehyde ([A]) using the concentration of benzaldehyde ([A]), obtained from the analysis.

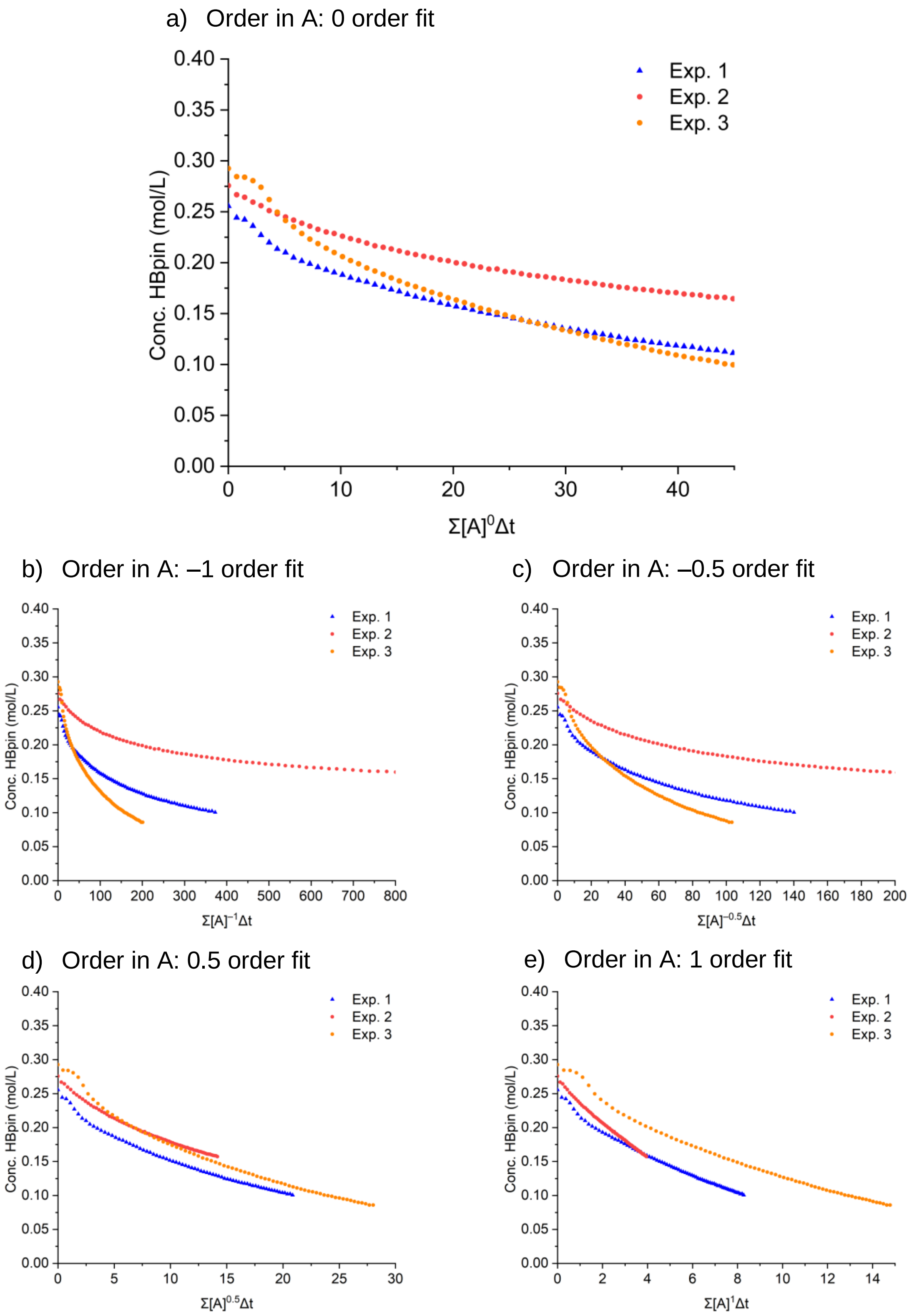

Figure S110. VTNA hydroboration benzaldehyde using HBpin as reducing agent and 0.1 mol% $[Na(18c6)]_2$[**2**] as catalyst. Graphs a, b, c, d, and e are the graphical representation of different orders in concentration benzaldehyde ([A]) using the concentration of HBpin ([B]), obtained from the analysis.

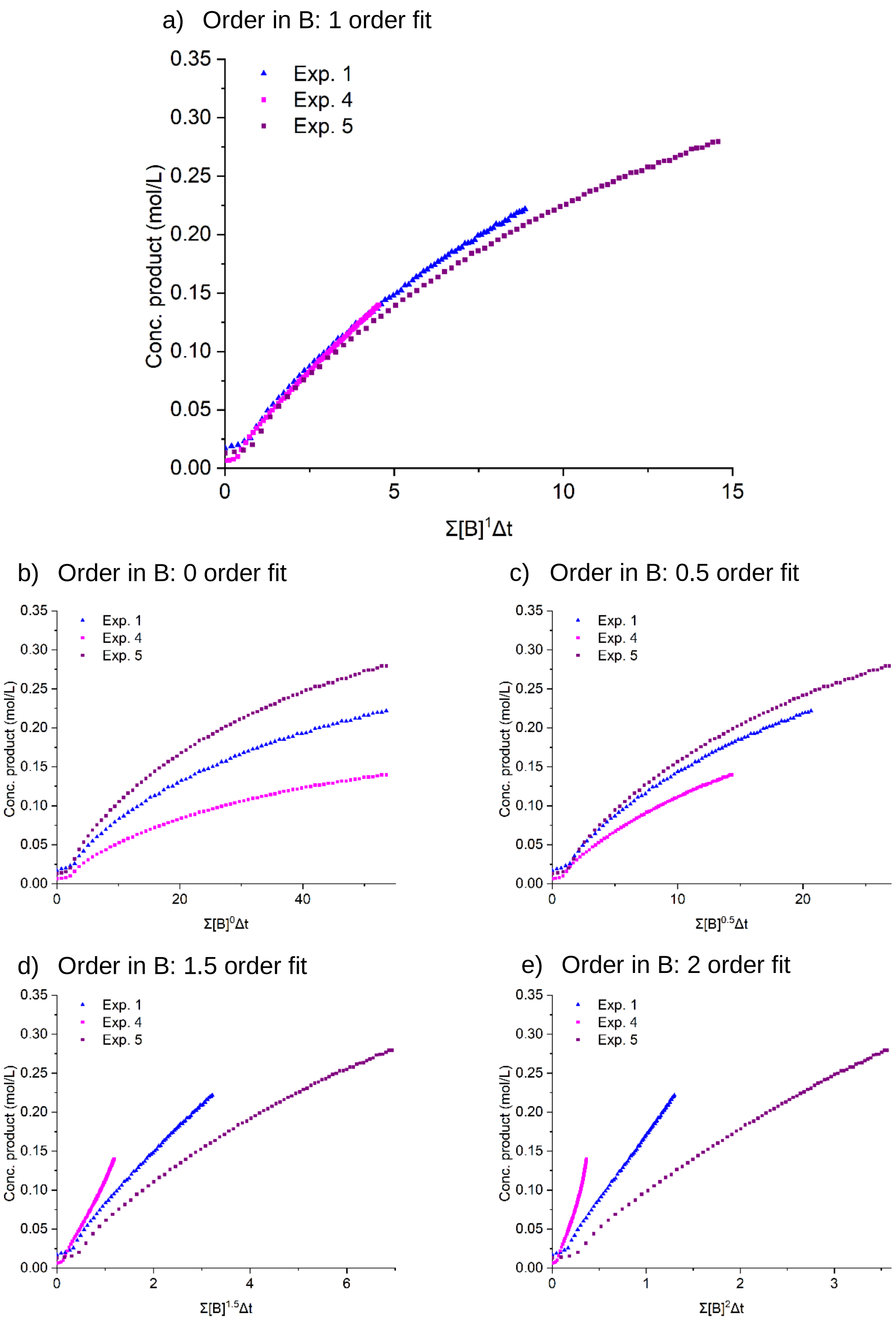

Figure S111. VTNA hydroboration benzaldehyde using HBpin as reducing agent and 0.1 mol% [Na(18c6)]$_2$[**2**] as catalyst. Graphs a, b, c, d, and e are the graphical representation of different orders in concentration HBpin ([B]) using the concentration of the product, obtained from the analysis.

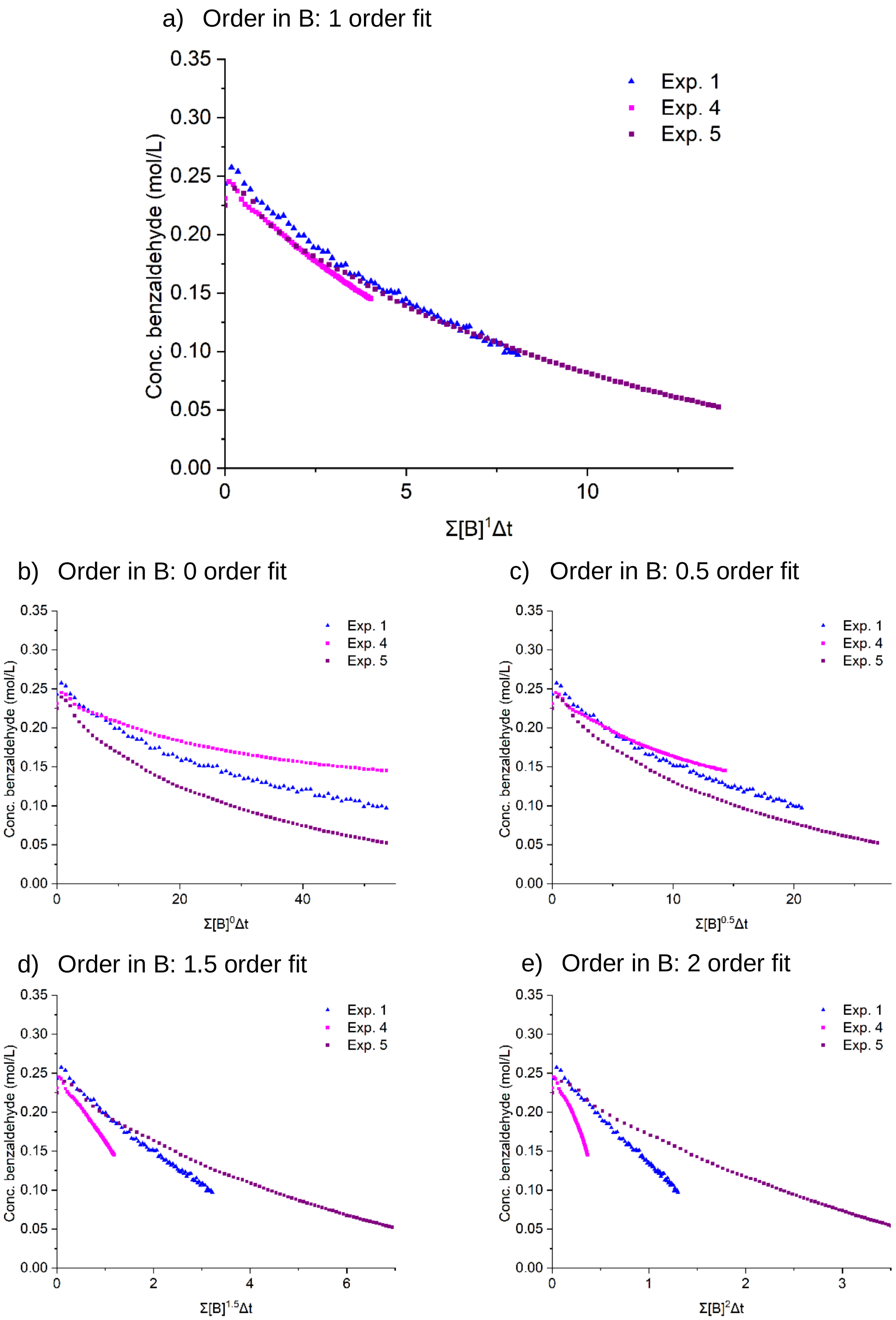

Figure S112. VTNA hydroboration benzaldehyde using HBpin as reducing agent and 0.1 mol% [Na(18c6)]$_2$[**2**] as catalyst. Graphs a, b, c, d, and e are the graphical representation of different orders in concentration HBpin ([B]) using the concentration of benzaldehyde ([A]), obtained from the analysis.

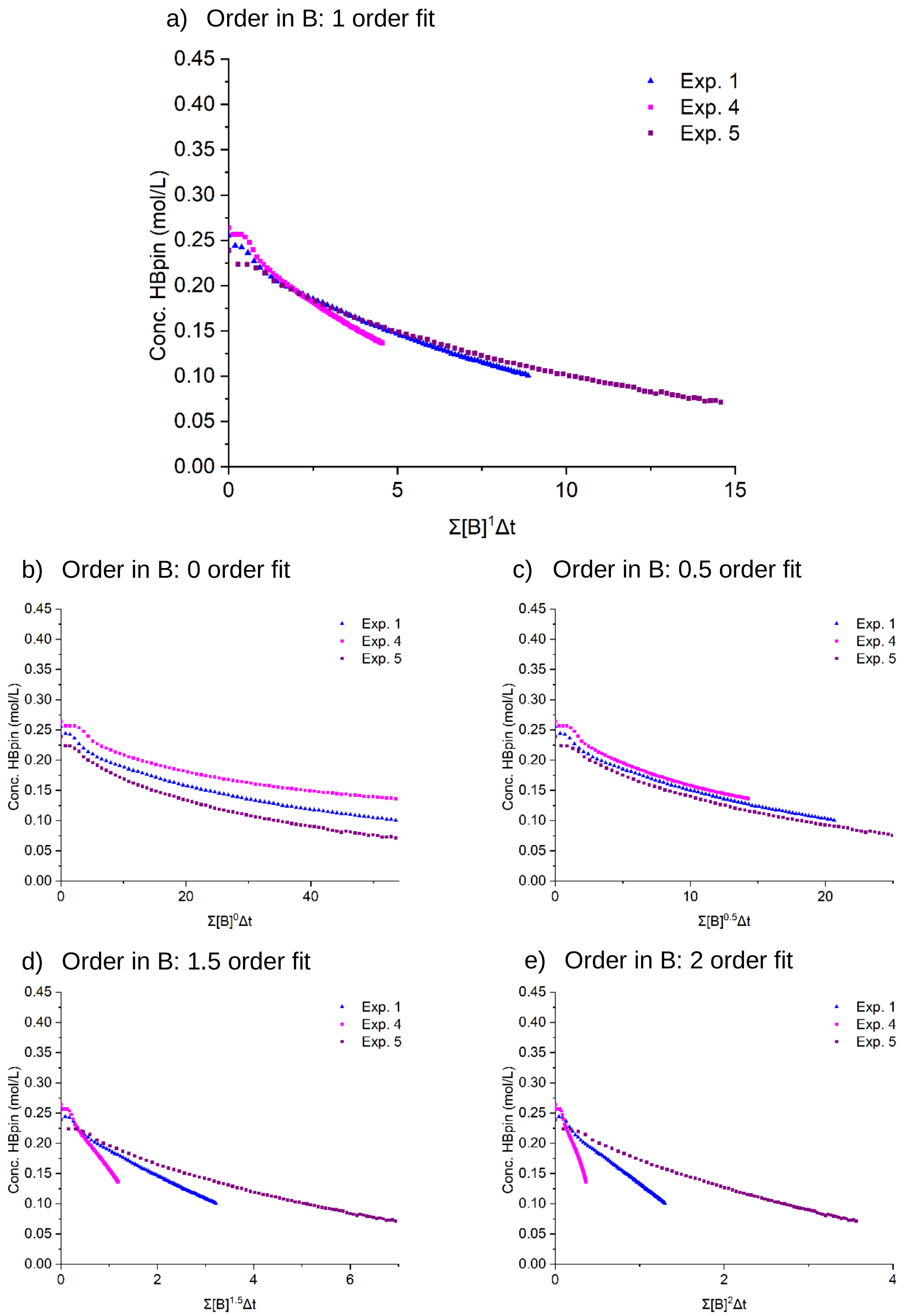

Figure S113. VTNA hydroboration benzaldehyde using HBpin as reducing agent and 0.1 mol% [Na(18c6)]$_2$**[2]** as catalyst. Graphs a, b, c, d, and e are the graphical representation of different orders in concentration HBpin ([B]) using the concentration of HBpin ([B]), obtained from the analysis.

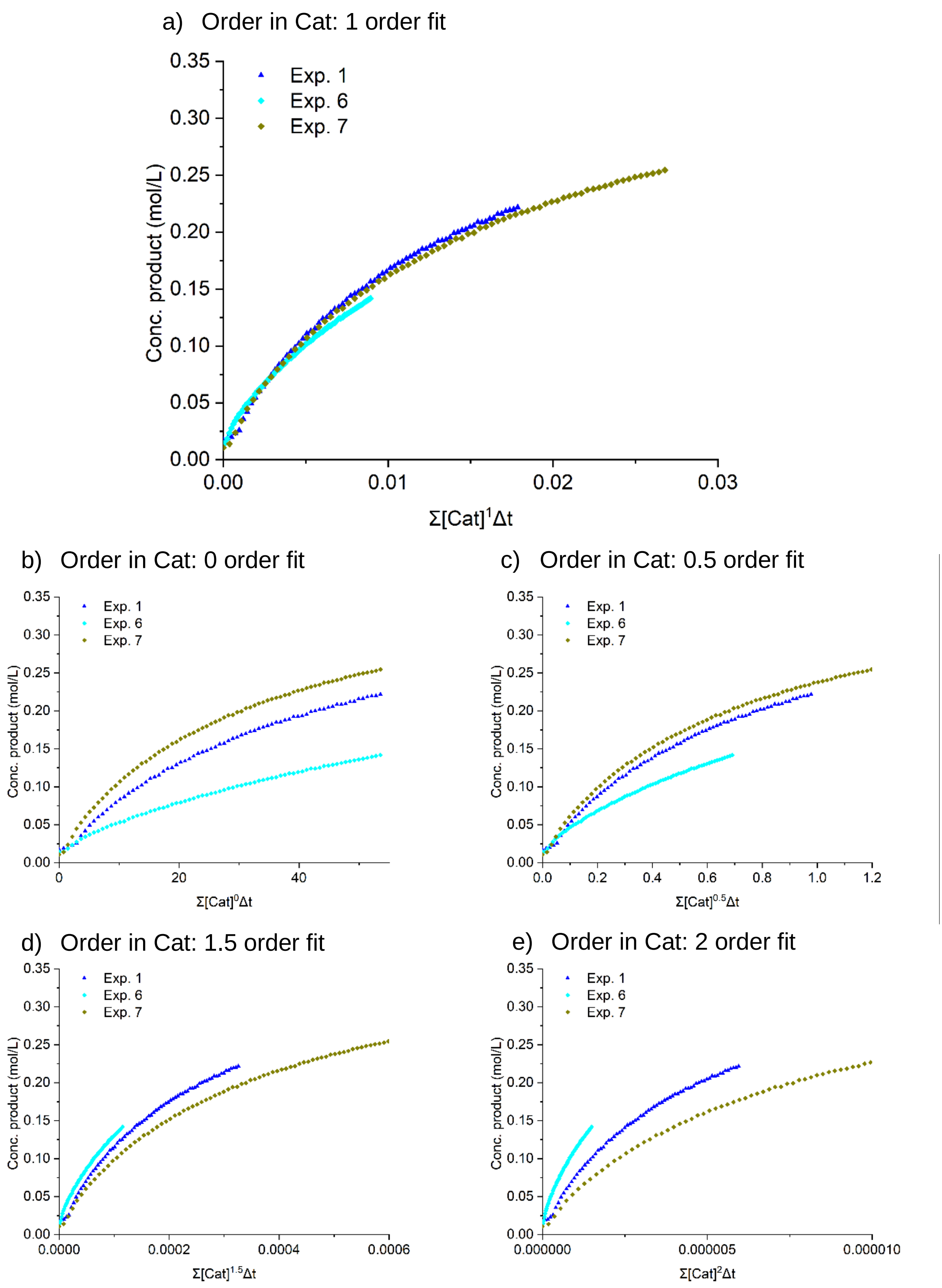

Figure S114. VTNA hydroboration benzaldehyde using HBpin as reducing agent and 0.1 mol% $[Na(18c6)]_2$[**2**] as catalyst. Graphs a, b, c, d, and e are the graphical representation of different orders in concentration catalyst ([Cat]) using the concentration of the product, obtained from the analysis.

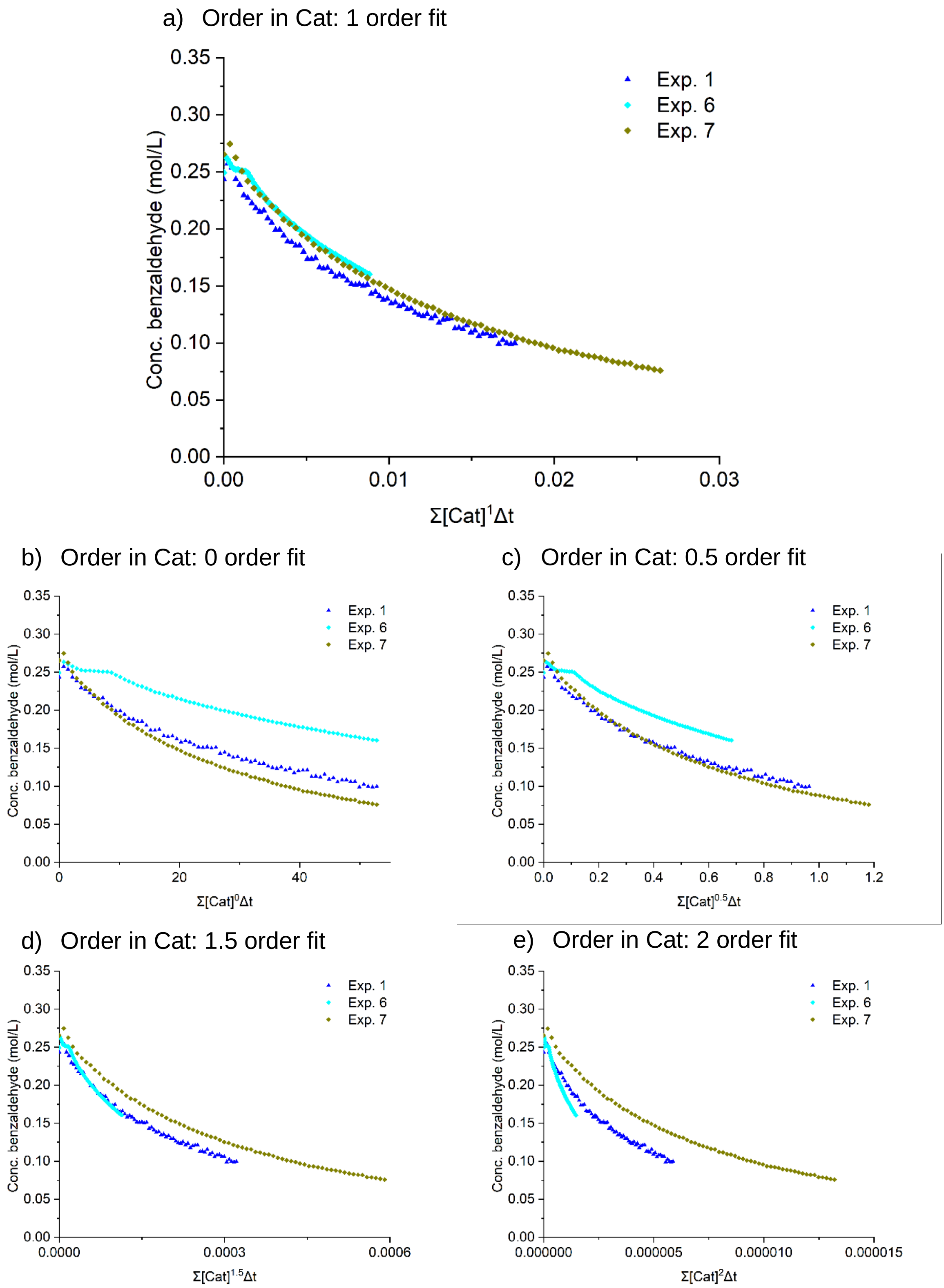

Figure S115. VTNA hydroboration benzaldehyde using HBpin as reducing agent and 0.1 mol% [Na(18c6)]$_2$[**2**] as catalyst. Graphs a, b, c, d, and e are the graphical representation of different orders in concentration catalyst ([Cat]) using the concentration of benzaldehyde ([A]), obtained from the analysis.

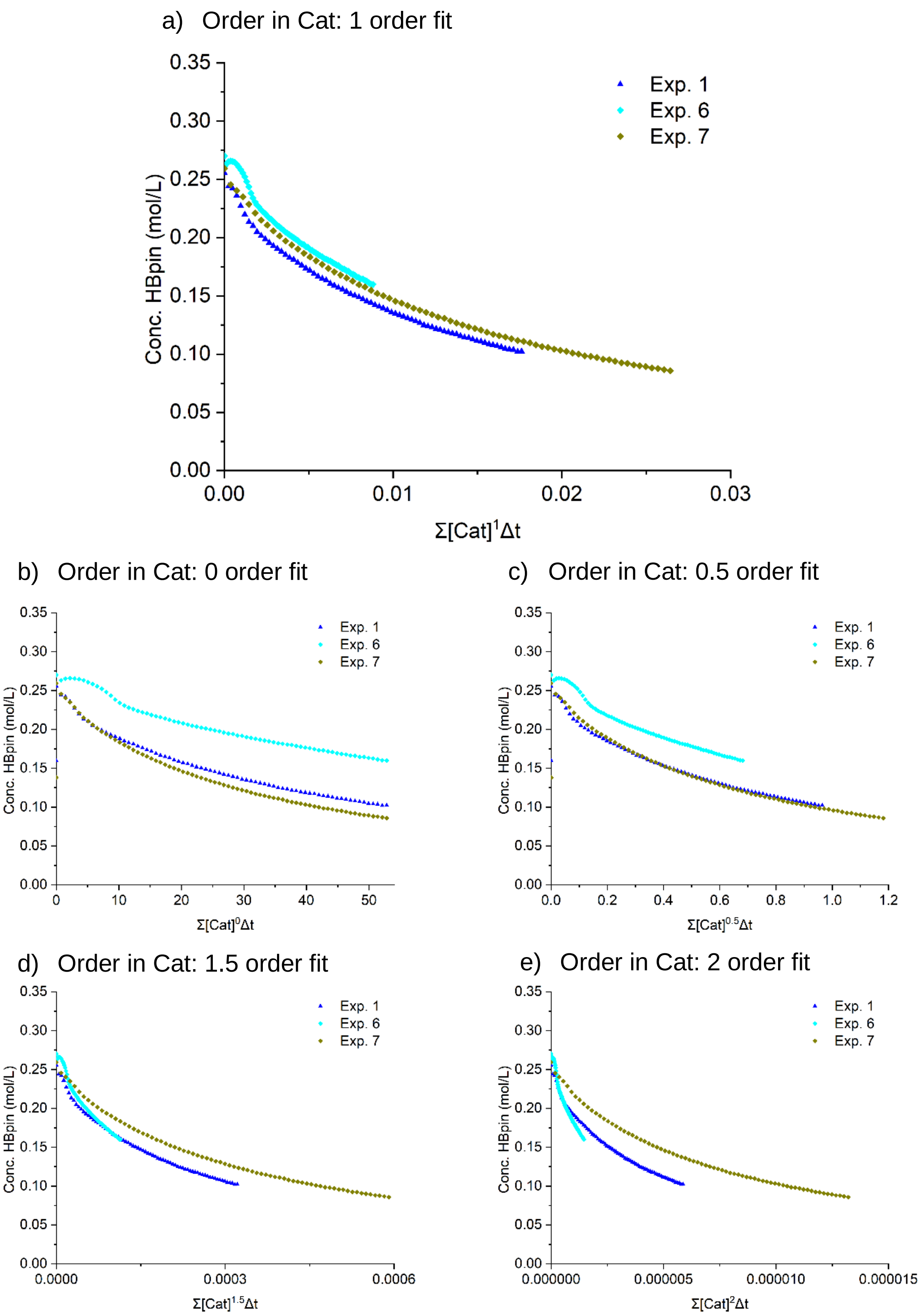

Figure S116. VTNA hydroboration benzaldehyde using HBpin as reducing agent and 0.1 mol% [Na(18c6)]$_2$[**2**] as catalyst. Graphs a, b, c, d, and e are the graphical representation of different orders in concentration catalyst ([Cat]) using the concentration of HBpin ([B]), obtained from the analysis.

6. Kinetic Isotope Effect

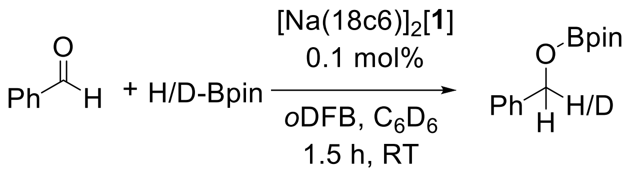

KIE = K_H/K_D

DBpin was prepared as a solution in C_6D_6 according to literature procedure.[30] Concentration of the DBpin was calculated by ^{1}H NMR spectroscopy using toluene as internal standard before use. The concentration of DBpin was determined to be 1.685 mol/L.

Benzaldehyde was used as a model substrate to investigate the mechanism of the CO_2 hydroboration. Mimicking conditions used in the hydroboration of CO_2. To a J Young NMR tube a solution of [Na(18c6)]$_2$[**1**] (0.2 mg, 0.22 µmol) in *o*DFB (400 µL) benzaldehyde (22 µL, 0.22 mmol) in *o*DFB, a solution of H/D-Bpin in C_6D_6 (0.22 mol in 130 µL C_6D_6) and toluene (25 µL, 0.24 mmol) was added. Reaction profiles were tracked by performing the reaction in a NMR spectrometer. Samples were loaded into the NMR spectrometer within 1.5-2.5 min, exact time was measured. Concentrations of the H/D-Bpin (^{1}H δ = 1.28 ppm) was calculated by integration of the ^{1}H NMR spectrum using toluene as an internal standard (^{1}H δ = 2.31 ppm). Following the concentration profiles, data was normalized against the initial substrate concentration. Reaction rates were derived from the plot of Ln($[B]_0/[B]_t$) vs time by using linear trendlines generated by Microsoft Excel software. The KIE was determined using the initial reaction rates (10% conversion) and determined to be 2.16.

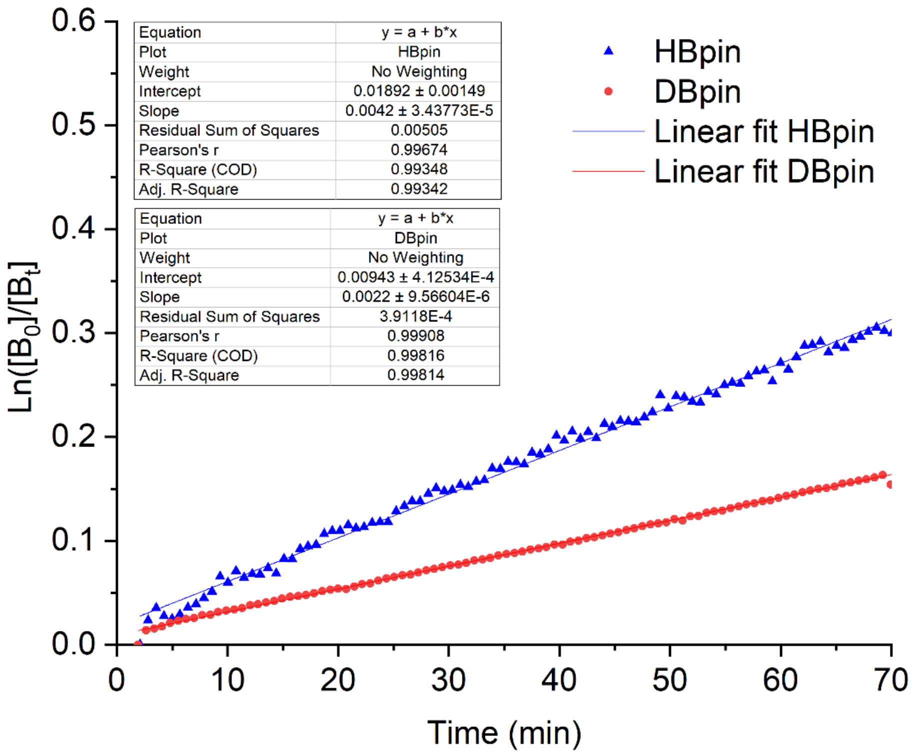

Figure S117. Determination of KIE using reaction rates of the full reaction profile.

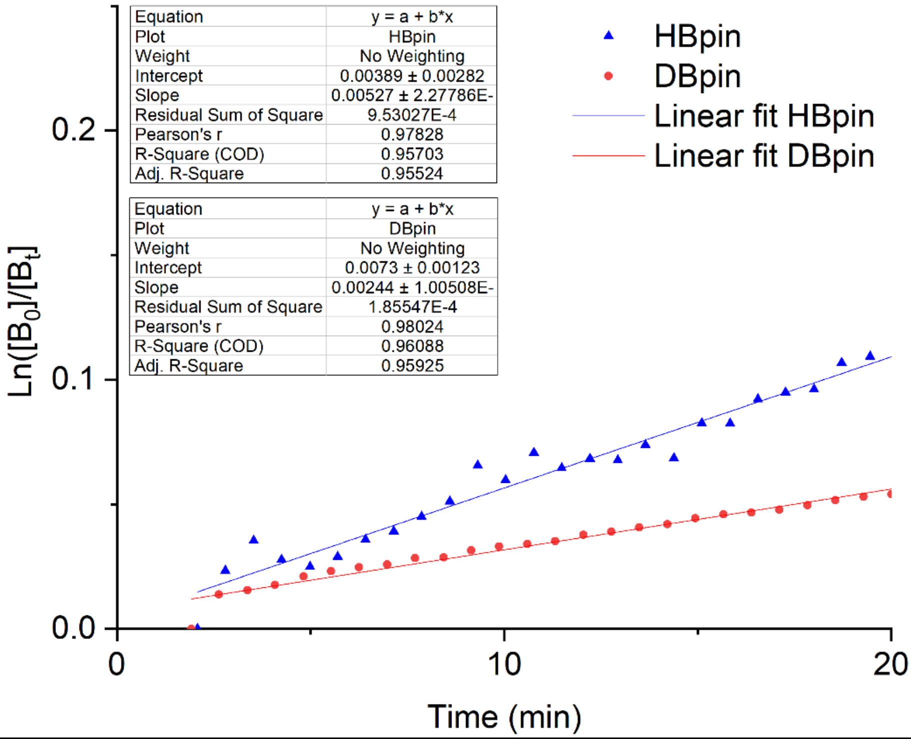

Figure S118. Determination of KIE using initial reaction rates.

7. Crystallography Table

Identification code	[Na(18c6)]$_2$[**2**]	[K(18c6)]$_2$[**3**]
Empirical formula	$C_{32}H_{66}AlNa_2O_{12}P_7$	$C_{32}H_{66}AlAs_7K_2O_{12}$
Formula weight	932.59	1272.46
Temperature/K	100.01	99.98(12)
Crystal system	triclinic	triclinic
Space group	P-1	P-1
a/Å	11.7559(4)	11.1937(17)
b/Å	14.0566(9)	17.759(3)
c/Å	16.2196(7)	26.344(5)
α/°	74.051(4)	104.725(14)
β/°	87.079(3)	96.415(13)
γ/°	66.652(5)	91.157(12)
Volume/Å^3	2361.0(2)	5026.7(14)
Z	2	4
ρ_{calc}g/cm^3	1.312	1.681
μ/mm^{-1}	3.238	4.828
F(000)	988.0	2544.0
Crystal size/mm^3	0.124 × 0.064 × 0.041	0.07 × 0.02 × 0.02
Radiation	Cu Kα (λ = 1.54184)	Mo Kα (λ = 0.71073)
2Θ range for data collection/°	5.68 to 152.652	3.474 to 35.998
Index ranges	-14 ≤ h ≤ 10, -17 ≤ k ≤ 16, -19 ≤ l ≤ 20	-9 ≤ h ≤ 9, -15 ≤ k ≤ 15, -22 ≤ l ≤ 22
Reflections collected	25302	14272
Independent reflections	9428 [R_{int} = 0.0474, R_{sigma} = 0.0597]	14272 [R_{int} = 0.1415, R_{sigma} = 0.3886]
Data/restraints/parameters	9428/0/521	14272/2321/1011
Goodness-of-fit on F^2	1.074	0.873
Final R indexes [I>=2σ (I)]	R_1 = 0.0495, wR_2 = 0.1299	R_1 = 0.0736, wR_2 = 0.1720
Final R indexes [all data]	R_1 = 0.0622, wR_2 = 0.1377	R_1 = 0.1431, wR_2 = 0.1937
Largest diff. peak/hole / e Å^{-3}	0.72/-0.59	1.10/-0.71
CCDC	2365860	2365861

A- or B-level alerts for $[K(18c6)]_2$[**3**] and justification.

THETM01_ALERT_3_A The value of sine(theta_max)/wavelength is less than 0.550
Calculated sin(theta_max)/wavelength = 0.4348
Author Response: Crystals of the sample were weakly diffracting. The data were truncated to the observable diffraction limit.

PLAT088_ALERT_3_B Poor Data / Parameter Ratio .... 6.84 Note
Author Response: Crystals of the sample were weakly diffracting. However, it was not deemed appropriate to model the atomic positions without anisotropic atomic displacement parameters. Appropriate atomic displacement parameter restraints have been applied to the model to mitigate against this poor data to parameter ratio.

PLAT242_ALERT_2_B Low 'MainMol' Ueq as Compared to Neighbors of K1D Check
Author Response: K1D is the central K+ in a clearly disordered 18crown6. However, the low resolution of the data precludes modelling of the disorder of this 18crown6

PLAT341_ALERT_3_B Low Bond Precision on C-C Bonds .... 0.04091 Ang.
Author Response: There is a large amount of residual noise in the electron density map. This lowers the precision of the C-C bonds. The validity of the model is not impinged by this.

8. Details of computational study

All density functional theory calculations were performed using the ORCA 5.0.4 program,[31] the r2SCAN-3c functional[32, 33] a bespoke mTZVPP basis set, and D4 and geometrical counterpoise (gCP) corrections.[34, 35] The influence of the solvent was modelled using the CPCM model with the following parameters for oDFB: Dielectric Constant (ε) = 14.26 and Refractive Index (*n*) = 1.443.[36, 37] All free energies include a concentration-induced correction of 1.89 kcal/mol to account for the change in standard state from gas phase (1 atm) to solution (1 mol/L).[38]

A sample input file is shown below.

Example of an ORCA input file for a ground state structure optimization:

```
# Ground State Optimization
! r2SCAN-3c
! Opt Freq
! NoPOP CPCM DefGrid3 #VerySlowConv

%geom
   MaxIter 200
   CoordSys Redundant_New
   ReducePrint True
   Convergence Tight
end

%scf
   MaxIter 150
   Convergence VeryTight
end

%cpcm
   Epsilon 14.26
   Refrac 1.443
end

* xyzfile -2 1 first.xyz
```

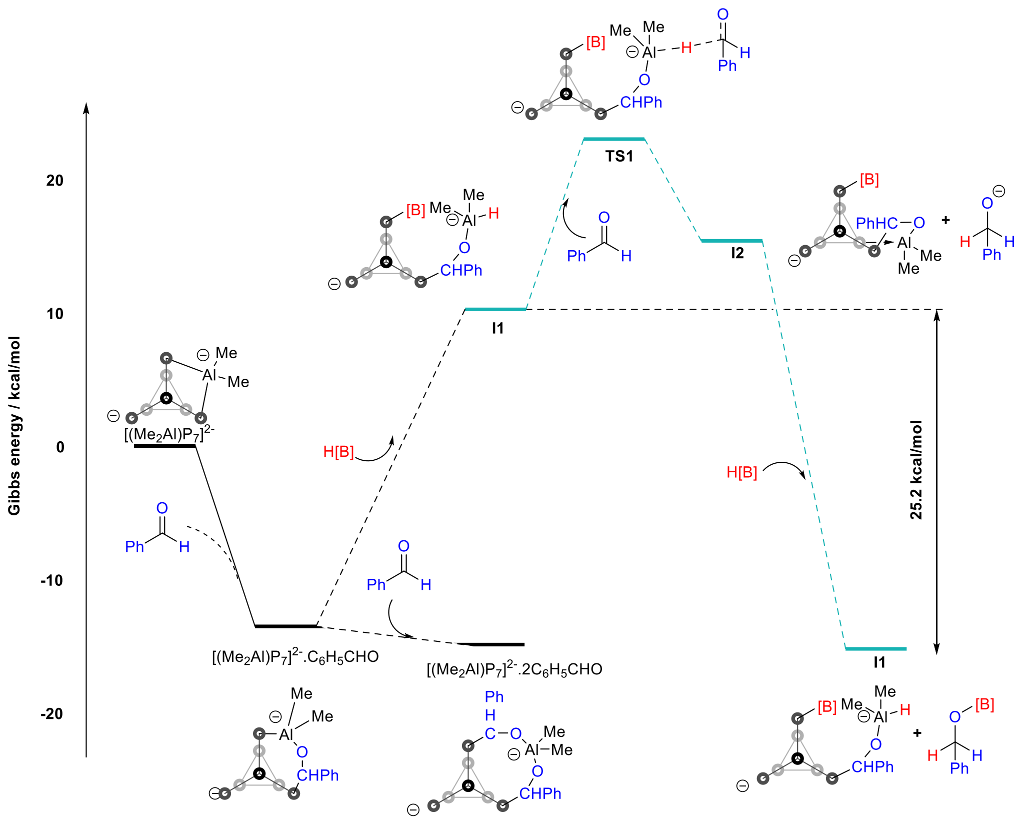

Figure S119. Gibbs energy surface for the proposed mechanism summarized in manuscript Figure 5

The pathway set out in Figure S119 is separated into a pre-catalytic phase, identified with black lines, and the catalytic cycle itself, shown with blue lines. The pre-catalytic phase involves binding one and then two molecules of benzaldehyde, the first very exergonically, the second with a fee energy close to zero. As an alternative to binding a second molecule of benzaldehyde, $[(Me_2Al)P_7]^{2-}$•C_6H_5CHO can also react with HBPin via insertion into the Al–P bond to form the aluminium hydride intermediate, **I1**. The overall process is endergonic (ΔG = +23.8 kcal/mol), indicating that the hydride will be present only in trace amounts. The hydride is then transferred to a further, unbound, molecule of C_6H_5CHO to form a free alkoxide (**I2**, ΔG = +5.2 kcal/mol), at which point the 3-coordinate Al center is stabilized by binding to the $[P_7]$ cluster. The alkoxide then binds to a further molecule of HBpin, driving transfer of the hydride to the Al center to reform **I1**, with concomitant generation of the product. We note that Li and co-workers have identified 4-membered ring transition states for the *trans*-metallation step which form the Al–H and B–O bonds in a concerted fashion, avoiding the release of free alkoxide.[39] We have not been able to locate such transition states here, probably because the neighbouring $[P_7]$ cluster provides an alternative mechanism to saturate the Al center during hydride loss. However, we do not rule out the possibility that such transition states may further lower the barrier if, for example, the initial B–H insertion was into the Al–O bond of $[(Me_2Al)P_7]^{2-}$•C_6H_5CHO, leaving the $AlMe_2H$ center bound directly to the $[P_7]$ unit. Investigations into these alternative pathways are ongoing.

9. References

1. Cicač-Hudi, M., *et al.* Direct Access to Inversely Polarized Phosphaalkenes from Elemental Phosphorus or Polyphosphides. *Eur. J. Inorg. Chem.* **2016**, 649–658 (2016).

2. Jobbins, W. D., van IJzendoorn, B., Vitorica-Yrezabal, I. J., Whitehead, G. F. S., Mehta, M. Reactivity of Tetrel Functionalized Heptapnictogen Clusters Towards Heteroallenes. *Dalton Trans.* **52**, 2384–2391 (2023).

3. Turbervill, R. S. P., Goicoechea, J. M. Hydrophosphination of Carbodiimides Using Protic Heptaphosphide Cages: A Unique Effect of the Bimodal Activity of Protonated Group 15 Zintl Ions. *Organometallics* **31**, 2452–2462 (2012).

4. Turbervill, R. S. P., Goicoechea, J. M. Hydropnictination Reactions of Carbodiimides and Isocyanates with Protonated Heptaphosphide and Heptaarsenide Zintl Ions. *Eur. J. Inorg. Chem.* **2014**, 1660–1668 (2014).

5. van IJzendoorn, B., Albawardi, S. F., Vitorica-Yrezabal, I. J., Whitehead, G. F. S., McGrady, J. E., Mehta, M. A Zintl Cluster for Transition Metal-Free Catalysis: C═O Bond Reductions. *J. Am. Chem. Soc.* **144**, 21213–21223 (2022).

6. Knapp, C., Zhou, B., Denning, M. S., Rees, N. H., Goicoechea, J. M. Reactivity Studies of Group 15 Zintl Ions Towards Homoleptic Post-Transition Metal Organometallics: A ‘Bottom-Up’ Approach to Bimetallic Molecular Clusters. *Dalton Trans.* **39**, 426–436 (2010).

7. CrysAlisPro Software System VxRC (2022).

8. Sheldrick, G. M. SHELXT-Integrated Space-Group and Crystal-Structure Determination. *Acta. Cryst.* **A71**, 3–8 (2015).

9. Dolomanov, O. V., Bourhis, L. J., Gildea, R. J., Howard, J. A. K., Puschmann, H. OLEX2: A Complete Structure Solution, Refinement and Analysis Program. *J. Appl. Cryst.* **42**, 339–341 (2009).

10. Blondiaux, E., Pouessel, J., Cantat, T. Carbon Dioxide Reduction to Methylamines Under Metal-Free Conditions. *Angew. Chem. Int. Ed.* **53**, 12186–12190 (2014).

11. Courtemanche, M.-A., Légaré, M.-A., Maron, L., Fontaine, F.-G.. A Highly Active Phosphine–Borane Organocatalyst for the Reduction of CO_2 to Methanol Using Hydroboranes. *J. Am. Chem. Soc.* **135**, 9326–9329 (2013).

12. Wang, T., Stephan, D. W. Phosphine Catalyzed Reduction of CO_2 with Boranes. *Chem. Commun.* **50**, 7007–7010 (2014).

13. von Wolff, N., Lefèvre, G., Berthet, J. C., Thuéry, P., Cantat, T. Implications of CO_2 Activation by Frustrated Lewis Pairs in the Catalytic Hydroboration of CO_2: A View Using N/Si^+ Frustrated Lewis Pairs. *ACS Catal.* **6**, 4526–4535 (2016).

14. Liu, L., Lo, S.-K., Smith, C., Goicoechea, J. M. Pincer-Supported Gallium Complexes for the Catalytic Hydroboration of Aldehydes, Ketones and Carbon Dioxide. *Chem. Eur. J.* **27**, 17379–17385 (2021).

15. Sau, S. C., Bhattacharjee, R., Vardhanapu, P. K., Vijaykumar, G., Datta, A., Mandal, S. K. Metal-Free Reduction of CO_2 to Methoxyborane under Ambient Conditions through Borondiformate Formation. *Angew. Chem. Int. Ed.* **55**, 15147–15151 (2016).

16. Das Neves Gomes, C., Blondiaux, E., Thuéry, P., Cantat, T. Metal-Free Reduction of CO_2 with Hydroboranes: Two Efficient Pathways at Play for the Reduction of CO_2 to Methanol. *Chem. Eur. J.* **20**, 7098–7106 (2014).

17. Yang, Y., Xu, M., Song, D. Organocatalysts with Carbon-Centered Activity for CO_2 Reduction with Boranes. *Chem. Commun.* **51**, 11293–11296 (2015).

18. Chia, C.-C.*, et al.* Aluminum-Hydride-Catalyzed Hydroboration of Carbon Dioxide. *Inorg. Chem.* **60**, 4569–4577 (2021).

19. Ramos, A., Antiñolo, A., Carrillo-Hermosilla, F., Fernández-Galán, R. $Ph_2PCH_2CH_2B(C_8H_{14})$ and Its Formaldehyde Adduct as Catalysts for the Reduction of CO_2 with Hydroboranes. *Inorg. Chem.* **59**, 9998–10012 (2020).

20. Chakraborty, S., Zhang, J., Krause, J. A., Guan, H. An Efficient Nickel Catalyst for the Reduction of Carbon Dioxide with a Borane. *J. Am. Chem. Soc.* **132**, 8872–8873 (2010).

21. Sánchez, P.*, et al.* Hydroboration of Carbon Dioxide with Catechol- and Pinacolborane using an Ir–CNP* Pincer Complex. Water Influence on the Catalytic Activity. *Dalton Trans.* **47**, 16766–16776 (2018).

22. Liu, T.*, et al.* Hydroboration of CO_2 Catalyzed by Bis(phosphinite) Pincer Ligated Nickel Thiolate Complexes. *Dalton Trans.* **46**, 4504–4509 (2017).

23. Erken, C.*, et al.* Manganese-Catalyzed Hydroboration of Carbon Dioxide and Other Challenging Carbonyl Groups. *Nat. Commun.* **9**, 4521 (2018).

24. Mukherjee, D., Wiegand, A.-K., Spaniol, T. P., Okuda, J. Zinc Hydridotriphenylborates Supported by a Neutral Macrocyclic Polyamine. *Dalton Trans.* **46**, 6183–6186 (2017).

25. Janes, T., Osten, K. M., Pantaleo, A., Yan, E., Yang, Y., Song, D. Insertion of CO_2 into the Carbon–Boron Bond of a Boronic Ester Ligand. *Chem. Commun.* **52**, 4148–4151 (2016).

26. Tamang, S. R., Findlater, M.. Cobalt Catalysed Reduction of CO_2 via Hydroboration. *Dalton Trans.* **47**, 8199–8203 (2018).

27. Aloisi, A., Berthet, J.-C., Genre, C., Thuéry, P., Cantat, T.. Complexes of the Tripodal Phosphine Ligands $PhSi(XPPh_2)_3$ (X = CH_2, O): Synthesis, Structure and Catalytic Activity in the Hydroboration of CO_2. *Dalton Trans.* **45**, 14774–14788 (2016).

28. Burés, J. A Simple Graphical Method to Determine the Order in Catalyst. *Angew. Chem. Int. Ed.* **55**, 2028–2031 (2016).

29. Nielsen, C. D. T., Burés, J. Visual Kinetic Analysis. *Chem. Sci.* **10**, 348–353 (2019).

30. Espinal-Viguri, M., Neale, S. E., Coles, N. T., Macgregor, S. A., Webster, R. L. Room Temperature Iron-Catalyzed Transfer Hydrogenation and Regioselective Deuteration of Carbon–Carbon Double Bonds. *J. Am. Chem. Soc.* **141**, 572–582 (2019).

31. Neese, F. Software update: The ORCA program system—Version 5.0. *WIREs Comput. Mol. Sci.* **12**, e1606 (2022).

32. Grimme, S., Hansen, A., Ehlert, S., Mewes J-M. r2SCAN-3c: A “Swiss Army Knife” Composite Electronic-Structure Method. *J. Chem. Phys.* **154**, 064103 (2021).

33. Furness, J. W., Kaplan, A. D., Ning, J., Perdew, J. P., Sun, J. Accurate and Numerically Efficient r2SCAN Meta-Generalized Gradient Approximation. *J. Phys. Chem. Lett.* **11**, 8208–8215 (2020).

34. Caldeweyher, E., *et al.* A Generally Applicable Atomic-Charge Dependent London Dispersion Correction. *J. Chem. Phys.* **150**, 154122 (2019).

35. Kruse, H., Grimme, S. A Geometrical Correction for the Inter- and Intra-Molecular Basis Set Superposition Error in Hartree-Fock and Density Functional Theory Calculations for Large Systems. *J. Chem. Phys.* **136**, 154101 (2012).

36. Barone, V., Cossi, M. Quantum Calculation of Molecular Energies and Energy Gradients in Solution by a Conductor Solvent Model. *J. Phys. Chem. A* **102**, 1995–2001 (1998).

37. Laurence, C., Nicolet, P., Dalati, M. T., Abboud, J.-L. M., Notario, R. The Empirical Treatment of Solvent-Solute Interactions: 15 Years of π^*. *J. Phys. Chem.* **98**, 5807–5816 (1994).

38. Bursch, M., Mewes, J.-M., Hansen, A., Grimme, S.. Best-Practice DFT Protocols for Basic Molecular Computational Chemistry. *Angew. Chem. Int. Ed.* **61**, e202205735 (2022).

39. Li, C.-Q., Leng, G., Li, W. Hydroboration of Carbon Dioxide with Pinacolborane Catalyzed by Various Aluminum Hydrides: A Comparative Mechanistic Study. *Cat. Sci. Tech.* **12**, 6129–6141 (2022).
