# Supplementary material for: Transforming carbon dioxide into a methanol surrogate using modular transition metal-free Zintl ions
Source: Nat Commun. 2024 Nov 19;15:10030. doi: 10.1038/s41467-024-54277-z (PMC11576849; doi:10.1038/s41467-024-54277-z)
Supplement: Supplementary file 2 — Description of Additional Supplementary Files [file 41467_2024_54277_MOESM2_ESM.pdf]

Description of Additional Supplementary Files

File name: Supplementary Data 1
Description: DFT Coordinates. In here the calculated coordinates from the DFT studies are given.

File name: Supplementary Data 2
Description: Kinetic and recycling data. In here all the data associated with the VTNA kinetic analysis and recyclability studies are given.
